# One-Pot Sequence of Staudinger/aza-Wittig/Castagnoli–Cushman Reactions Provides Facile Access to Novel Natural-like Polycyclic Ring Systems

**DOI:** 10.3390/molecules27238130

**Published:** 2022-11-22

**Authors:** Rodion Lebedev, Dmitry Dar’in, Grigory Kantin, Olga Bakulina, Mikhail Krasavin

**Affiliations:** 1 Institute of Chemistry, Saint Petersburg State University, 26 Universitetskii Prospect, Peterhof 198504, Russia; 2 Institute of Living Systems, Immanuel Kant Baltic Federal University, Kaliningrad 236041, Russia

**Keywords:** Staudinger/aza-Wittig, Castagnoli–Cushman, imine, homophthalic anhydride, lactam, azide

## Abstract

Realization of the one-pot Staudinger/aza-Wittig/Castagnoli–Cushman reaction sequence for a series of azido aldehydes and homophthalic anhydrides is described. The reaction proceeded at room temperature and delivered novel polyheterocycles related to the natural product realm in high yields and high diastereoselectivity. The methodology has been extended to three other cyclic anhydrides. These further unravel the potential of the Castagnoli–Cushman reaction in generating polyheterocyclic molecular scaffolds.

## 1. Introduction

Molecular scaffolds are the foundation of a compound’s biological activity [1]. This notion and its importance for drug discovery and the goal of generating drugs leads free from intellectual property liabilities [2] and fuels efforts in synthetic method development defined by Aktitopoulou-Zanze at Abbott as scaffold-oriented synthesis [3]. The major aim of scaffold-oriented synthesis is the deduction of synthetic pathways to novel ring systems, particularly polyheterocyclic ones, taking into account that bioactive compound [4] and natural product [5,6,7] chemical space is heavily populated with polyheterocycles.

**Figure 1:**
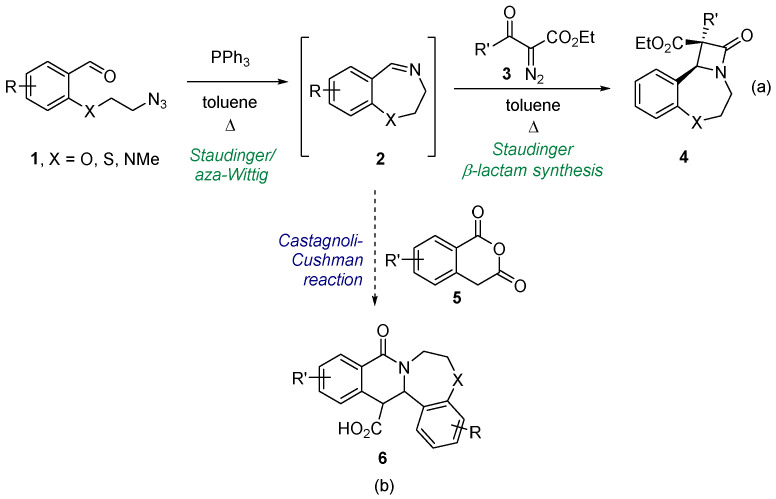
Synthesis of cyclic imines **2** and their involvement in ring-forming processes: (**a**) recently reported and (**b**) investigated in this work.

Involvement of a cyclic starting material in a ring-forming process is a proven way to create a polycyclic framework [8]. Such processes are often associated with the creation of new, sometimes multiple, stereocenters, so relying on or discovering diastereoselective approaches to the new cycle formation is key. Recently, we developed a convenient synthetic protocol to transform azide-bearing benzaldehydes **1** into cyclic seven-membered imines **2** via the Staudinger reaction with triphenyl phosphine followed by an intramolecular aza-Wittig reaction [9]. Imines **2** were involved in the Staudinger β-lactam synthesis with ketenes generated from diazo compounds **3**. As the result, tricyclic β-lactams **4** fused with tetrahydrothia-, oxa- and diazepine rings were obtained with high diastereoselectivity. Encouraged by this success, we pondered if the same cyclic imine substrates could be coupled to other ring-forming reactions. Our positive recent experience [10,11,12] with the imines (cyclic and acyclic) reacting with cyclic anhydrides via the formal [4+2] cycloaddition dubbed the Castagnoli–Cushman reaction (CCR) [13,14] prompted us to investigate the possibility of employing imines **2** as substrates for the CCR. As the starting point for this investigation, we considered reacting them with homophthalic anhydrides (HPA, **5**), one of the most popular and reactive anhydrides that have been involved in the CCR [15,16,17]. This, we reasoned, would provide access to a tetracyclic 6-6-7-6 ring system **6** containing two new stereogenic centers. Considering that the CCR quite often proceeds diastereoselectively [18,19,20,21,22], the choice of the CCR as the means to create the new polyheterocyclic scaffold **6** from imines **2** is quite justified (Figure 1). Herein, we present the results obtained in the course of realizing the synthetic strategy outlined above.

## 2. Results and Discussion

**Scheme 1:**
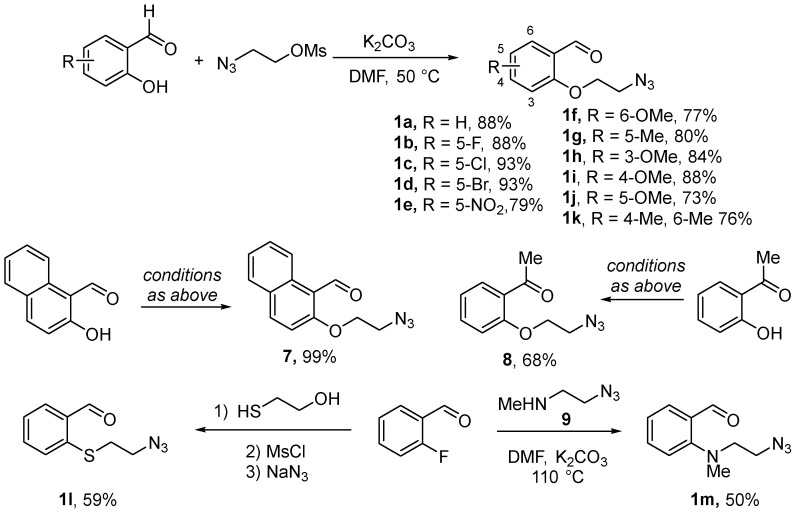
Preparation of azido aldehyde and ketone substrates for the Staudinger/aza-Wittig cyclization.

*O*-linked aldehydes **1** required for the realization of the planned approach were synthesized from *o*-hydroxybenzaldehydes via alkylation with 2-azidoethyl mesylate as described elsewhere [9]. Eleven *O*-(2-azidoethyl) substrates **1a**–**k** were obtained in good to excellent yields. In addition to these, naphthalene-templated *O*-linked azido aldehyde **7** and azido ketone **8** were obtained, also in high yields, from the respective phenolic starting materials. *S*-linked azido aldehyde **1l** was prepared via nucleophilic aromatic substitution of fluorine in *o*-fluorobenzaldehyde with 2-hydroxyethyl thiol followed by mesylation of the hydroxyl group and nucleophilic displacement of mesylate with azide. *N*-linked azido aldehyde **1m** was prepared in a similar manner from *o*-fluorobenzaldehyde using 2-azidoethyl methylamine (**9**) as the nucleophilic agent [9] (Scheme 1).

**Scheme 2:**
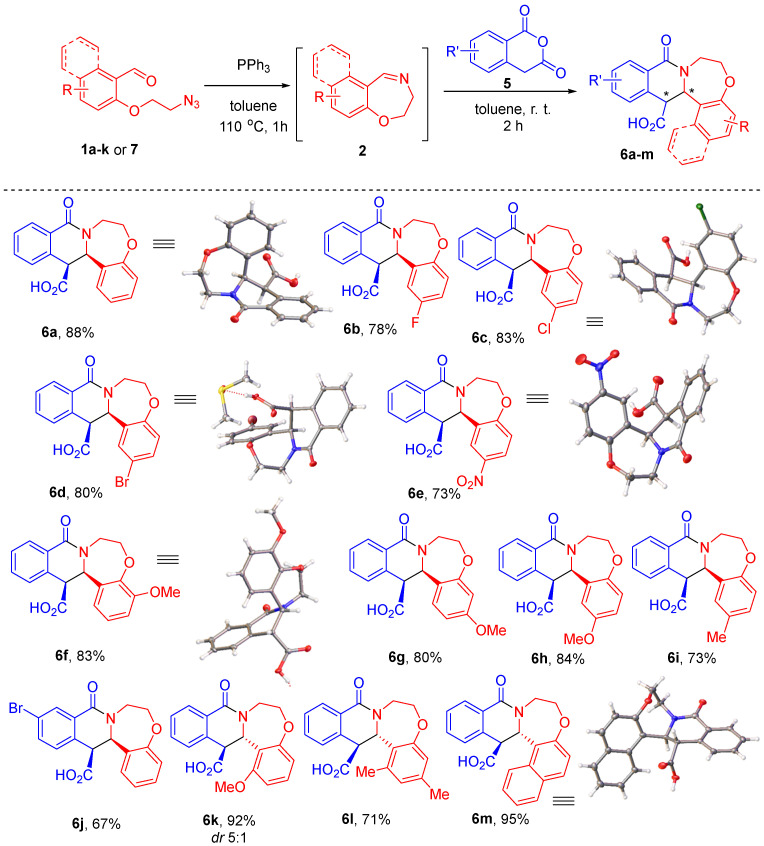
The one-pot Staudinger/aza-Wittig generation of imines **2** followed by the CCR with HPA.

Considering that cyclic imines **2** can be generated on heating starting materials such as **1a**–**k** or **7** in toluene and used, without the need for isolation, in the subsequent transformation, provided the latter can be conducted also in toluene (as was the case with the synthesis of b-lactams **4** [9]), we opted to attempt to couple the CCR of HPA to the preparation of **2** this way as well. The CCR of HPA can be conducted in a range of different solvents [16], depending on the solubility of the starting material (HPA itself not being the limiting factor). Fortunately, imines **2** do not precipitate from toluene even at room temperature, so after their formation and cooling the solution to ambient temperature, we added HPA (**5**) directly to the solution of imines **2** and continued the reaction with no heating. To our delight, in 2 h the starting materials (**2** and HPA) were fully consumed and a thick precipitate of products **6** formed. The latter were filtered off, washed with ether and air-dried. NMR analysis of the products **6a**–**m** thus isolated (in 67–95% yield) confirmed their identity and purity as greater than 95%, whereas they were not found in the filtrate. In the majority of instances (except for **6k**), only one diastereomer was observed in the ^1^H NMR spectra (see Supplementary Materials), thus confirming an excellent case of fully diastereoselective CCR (Scheme 2).

The relative stereochemistry of compounds **6a**–**j** was deemed to be *cis* based on the similarity of their NMR spectra and the single-crystal X-ray structures which were obtained for compounds **6a**, **6c** and **6d**–**f**. The characteristic features of the ^1^H NMR spectra of compounds **6a**–**j** are the significant broadening of the signals corresponding to the ex-imine aromatic portion and the bis-methylene linker (presumably, due to the conformational behavior of the seven-membered ring) as well as the vicinal ^3^*J*_HH_ coupling constants of the signals coming from the methine protons at the asymmetric carbon atoms. In contrast, adducts **6k**–**m** derived from aldehydes **1** where the position of the aromatic ring *ortho* to the carbonyl group was substituted displayed a different set of ^1^H NMR characteristics (no broadening and different methine ^3^*J*_HH_ coupling constants) and were deemed to be *trans*-configured. Such a stereochemical outcome is not unexpected since the respective *cis*-configured products would be sterically congested. This stereochemistry assignment was further supported by the single-crystal X-ray structure obtained for compound **6m** (see Supplementary Materials).

**Scheme 3:**
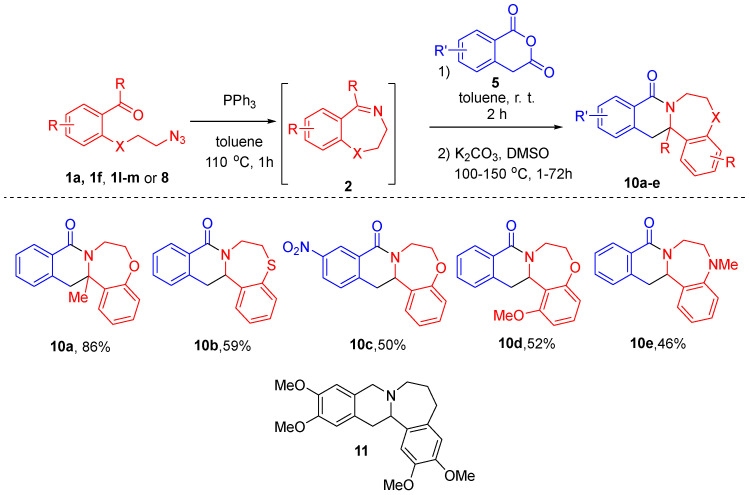
Staudinger/aza-Wittig/CCR sequence followed by decarboxylation.

Unfortunately, involvement of imines **2** derived from azido aldehydes **1l**–**m** and **8** in the reaction with HPA did not permit isolation of pure products **6,** although it was successful in terms of starting material conversion. Likewise, isolation of the products from the reactions of 7-nitro-HPA was problematic. However, it was possible to isolate products of decarboxylation of these impure carboxylic acids. After performing the Staudinger/aza-Wittig sequence and reacting starting materials **1a**, **1f**, **1l**–**m** or **8** with HPA (**5**), toluene was replaced with DMSO. Decarboxylation was performed at 100–150 °C in the presence of potassium carbonate. Tetracycles **10a-e** were isolated in good to excellent yields over three steps. Notably, these compounds are hetero des-oxo analogs of natural product-related B-homoxylopinin (**11**), first reported in 1976 [23], whose congeners displayed histone acetyltransferase inhibitory activity [24] (Scheme 3).

**Scheme 4:**
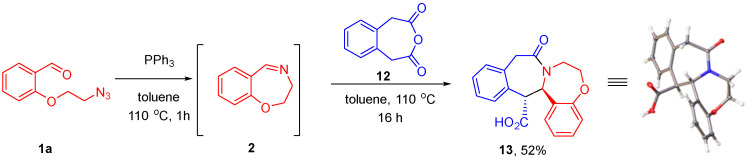
Involvement of *o*-phenylenediacetic acid anhydride in the Staudinger/aza-Wittig/CCR reaction sequence.

Having successfully realized our synthetic plan with HPA, we became interested in extending the scope of this approach to other cyclic anhydrides. *o*-Phenylenediacetic acid anhydride (**12**) was first introduced as a reagent for the CCR in 2017, providing an entry into ε-lactams. It was shown to be less reactive compared to HPA [25]. In line with this observation, the reaction of **12** with cyclic imine **2** generated from azido aldehyde **1a** required heating at 110 °C for 16 h to go to completion. The product of this transformation (**13**) containing a novel 6-7-7-6 polyheterocyclic ring system was isolated in a 52% yield by simple filtration as a single diastereomer, which was shown to be *trans*-configured by single-crystal X-ray analysis (see Supplementary Materials) (Scheme 4).

**Scheme 5:**
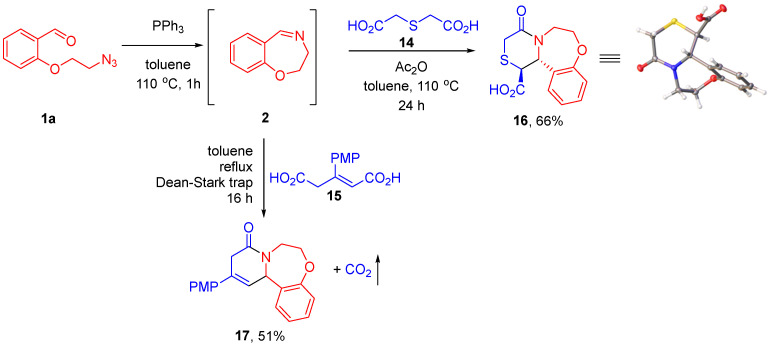
Staudinger/aza-Wittig/CCR reaction sequence conducted with dicarboxylic acids via in situ cyclodehydration.

CCR can be conveniently performed with the use of dicarboxylic acids in lieu of the respective cyclic anhydrides generated from diacids using dehydrating agents [26,27,28] or more straightforward cyclodehydration by means of azeotropic removal of water [11,29]. We chose to investigate the Staudinger/aza-Wittig/CCR reaction sequence for two dicarboxylic acids—thiodiglycolic (**14**) and *p*-methoxyphenyl-substituted glutaconic acid (**15**). After generation of imine **2** from azido aldehyde **1a**, diacid **14** and acetic anhydride (serving as the dehydrating agent) were added and the process was further conducted at 110 °C for 24 h. The CCR product **16** was obtained in a respectable 66% yield, with no need for chromatographic purification, as a single diastereomer, which was shown to be *trans*-configured by single-crystal X-ray analysis (see Supplementary Materials). Glutaconic acid **15** was cyclodehydrated by means of azeotropic removal of water. The CCR in this case was conducted in refluxing toluene and, as expected [11,30], was accompanied by decarboxylation and transposition of the double bond. The respective tricyclic adduct **17** was isolated in a 51% yield (Scheme 5).

**Scheme 6:**
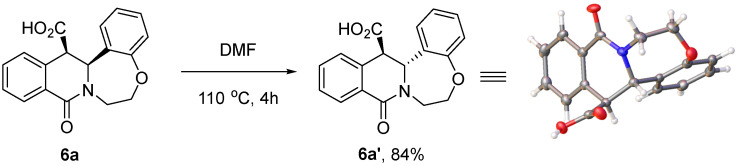
Thermal *cis* → *trans* isomerization of compound **6a**.

Finally, intrigued by the opposite stereochemical outcome of the reaction sequence under investigation in the case of products **6a**–**j** and **6k**–**m**, we reasoned that the *cis*-isomers of **6a**–**j** might signify the kinetic outcome of the CCR, while *trans*-configured products **6k**–**m** obtained from more sterically demanding substrates **2** are likely to be thermodynamic adducts. If such an interpretation is correct, it should be possible to isomerize the kinetic *cis*-configured tetracycles **6** to their *trans* counterparts by thermal activation or otherwise. This was indeed demonstrated for compound **6a**. On heating at 110 °C in dimethylformamide for 4 h, it was completely transformed to its *trans*-isomer **6a′,** which was isolated in an 84% yield. The relative stereochemistry of **6a′** was confirmed by single-crystal X-ray crystallography (Scheme 6).

## 3. Conclusions

We have described the realization of the one-pot Staudinger/aza-Wittig/Castagnoli–Cushman reaction sequence for a series of azido aldehydes and homophthalic anhydrides. The reaction proceeded at room temperature and delivered novel polyheterocycles related to the natural product realm in high yields and high diastereoselectivity. Certain substrates presented difficulty in terms of isolation of the resulting tetracyclic products, which can be overcome by thermal decarboxylation of the carboxylic acid Castagnoli–Cushman adduct in the presence of potassium carbonate. The methodology has been extended to three other cyclic anhydrides, two of which were generated from the respective dicarboxylic acids in situ. The *cis*-configured Castagnoli–Cushman adducts are kinetic products and can be thermally isomerized to their *trans* counterparts. Collectively, these findings substantially expand the scope of the Castagnoli–Cushman reaction in terms of the imine source and the molecular scaffold accessible via applying this ring-forming reaction to cyclic imines.

## 4. Materials and Methods

### 4.1. General Information

All commercial reagents were used without purification. NMR spectra were recorded using a Bruker Avance III spectrometer in CDCl_3_ (^1^H: 400.13 MHz; ^13^C: 100.61 MHz; ^19^F: 376.50 MHz); chemical shifts are reported as parts per million (*δ*, ppm); the residual solvent peak (CHCl_3_ or DMSO-*d*_6_) was used as the internal standard: 7.28 or 2.51 for ^1^H and 77.07 or 40.00 ppm for ^13^C; multiplicities are abbreviated as follows: s = singlet, d = doublet, t = triplet, q = quartet, m = multiplet, br = broad, dd = doublet of doublets, dt = doublet of triplets, ddd = doublet/doublets of doublets; coupling constants, *J*, are reported in Hz. Mass spectra were recorded using a Bruker microTOF spectrometer (ionization by electrospray, positive ions detection). Melting points were determined in open capillary tubes on a Stuart SMP50 Automatic Melting Point Apparatus. Analytical thin-layer chromatography was carried out on UV-254 silica gel plates using appropriate eluents. Compounds were visualized with short-wavelength UV light. Column chromatography was performed using silica gel Merk grade 60 (0.040−0.063 mm) 230−400 mesh. All reactions were conducted in the atmosphere of argon.

### 4.2. Preparation of Azides ***1***,***7***,***8*** and Their Precursors

#### 4.2.1. 2-Azidoethan-1-ol

This compound was obtained according to a slightly modified literature procedure [31]**.** Sodium azide (5.85 g, 90 mmol, 3.0 eq.) was added to a solution of 2-bromoethan-1-ol (3.75 g, 30 mmol, 1.0 eq.) in water (30 mL). The mixture was stirred overnight at 80 °C. The reaction solution was extracted with diethyl ether. Combined organic layers were dried over anhydrous Na_2_SO_4_ and the solvent was removed under reduced pressure to give 2-azidoethan-1-ol (2.5 g, 96%) as a colorless oil. ^1^H NMR (400 MHz, CDCl_3_) δ 3.79 (t, *J* = 5.0 Hz, 2H), 3.45 (t, *J* = 5.0 Hz, 2H), 2.22 (s, *br*, 1H), in accordance with previously obtained literature data.

#### 4.2.2. 2-Azidoethyl Methanesulfonate

This compound was obtained according to a slightly modified literature procedure [32]. Methanesulfonyl chloride (3.6 g, 31.39 mmol. 1.1 eq) was added to a solution of 2-azidoethan-1-ol (2.485 g, 28.537 mmol, 1.0 eq) and triethylamine (3.75 g, 1.3 eq) in DCM (57 mL) at 0 °C and the mixture was stirred for 30 min. One volume of saturated NaHCO_3_ solution was added and the mixture was stirred for 30 min. The organic layer was separated and the aqueous layer was extracted with DCM. Combined organic layers were washed with brine, dried over anhydrous Na_2_SO_4_ and the solvent was removed under reduced pressure to give 2-azidoethyl methanesulfonate (3.94 g, 84%) as a colorless oil. ^1^H NMR (400 MHz, CDCl_3_) δ 4.37 (t, *J* = 5.0 Hz, 2H), 3.62 (t, *J* = 5.0 Hz, 2H), 3.11 (s, 3H), in accordance with previously obtained literature data [33].

### 4.3. General Procedure for Synthesis of ***1a***–***1k***, ***7***, ***8*** (GP1)

Corresponding salicylic aldehyde (1.05 mmol, 1.05 eq), 2-azidoethyl methanesulfonate (0.165 g, 1 mmol, 1 eq) and K_2_CO_3_ (0.145 g, 1.05 mmol, 1 eq) were mixed in DMF (3 mL) and stirred at 50 °C. Reaction progress was controlled by TLC. The solvent of the resulting mixture was evaporated, and the residue was diluted with water and extracted with DCM. Combined organic layers were washed with NaOH (3%), water, brine, dried over anhydrous Na_2_SO_4_ and concentrated in vacuo to give corresponding 2-(2-azidoethoxy)benzaldehyde. 

*2-(2-Azidoethoxy)*benzaldehyde (**1a**), Yield: 168 mg (88%). Yellow amorphous solid. ^1^H NMR (400 MHz, CDCl_3_) δ 10.54 (s, 1H), 7.89 (dd, *J* = 7.7, 1.4 Hz, 1H), 7.64–7.50 (m, 1H), 7.10 (t, *J* = 7.9 Hz, 1H), 7.00 (d, *J* = 7.9 Hz, 1H), 4.29 (t, *J* = 4.9 Hz, 2H), 3.71 (t, *J* = 4.9 Hz, 2H). ^13^C NMR (101 MHz, CDCl_3_) δ 189.3, 160.4, 135.9, 128.6, 125.1, 121.5, 112.4, 67.5, 50.2. HRMS (ESI), *m/z* calcd. for C_9_H_10_N_3_O_2_ [M + H]^+^ 192.0773; found 192.0772. 

*2-(2-Azidoethoxy)-5-fluorobenzaldehyde* (**1b**), Yield: 184 mg (88%). Brown amorphous solid. ^1^H NMR (400 MHz, CDCl_3_) δ 10.46 (d, *J* = 3.1 Hz, 1H), 7.54 (dd, *J* = 8.2, 3.3 Hz, 1H), 7.32–7.23 (m, 1H), 6.97 (dd, *J* = 9.1, 3.8 Hz, 1H), 4.26 (d, *J* = 4.9 Hz, 2H), 3.69 (t, *J* = 4.9 Hz, 2H). ^13^C NMR (101 MHz, CDCl_3_) δ 188.2 (d, C-F, ^4^*J*_C-F_ = 1.5 Hz), 157.3 (d, C-F, ^1^*J*_C-F_ = 242.5 Hz), 156.7 (d, C-F, ^3^*J*_C-F_ = 2.0 Hz), 125.9 (d, C-F, ^3^*J*_C-F_ = 6.0 Hz), 122.4 (d, C-F, ^2^*J*_C-F_ = 24.0 Hz), 114.3 (d, C-F, ^2^*J*_C-F_ = 31.9 Hz), 114.2, 68.2, 50.2. ^19^F NMR (376.5 MHz, CDCl_3_) δ -121.32. HRMS (ESI), *m/z* calcd. for C_9_H_8_N_3_NaO_2_F [M + Na]^+^ 232.0498; found 232.0495. 

*2-(2-Azidoethoxy)-5-chlorobenzaldehyde* (**1c**), Yield: 210 mg (93%). Yellow amorphous solid. ^1^H NMR (400 MHz, CDCl_3_) 10.46 (s, 1H), 7.83 (d, *J* = 2.7 Hz, 1H), 7.52 (dd, *J* = 8.9, 2.7 Hz, 1H), 6.96 (d, *J* = 8.9 Hz, 1H), 4.28 (t, *J* = 4.9 Hz, 2H), 3.71 (t, *J* = 4.9 Hz, 2H). ^13^C NMR (101 MHz, CDCl_3_) δ 188.0, 158.8, 135.3, 128.2, 127.2, 126.0, 114.0, 67.9, 50.1. HRMS (ESI), *m/z* calcd. for C_9_H_8_ClN_3_NaO_2_ [M + Na]^+^ 248.0197; found 248.0196.

*2-(2-Azidoethoxy)-5-bromobenzaldehyde* (**1d**), Yield: 250 mg (93%). Yellow amorphous solid. ^1^H NMR (400 MHz, CDCl_3_) δ 10.45 (s, 1H), 7.98 (d, *J* = 2.6 Hz, 1H), 7.66 (dd, *J* = 8.8, 2.6 Hz, 1H), 6.90 (d, *J* = 8.8 Hz, 1H), 4.27 (t, *J* = 4.9 Hz, 2H), 3.71 (t, *J* = 4.9 Hz, 2H). ^13^C NMR (101 MHz, CDCl_3_) δ 187.9, 159.3, 138.2, 131.2, 126.4, 114.4, 67.9, 50.1. HRMS (ESI), *m/z* calcd. for C_9_H_9_N_3_O_2_Br [M + H]^+^ 269.9873; found 269.9878.

*2-(2-Azidoethoxy)-5-nitrobenzaldehyde* (**1e**), Yield: 187 mg (79%). Yellow amorphous solid. ^1^H NMR (400 MHz, CDCl_3_) δ 10.51 (s, 1H), 8.75 (d, *J* = 2.9 Hz, 1H), 8.46 (dd, *J* = 9.2, 2.9 Hz, 1H), 7.14 (d, *J* = 9.2 Hz, 1H), 4.42 (t, *J* = 4.9 Hz, 2H), 3.79 (t, *J* = 4.9 Hz, 2H). ^13^C NMR (101 MHz, CDCl_3_) δ 187.0, 164.1, 142.1, 130.6, 124.9, 124.7, 112.9, 68.5, 49.9. HRMS (ESI), *m/z* calcd. for C_9_H_9_N_4_O_4_ [M + H]^+^ 237.0618; found 237.0624.

*2-(2-Azidoethoxy)-6-methoxybenzaldehyde* (**1f**), Yield: 177 mg (77%). Brown oil. ^1^H NMR (400 MHz, CDCl_3_) δ 10.53 (s, 1H), 7.45 (t, *J* = 8.5 Hz, 1H), 6.64 (d, *J* = 8.5 Hz, 1H), 6.57 (d, *J* = 8.5 Hz, 1H), 4.22 (t, *J* = 5.0 Hz, 2H), 3.91 (s, 3H), 3.67 (t, *J* = 5.0 Hz, 2H). ^13^C NMR (101 MHz, CDCl_3_) δ 189.0, 161.8, 161.0, 135.7, 114.9, 104.9, 104.8, 67.8, 56.1, 50.1. HRMS (ESI), *m/z* calcd. for C_10_H_12_N_3_O_3_ [M + H]^+^ 222.0873; found 222.0875.

*2-(2-Azidoethoxy)-5-methylbenzaldehyde* (**1g**), Yield: 165 mg (80%). Yellow amorphous solid. ^1^H NMR (400 MHz, CDCl_3_) δ 10.50 (s, 1H), 7.68 (d, *J* = 2.0 Hz, 1H), 7.37 (dd, *J* = 8.4, 2.0 Hz, 1H), 6.89 (d, *J* = 8.4 Hz, 1H), 4.26 (t, *J* = 4.9 Hz, 2H), 3.68 (t, *J* = 4.9 Hz, 2H), 2.34 (s, 3H). ^13^C NMR (101 MHz, CDCl_3_) 189.6, 158.6, 136.5, 131.0, 128.6, 124.8, 112.4, 67.6, 50.3, 20.3. HRMS (ESI), *m/z* calcd. for C_10_H_12_N_3_O_2_ [M + H]^+^ 206.0924; found 206.0928. 

*2-(2-Azidoethoxy)-3-methoxybenzaldehyde* (**1h**), Yield: 186 mg (84%). Brown oil. ^1^H NMR (400 MHz, CDCl_3_) δ 10.51 (s, 1H), 7.45 (t, *J* = 4.8 Hz, 1H), 7.18 (d, *J* = 4.8 Hz, 2H), 4.33 (t, *J* = 5.0 Hz, 2H), 3.93 (s, 3H), 3.66 (t, *J* = 5.0 Hz, 2H). ^13^C NMR (101 MHz, CDCl_3_) δ 190.0, 152.7, 150.7, 129.9, 124.5, 119.4, 118.1, 72.4, 56.1, 51.1. HRMS (ESI), *m/z* calcd. for C_10_H_12_N_3_O_3_ [M + H]^+^ 222.0873; found 222.0877.

*2-(2-Azidoethoxy)-4-methoxybenzaldehyde* (**1i**), Yield: 195 mg (88%). Brown amorphous solid. ^1^H NMR (400 MHz, CDCl_3_) δ 10.35 (s, 1H), 7.86 (d, *J* = 8.7 Hz, 1H), 6.60 (dd, *J* = 8.7, 1.9 Hz, 2H), 6.44 (d, *J* = 1.9 Hz, 1H), 4.24 (t, *J* = 4.9 Hz, 2H), 3.89 (s, 3H), 3.69 (t, *J* = 4.9 Hz, 2H). ^13^C NMR (101 MHz, CDCl_3_) δ 187.9, 166.0, 162.1, 130.6, 119.2, 106.4, 98.8, 67.5, 55.7, 50.1. HRMS (ESI), *m/z* calcd. for C_10_H_12_N_3_O_3_ [M + H]^+^ 222.0873; found 222.0875.

*2-(2-Azidoethoxy)-5-methoxybenzaldehyde* (**1j**), Yield: 161 mg (73%). Yellow amorphous solid. 1H NMR (400 MHz, CDCl_3_) δ 10.48 (s, 1H), 7.36 (d, *J* = 3.2 Hz, 1H), 7.14 (dd, *J* = 9.0, 3.2 Hz, 1H), 6.95 (d, *J* = 9.0 Hz, 1H), 4.24 (t, *J* = 4.9 Hz, 2H), 3.82 (s, 3H), 3.67 (t, *J* = 4.9 Hz, 2H). ^13^C NMR (101 MHz, CDCl_3_) δ 189.2, 155.2, 154.2, 125.4, 123.3, 114.4, 110.6, 68.2, 55.8, 50.3. HRMS (ESI), m/z calcd. for C_10_H_12_N_3_O_3_ [M + H]+ 222.0873; found 222.0875.

*2-(2-Azidoethoxy)-4,6-dimethylbenzaldehyde* (**1k**), Yield: 167 mg (76%). Yellow amorphous solid. ^1^H NMR (400 MHz, CDCl_3_) δ 10.63 (s, 1H), 6.69 (s, 1H), 6.64 (s, 1H), 4.23 (t, *J* = 5.0 Hz, 2H), 3.67 (t, *J* = 5.0 Hz, 2H), 2.57 (s, 3H), 2.36 (s, 3H). ^13^C NMR (101 MHz, CDCl_3_) δ 191.4, 161.9, 145.6, 142.3, 125.8, 121.2, 110.6, 67.5, 50.3, 22.1, 21.5. HRMS (ESI), *m/z* calcd. for C_11_H_14_N_3_O_2_ [M + H]^+^ 220.1081; found 220.1083. 

*2-(2-Azidoethoxy)-1-naphthaldehyde* (**7**), Yield: 219 mg (99%). Reddish amorphous solid. ^1^H NMR (400 MHz, CDCl_3_) δ 10.95 (s, 1H), 9.30 (d, *J* = 8.4 Hz, 1H), 8.08 (d, *J* = 9.1 Hz, 1H), 7.81 (d, *J* = 8.4 Hz, 1H), 7.70–7.60 (m, 1H), 7.47 (t, *J* = 7.1 Hz, 1H), 7.28 (d, *J* = 7.5 Hz, 1H), 4.41 (t, *J* = 5.0 Hz, 2H), 3.75 (t, *J* = 5.0 Hz, 3H). ^13^C NMR (101 MHz, CDCl_3_) δ 191.7, 162.4, 137.5, 131.5, 130.0, 129.0, 128.3, 125.1, 125.1, 117.3, 113.3, 68.4, 50.4. HRMS (ESI), *m/z* calcd. for C_13_H_12_N_3_O_2_ [M + H]^+^ 242.0930; found 242.0926.

*1-(2-(2-Azidoethoxy)phenyl)ethan-1-one* (**8**), Yield: 128 mg (68%). Brown oil. ^1^H NMR (400 MHz, CDCl_3_) δ 7.75 (dd, *J* = 7.6, 1.6 Hz, 1H), 7.47 (td, *J* = 8.4, 1.6 Hz, 1H), 7.05 (t, *J* = 7.6 Hz, 1H), 6.95 (d, *J* = 8.4 Hz, 1H), 4.23 (t, *J* = 5.0 Hz, 2H), 3.72 (t, *J* = 5.0 Hz, 2H), 2.67 (s, 3H). ^13^C NMR (101 MHz, CDCl_3_) δ 199.6, 157.3, 133.5, 130.5, 128.8, 121.3, 112.4, 66.9, 50.4, 31.8. HRMS (ESI), *m/z* calcd. for C_10_H_11_N_3_NaO_2_ [M + Na]^+^ 228.0743; found 228.0746. 

#### 4.3.1. 2-((2-Azidoethyl)thio)benzaldehyde (**1l**)

Step 1. 2-((2-Hydroxyethyl)thio)benzaldehyde was obtained according to a slightly modified literature procedure [34]. 2-Mercaptoethanol (0.647 g, 8.3 mmol, 1 eq) was added to a solution of 2-fluorobenzaldehyde (1029 mg, 8.3 mmol, 1 eq) and K_2_CO_3_ (1.147 g, 8.3 mmol, 1 eq) in 5 mL of DMF and the mixture was stirred for 36 h at 60 °C. The reaction solution was diluted with water and extracted with chloroform. Combined organic layers were washed with water, brine and dried over anhydrous Na_2_SO_4_. Crude product was purified using column chromatography in a DCM–methanol system (0–40%) to give 2-((2-hydroxyethyl)thio)benzaldehyde (1.022 g, 68%) as a yellow oil. ^1^H NMR (400 MHz, CDCl_3_) δ 10.23 (s, 1H), 7.87 (dd, *J* = 7.6, 1.4 Hz, 1H), 7.63 (td, *J* = 7.6, 1.4 Hz, 1H), 7.56 (d, *J* = 7.6 Hz, 1H), 7.41–7.34 (t, *J* = 7.6 Hz, 1H), 5.00 (t, *J* = 6.0 Hz, 1H), 3.64 (q, *J* = 6.0 Hz, 2H), 3.10 (t, *J* = 6.0 Hz, 2H), in accordance with previously obtained literature data [34].

Step 2. 2-((2-Formylphenyl)thio)ethyl methanesulfonate was obtained according to a slightly modified mesilation literature procedure [35]. Mesyl chloride (214.2 mg, 1.87 mmol, 1.1 eq) was added to a solution of 2-((2-hydroxyethyl)thio)benzaldehyde and triethylamine (223.7 mg, 2.21 mmol, 1.3 equiv.) in DCM (6 mL) at 0 °C. One volume of saturated NaHCO_3_ solution was added and the mixture was stirred for 30 min. The organic layer was separated and the aqueous layer was extracted with DCM. Combined organic layers were washed with brine, dried over anhydrous Na_2_SO_4_ and the solvent was removed under reduced pressure to give 2-((2-formylphenyl)thio)ethyl methanesulfonate (237 mg, 91%) as a crude product that was used in the next stage directly without purification. 

Step 3. Sodium azide (151 mg, 2.33 mmol, 1.5 eq.) was added to a solution of 2-((2-formylphenyl)thio)ethyl methanesulfonate (404 mg, 1.55 mmol, 1.0 eq.) in DMF (4 mL). The mixture was stirred overnight at room temperature. The reaction solution was diluted with water and extracted with chloroform. Combined organic layers were washed with water, brine and dried over anhydrous Na_2_SO_4_. The solvent was removed under reduced pressure to give 2-((2-azidoethyl)thio)benzaldehyde **1l** (310 mg, 96%) as a yellow oil. ^1^H NMR (400 MHz, CDCl_3_) δ 10.43 (s, 1H), 7.88 (dd, *J* = 7.6, 1.1 Hz, 1H), 7.61–7.53 (m, 1H), 7.48 (d, *J* = 7.6 Hz, 1H), 7.38 (t, *J* = 7.6 Hz, 1H), 3.54 (t, *J* = 7.0 Hz, 1H), 3.16 (t, *J* = 7.0 Hz, 1H). ^13^C NMR (101 MHz, CDCl_3_) δ 191.3, 139.6, 134.8, 134.1, 132.1, 129.2, 126.5, 49.9, 32.9. HRMS (ESI), *m/z* calcd. for C_9_H_9_N_3_OSNa [M + Na]^+^ 230.0359; found 230.0362. 

#### 4.3.2. 2-((2-Azidoethyl)(methyl)amino)benzaldehyde (**1m**)

Step 1. 2-Azido-*N*-methylethan-1-amine (**9**) was obtained according to a slightly modified literature procedure [36]**.** Sodium azide (1.3 g, 20 mmol, 2.0 eq.) was added to a solution of *N*-methylamino-1-ethylbromide hydrobromide (2.19 g, 10 mmol, 1.0 eq.) in water (10 mL) and the mixture was stirred for 24 h at 80 °C. The mixture was cooled with an ice bath and NaOH (1.74 g) was added. The organic layer was separated and the aqueous layer was extracted with diethyl ether. Combined organic layers were washed with brine, dried over anhydrous Na_2_SO_4_ and the solvent was removed under reduced pressure to give 2-azido-*N*-methyl-ethanamine (0.72 g, 72%) as a colorless oil. ^1^H NMR (400 MHz, CDCl_3_) 3.43 (t, *J* = 5.7 Hz, 2H), 2.77 (t, *J* = 5.7 Hz, 2H), 2.46 (s, 3H), in accordance with previously obtained literature data [36].

Step 2. 2-Azido-*N*-methyl ethan-1-amine (266 mg, 2.66 mmol, 1.1 eq) was added to a solution of 2-fluorobenzaldehyde (300 mg, 2.42 mmol, 1 eq) and K_2_CO_3_ (367 mg, 2.66 mmol, 1.1 eq) in 2 mL of DMF and the mixture was stirred for 16 h at 110 °C. The reaction solution was diluted with water and extracted with chloroform. Combined organic layers were washed with water, brine and dried over anhydrous Na_2_SO_4_. The solvent was removed under reduced pressure to give 2-((2-azidoethyl)(methyl)amino)benzaldehyde **1m** (251 mg, 50%) as a brown oil. ^1^H NMR (400 MHz, CDCl_3_) δ 10.33 (s, 1H), 7.83 (dd, *J* = 7.7, 1.6 Hz, 1H), 7.56–7.50 (m, 1H), 7.19–7.10 (m, 2H), 3.49 (t, *J* = 6.0 Hz, 2H), 3.37 (t, *J* = 6.0 Hz, 2H), 2.96 (s, 3H). ^13^C NMR (101 MHz, CDCl_3_) δ 191.3, 154.8, 134.8, 130.6, 129.0, 122.5, 119.8, 56.2, 48.9, 43.5. HRMS (ESI), *m/z* calcd. for C_10_H_12_N_4_ONa [M + Na]^+^ 227.0903; found 227.0905. 

### 4.4. General Procedure for Synthesis of ***6a***–***6j***, ***6l***, ***6m*** and ***13*** (GP2)

Corresponding substituted 2-(2-azidoethoxy)benzaldehyde (0.315 mmol, 1.05 eq) and triphenylphosphine (82.6 mg, 0.315 mmol, 1.05 eq) were mixed in dry toluene (2 mL) in a sealed tube and heated at 110 °C for 1 h. The mixture was cooled down to room temperature. Homophthalic anhydride **5** (48.6 mg, 1 eq), 5-Br-substituted homophtalic anhydride [37] (72.3 mg, 1 eq—synthesis of compound **6j**) or *o*-phenylenediacetic acid anhydride [25] **12** (52.9 mg, 1 eq—synthesis of compound **13**) was added to the mixture and the stirring continued for 2 h, 12 h (**6j**) or 16 h (**13**) at room temperature or 110 °C (**13**). The precipitate was washed with toluene, then with hexane-ether (1:1) and dried at 80 °C. 

*(±)-(cis)-9-Oxo-6,7,14,14a-tetrahydro-9H-benzo [6,7][1,4]oxazepino [4,5b]isoquinoline-14-carboxylic acid* (**6a**), Yield: 82 mg (88%). White crystals. ^1^H NMR (400 MHz, DMSO-*d*_6_) δ 12.85 (s, 1H), 8.12–7.74 (m, 2H), 7.60 (t, *J* = 7.6 Hz, 1H), 7.44 (t, *J* = 7.6 Hz, 1H), 7.24 (t, *J* = 7.6 Hz, 1H), 7.14–7.06 (m, 1H), 7.04 (d, *J* = 7.9 Hz, 1H), 6.98 (t, *J* = 7.6 Hz, 1H), 5.40 (d, *J* = 4.4 Hz, 1H), 4.68–4.32 (m, 2H), 4.21 (d, *J* = 11.4 Hz, 1H), 3.79 (t, *J* = 10.9 Hz, 1H), 3.48 (ddd, *J* = 14.7, 10.9, 3.8 Hz, 1H). ^13^C NMR (101 MHz, DMSO-*d*_6_) δ 171.2, 162.8, 157.1, 136.6, 132.5, 132.0, 129.9, 129.4, 128.4, 128.4, 127.9, 127.3, 124.3, 122.8, 70.8, 59.7, 47.1, 44.4. HRMS (ESI), *m/z* calcd. for C_18_H_16_NO_4_ [M + H]^+^ 310.1074; found 310.1078. 

*(±)-(cis)-2-Fluoro-9-oxo-6,7,14,14a-tetrahydro-9H-benzo [6,7][1,4]oxazepino [4,5b]-isoquinoline-14-carboxylic acid* (**6b**), Yield: 77 mg (78%). White crystals. ^1^H NMR (400 MHz, DMSO-*d*_6_) δ 12.93 (s, 1H), 7.92 (d, *J* = 7.6 Hz, 1H), 7.81 (s, 1H), 7.61 (t, *J* = 7.9 Hz, 1H), 7.45 (t, *J* = 7.6 Hz, 1H), 7.15–7.02 (m, 2H), 6.93 (s, 1H), 5.39 (d, *J* = 4.5 Hz, 1H), 4.49 (d, *J* = 14.2 Hz, 2H), 4.16 (d, *J* = 11.4 Hz, 1H), 3.80 (t, *J* = 11.3 Hz, 1H), 3.46 (t, *J* = 11.3 Hz, 1H). ^13^C NMR (101 MHz, DMSO-*d*_6_) δ 171.0, 162.8, 158.3 (d, ^1^*J*_C-F_ = 240.6 Hz), 153.2, 136.4, 133.8, 132.6, 129.3, 128.5, 128.1, 127.3, 124.3 (d, ^3^*J*_C-F_ = 8.5 Hz), 116.1 (d, ^2^*J*_C-F_ = 23.0 Hz), 115.3 (d, ^2^*J*_C-F_ = 24.1 Hz), 70.8, 59.6, 47.4, 44.1. ^19^F NMR (376.5 MHz, CDCl_3_) δ -118.90. HRMS (ESI), *m/z* calcd. for C_18_H_15_NO_4_F [M + H]^+^ 328.0980; found 328.0983.

*(±)-(cis)-2-Chloro-9-oxo-6,7,14,14a-tetrahydro-9H-benzo [6,7][1,4]oxazepino [4,5b]-isoquinoline-14-carboxylic acid* (**6c**), Yield: 86 mg (83%). White crystals. ^1^H NMR (400 MHz, DMSO-*d*_6_) δ 12.86 (s, 1H), 7.92 (d, *J* = 7.6 Hz, 1H), 7.84–7.67 (m, 1H), 7.61 (td, *J* = 7.5, 1.5 Hz, 1H), 7.46 (t, *J* = 7.6 Hz, 1H), 7.32 (dd, *J* = 8.5, 2.6 Hz, 1H), 7.23–7.15 (m, 1H), 7.06 (d, *J* = 8.5 Hz, 1H), 5.39 (d, *J* = 4.4 Hz, 1H), 4.58–4.31 (m, 2H), 4.16 (d, *J* = 11.2 Hz, 1H), 3.84 (t, *J* = 9.7 Hz, 1H), 3.44 (ddd, *J* = 15.1, 11.2, 4.0 Hz, 1H). ^13^C NMR (101 MHz, DMSO-*d*_6_) δ 171.1, 162.8, 155.5, 136.4, 133.9, 132.6, 129.7, 129.4, 128.5, 128.4, 128.2, 128.1, 127.4, 124.7, 70.8, 59.8, 47.8, 43.5. HRMS (ESI), *m/z* calcd. for C_18_H_15_NO_4_Cl [M + H]^+^ 344.0684; found 344.0689.

*(±)-(cis)-2-Bromo-9-oxo-6,7,14,14a-tetrahydro-9H-benzo [6,7][1,4]oxazepino [4,5b]-isoquinoline-14-carboxylic acid* (**6d**),Yield: 93 mg (80%). White crystals. ^1^H NMR (400 MHz, DMSO-*d*_6_) δ 12.84 (s, 1H), 7.91 (d, *J* = 7.4 Hz, 1H), 7.76 (s, 1H), 7.61 (td, *J* = 7.6, 1.5 Hz, 1H), 7.51–7.40 (m, 2H), 7.33 (s, 1H), 7.00 (d, *J* = 8.4 Hz, 1H), 5.38 (d, *J* = 4.4 Hz, 1H), 4.56–4.31 (m, 2H), 4.15 (d, *J* = 11.0 Hz, 1H), 3.85 (t, *J* = 10.8 Hz, 1H), 3.44 (ddd, *J* = 15.1, 11.3, 3.9 Hz, 1H). ^13^C NMR (101 MHz, DMSO-*d*_6_) δ 171.0, 162.8, 155.9, 136.5, 134.3, 132.7, 132.5, 131.4, 129.4, 128.4, 128.2, 127.4, 125.1, 116.1, 70.7, 59.7, 47.8, 43.6. HRMS (ESI), *m/z* calcd. for C_18_H_15_NO_4_Br [M + H]^+^ 388.0179; found 388.0182.

*(±)-(cis)-2-Nitro-9-oxo-6,7,14,14a-tetrahydro-9H-benzo [6,7][1,4]oxazepino [4,5b]-isoquinoline-14-carboxylic acid* (**6e**)**,** Yield: 78 mg (73%). White crystals. ^1^H NMR (400 MHz, DMSO-*d*_6_) δ 12.96 (s, 1H), 8.23–8.08 (m, 2H), 7.91 (d, *J* = 7.6 Hz, 1H), 7.76 (s, 1H), 7.62 (t, *J* = 7.4 Hz, 1H), 7.46 (t, *J* = 7.5 Hz, 1H), 7.27 (d, *J* = 8.7 Hz, 1H), 5.55 (d, *J* = 4.4 Hz, 1H), 4.64–4.41 (m, 2H), 4.31 (d, *J* = 11.3 Hz, 1H), 4.09–3.91 (m, 1H), 3.50 (ddd, *J* = 14.8, 11.0, 3.8 Hz, 1H). ^13^C NMR (101 MHz, DMSO-*d*_6_) δ 171.0, 162.8, 162.4, 143.6, 136.3, 132.8, 132.7, 129.3, 128.4, 128.3, 127.4, 125.7, 124.7, 124.3, 71.4, 59.7, 48.0, 43.2. HRMS (ESI), *m/z* calcd. for C_18_H_15_N_2_O_6_ [M + H]^+^ 355.0925; found 355.0928.

*(±)-(cis)-4-Methoxy-9-oxo-6,7,14,14a-tetrahydro-9H-benzo [6,7][1,4]oxazepino [4,5b]-isoquinoline-14-carboxylic acid* (**6f**), Yield: 84 mg (83%). White crystals. ^1^H NMR (400 MHz, DMSO-*d*_6_) δ 12.80 (s, 1H), 8.03–7.79 (m, 2H), 7.59 (t, *J* = 7.1 Hz, 1H), 7.43 (t, *J* = 7.5 Hz, 1H), 7.02–6.85 (m, 2H), 6.64 (s, 1H), 5.39 (d, *J* = 4.3 Hz, 1H), 4.62–4.32 (m, 2H), 4.19 (d, *J* = 8.5 Hz, 1H), 3.76 (s, 3H), 3.70 (s, 1H), 3.49 (t, *J* = 10.9 Hz, 1MH). ^13^C NMR (101 MHz, DMSO-*d*_6_) δ 171.1, 162.8, 152.8, 145.9, 136.7, 133.4, 132.5, 129.4, 128.4, 127.9, 127.3, 124.3, 119.8, 113.2, 70.1, 59.7, 56.2, 47.2, 44.4. HRMS (ESI), *m/z* calcd. for C_19_H_17_NNaO_5_ [M + Na]^+^ 362.0999; found 362.1000.

*(±)-(cis)-3-Methoxy-9-oxo-6,7,14,14a-tetrahydro-9H-benzo [6,7][1,4]oxazepino [4,5b]-isoquinoline-14-carboxylic acid* (**6g**), Yield: 82 mg (80%). White crystals. ^1^H NMR (400 MHz, DMSO-*d*_6_) δ 12.81 (s, 1H), 8.04–7.74 (m, 2H), 7.59 (t, *J* = 7.5 Hz, 1H), 7.43 (t, *J* = 7.5 Hz, 1H), 6.97 (s, 1H), 6.60 (d, *J* = 2.5 Hz, 1H), 6.57 (d, *J* = 8.0 Hz, 1H), 5.32 (d, *J* = 4.4 Hz, 1H), 4.64–4.32 (m, 2H), 4.20 (d, *J* = 9.6 Hz, 1H), 3.78 (t, *J* = 10.0 Hz, 1H), 3.70 (s, 3H), 3.53–3.41 (m, 1H). ^13^C NMR (101 MHz, DMSO-*d*_6_) δ 171.2, 162.8, 160.4, 158.1, 136.7, 132.4, 129.4, 129.0, 128.4, 127.9, 127.3, 123.9, 109.5, 108.6, 71.0, 59.2, 55.7, 47.3, 44.3. HRMS (ESI), *m/z* calcd. for C_19_H_18_NO_5_ [M + H]^+^ 340.1180; found 340.1181.

*(±)-(cis)-2-Methoxy-9-oxo-6,7,14,14a-tetrahydro-9H-benzo [6,7][1,4]oxazepino [4,5b]-isoquinoline-14-carboxylic acid* (**6h**), Yield: 85 mg (84%). White crystals. ^1^H NMR (400 MHz, DMSO-*d*_6_) δ 12.88 (s, 1H), 8.04–7.78 (m, 1H), 7.61 (t, *J* = 7.1 Hz, 2H), 7.44 (t, *J* = 7.5 Hz, 1H), 6.96 (d, *J* = 8.7 Hz, 1H), 6.78 (dd, *J* = 8.6, 2.5 Hz, 2H), 6.65 (s, 1H), 5.35 (d, *J* = 4.3 Hz, 1H), 4.67–4.34 (m, 1H), 4.16 (d, *J* = 10.1 Hz, 1H), 3.71 (t, *J* = 9.9 Hz, 1H), 3.58 (s, 3H), 3.51–3.42 (m, 1H). ^13^C NMR (101 MHz, DMSO-*d*_6_) δ 171.2, 162.8, 155.5, 150.6, 136.6, 132.9, 132.5, 129.4, 128.5, 127.9, 127.3, 123.2, 114.5, 113.8, 71.0, 59.7, 55.6, 47.0, 44.4. HRMS (ESI), *m/z* calcd. for C_19_H_18_NO_5_ [M + H]^+^ 340.1180; found 340.1179.

*(±)-(cis)-2-Methyl-9-oxo-6,7,14,14a-tetrahydro-9H-benzo [6,7][1,4]oxazepino-[4,5b]-isoquinoline-14-carboxylic acid* (**6i**), Yield: 71 mg (73%). White crystals. ^1^H NMR (400 MHz, DMSO-*d*_6_) δ 12.75 (s, 1H), 7.91 (d, *J* = 7.6 Hz, 1H), 7.87–7.70 (m, 1H), 7.59 (t, *J* = 7.6 Hz, 1H), 7.44 (t, *J* = 7.6 Hz, 1H), 7.06 (dd, *J* = 7.8, 2.1 Hz, 1H), 6.96 (s, 1H), 6.91 (d, *J* = 8.0 Hz, 1H), 5.33 (d, *J* = 4.3 Hz, 1H), 4.59–4.25 (m, 2H), 4.10 (d, *J* = 10.5 Hz, 1H), 3.80 (t, *J* = 11.3 Hz, 1H), 3.42 (ddd, *J* = 14.7, 10.9, 4.0 Hz, 1H), 2.15 (s, 3H). ^13^C NMR (101 MHz, DMSO-*d*_6_) δ 171.2, 162.8, 154.3, 136.9, 133.2, 132.4, 131.4, 130.3, 129.5, 129.1, 128.4, 128.0, 127.3, 122.5, 70.5, 60.2, 47.7, 43.5, 20.9. HRMS (ESI), *m/z* calcd. for C_19_H_17_NNaO_4_ [M + Na]^+^ 346.1055; found 346.1051.

*(±)-(cis)-11-Bromo-9-oxo-6,7,14,14a-tetrahydro-9H-benzo [6,7][1,4]oxazepino [4,5b]-isoquinoline-14-carboxylic acid* (**6j**)**,** Yield: 78 mg (67%). White crystals. ^1^H NMR (400 MHz, DMSO-*d*_6_) δ 12.97 (s, 1H), 8.21–7.86 (m, 2H), 7.81 (d, *J* = 7.4 Hz, 1H), 7.35–7.20 (m, 1H), 7.12–6.92 (m, 3H), 5.42 (d, *J* = 3.2 Hz, 1H), 4.76–4.34 (m, 2H), 4.22 (d, *J* = 9.7 Hz, 1H), 3.77 (t, *J* = 11.0 Hz, 1H), 3.49 (t, *J* = 12.3 Hz, 1H). ^13^C NMR (101 MHz, CDCl_3_) δ 170.8, 161.5, 157.2, 135.0, 135.2, 131.8, 131.4, 130.8, 130.0, 129.9, 128.2, 124.3, 122.8, 121.0, 70.8, 59.4, 46.5, 44.6. HRMS (ESI), *m/z* calcd. for C_18_H_15_NO_4_Br [M + H]^+^ 388.0179; found 388.0182.

*(±)-(cis)-1,3-Dimethyl-9-oxo-6,7,14,14a-tetrahydro-9H-benzo [6,7][1,4]oxazepino [4,5b]-isoquinoline-14-carboxylic acid* (**6l**), Yield: 72 mg (71%). White crystals. ^1^H NMR (400 MHz, DMSO-*d*_6_) δ 12.88 (s, 1H), 7.96 (d, *J* = 7.5 Hz, 1H), 7.57 (td, *J* = 7.5, 1.6 Hz, 1H), 7.48 (t, *J* = 7.5 Hz, 1H), 7.13 (d, *J* = 7.6 Hz, 1H), 6.87 (s, 1H), 6.78 (s, 1H), 5.33 (d, *J* = 11.4 Hz, 1H), 4.57–4.47 (m, 2H), 4.06–3.96 (m, 1H), 3.70 (dd, *J* = 10.6, 4.8 Hz, 1H), 2.92 (ddd, *J* = 14.6, 12.1, 4.9 Hz, 1H), 2.32 (s, 3H), 2.27 (s, 3H). ^13^C NMR (101 MHz, DMSO-*d*_6_) δ 172.5, 162.6, 155.1, 140.2, 138.2, 137.7, 132.6, 129.5, 128.7, 128.5, 128.2, 126.0, 125.7, 121.4, 69.8, 57.7, 51.3, 39.7, 21.0, 20.2. HRMS (ESI), *m/z* calcd. for C_20_H_20_NNaO_4_ [M + Na]^+^ 360.1212; found 360.1213.

*(±)-(trans)-11-Oxo-8,9,16,16a-tetrahydro-11H-naphtho [1′,2′:6,7][1,4]oxazepino [4,5b]-isoquinoline-16-carboxylic acid* (**6m**), Yield: 102 mg (95%). White crystals. ^1^H NMR (400 MHz, DMSO-*d*_6_) δ 12.80 (s, 1H), 8.21 (d, *J* = 8.4 Hz, 1H), 8.10–7.97 (m, 2H), 7.94 (d, *J* = 7.7 Hz, 1H), 7.65–7.40 (m, 4H), 7.29 (d, *J* = 8.7 Hz, 1H), 7.13 (d, *J* = 7.4 Hz, 1H), 6.17 (d, *J* = 9.8 Hz, 1H), 4.73–4.46 (m, 2H), 4.21 (td, *J* = 10.8, 5.6 Hz, 1H), 3.87 (dd, *J* = 10.3, 5.1 Hz, 1H), 3.14–2.94 (m, 1H). ^13^C NMR (101 MHz, DMSO-*d*_6_) δ 172.3, 163.1, 153.4, 137.0, 132.6, 131.9, 131.8, 131.8, 129.3, 128.9, 128.4, 128.2, 126.9, 126.8, 125.3, 124.9, 123.9, 123.2, 69.3, 57.6, 51.2, 40.6. HRMS (ESI), *m/z* calcd. for C_22_H_17_NO_4_Na [M + Na]^+^ 382.1050; found 382.1042.

*(±)-(trans)-9-Oxo-6,7,9,10,15,15a-hexahydrobenzo[f]benzo [4,5]azepino [1,2d][1,4]- oxazepine-15-carboxylic acid* (**13**), Yield: 48 mg (52%). White crystals. ^1^H NMR (400 MHz, DMSO-*d*_6_) δ 12.56 (s, 1H), 7.46–7.35 (m, 2H), 7.34–7.20 (m, 4H), 7.16 (t, *J* = 7.1 Hz, 1H), 7.10 (d, *J* = 7.9 Hz, 1H), 5.85 (d, *J* = 11.6 Hz, 1H), 4.74 (d, *J* = 14.1 Hz, 1H), 4.48 (d, *J* = 11.5 Hz, 1H), 4.41 (d, *J* = 15.3 Hz, 1H), 4.07–3.99 (m, 1H), 3.94 (td, *J* = 11.6, 3.1 Hz, 1H), 3.36 (d, *J* = 14.2 Hz, 1H), 2.82 (ddd, *J* = 15.5, 12.0, 3.6 Hz, 1H). ^13^C NMR (101 MHz, DMSO-*d*_6_) δ 173.3, 171.9, 154.3, 134.7, 133.3, 131.3, 131.2, 131.1, 130.6, 129.7, 127.9, 127.9, 124.6, 122.8, 71.0, 61.4, 55.5, 42.5, 39.3. HRMS (ESI), *m/z* calcd. for C_19_H_17_NNaO_4_ [M + Na]^+^ 346.1050; found 346.1046.

#### 1-Methoxy-9-oxo-6,7,14,14a-tetrahydro-9H-benzo [6,7][1,4]oxazepino [4,5b]isoquinoline-14-carboxylic acid (**6k**)

This compound was obtained using a modified general procedure GP2. Precipitate did not appear at the final stage, so extraction with NaHCO_3_ was performed: the solvent was evaporated and the residue was diluted with DCM, and the resulting mixture was extracted with a saturated solution of NaHCO_3_. Combined aqueous phases were acidified with HCl (40%) to a pH lower than 7 (controlled by indicator litmus paper)—acid precipitated. The resulting suspension was extracted with DCM. Combined organic layers were washed with water, brine, dried over anhydrous Na_2_SO_4_ and concentrated in vacuo to give compound **6k** (94 mg, 92%, *dr* = 5:1 *trans:cis*) as a white solid. 

^1^H NMR (400 MHz, DMSO-*d*_6_) showed major product signals δ 12.75 (s, 1H), 7.86 (d, *J* = 7.7 Hz, 1H), 7.54 (t, *J* = 7.7 Hz, 1H), 7.39 (t, *J* = 7.5 Hz, 1H), 7.37–7.32 (m, 1H), 7.21 (t, *J* = 8.2 Hz, 1H), 6.75 (d, *J* = 8.4 Hz, 1H), 6.64 (d, *J* = 7.8 Hz, 1H), 5.63 (d, *J* = 5.1 Hz, 1H), 4.64–4.53 (m, 2H), 4.17–4.07 (m, 1H), 3.95 (td, *J* = 10.9, 4.6 Hz, 1H), 3.58 (s, 3H), 3.19 (ddd, *J* = 14.7, 10.3, 4.8 Hz, 1H), and minor product observed signals δ 7.97 (d, *J* = 7.6 Hz, 1H), 6.93 (d, *J* = 8.4 Hz, 1H), 4.51 (d, *J* = 4.7 Hz, 1H), 3.83 (s, 3H), 3.80 (t, *J* = 3.8 Hz, 1H). ^13^C NMR (101 MHz, DMSO-*d*_6_) showed major product signals δ 172.7, 162.9, 158.9, 157.4, 137.4, 132.2, 130.2, 128.8, 128.0, 127.7, 120.1, 115.2, 108.8, 70.7, 57.0, 56.0, 48.6, 44.4, and minor product observed signals δ 171.1, 157.3, 132.1, 130.4, 130.2, 128.2, 127.6, 127.5, 115.6, 108.1, 69.1, 56.6, 56.0, 48.4. HRMS (ESI), *m/z* calcd. for C_19_H_18_NO_5_ [M + H]^+^ 340.1180; found 340.1181.

### 4.5. Procedure for Synthesis of ***10b*** and ***10e***

Corresponding azide (0.315 mmol, 1.05 eq) and triphenylphosphine (82.6 mg, 0.315 mmol, 1.05 eq) were mixed in a sealed tube in dry toluene (2 mL) and heated to 110 °C for 1 h. The mixture was cooled down to room temperature. Homophthalic anhydride **5** (48.6 mg, 1 eq) was added to the mixture and it was stirred for 2 h (synthesis of compound **10b**) or 72 h (synthesis of compound **10e**) at room temperature. The precipitate was washed with toluene, hexane-ether (1:1) and dried at 80 °C. The precipitate was diluted with DSMO. K_2_CO_3_ (1.1 eq.) was added to the mixture and it was heated at 150 °C for 2 h. The mixture was diluted with water and extracted with DCM, and combined organic fractions were washed with a saturated solution of NaHCO_3_, water and brine, then dried over anhydrous Na_2_SO_4_ concentrated in vacuo. Crude products were purified using column chromatography in a petroleum ether–ethyl acetate system (gradient elution 5–25%).

*6,7,14,14a-Tetrahydro-9H-benzo [6,7][1,4]thiazepino [4,5b]isoquinolin-9-one* (**10b**), Yield: 50 mg (59%). White crystals. ^1^H NMR (400 MHz, CDCl_3_) δ 8.03 (d, *J* = 7.4 Hz, 1H), 7.61 (d, *J* = 7.5 Hz, 1H), 7.53 (t, *J* = 7.4 Hz, 1H), 7.42–7.32 (m, 2H), 7.15 (td, *J* = 8.8, 7.3, 1.8 Hz, 1H), 7.12–7.02 (m, 2H), 5.37–5.29 (m, 1H), 5.07 (d, *J* = 13.7 Hz, 1H), 3.64 (dd, *J* = 16.3, 5.5 Hz, 1H), 3.50 (dd, *J* = 16.3, 3.6 Hz, 1H), 3.42 (ddd, *J* = 13.9, 8.5, 5.6 Hz, 1H), 2.93 (dd, *J* = 8.4, 3.0 Hz, 2H). ^13^C NMR (101 MHz, CDCl_3_) δ 163.3, 144.2, 137.5, 135.2, 134.4, 132.4, 129.1, 128.7, 128.2, 127.9, 127.6, 127.2, 127.0, 58.9, 48.1, 33.0, 32.4. HRMS (ESI), *m/z* calcd. for C_17_H_16_NOS [M + H]^+^ 282.0947; found 282.0949.

*5-Methyl-6,7,14,14a-tetrahydrobenzo [5,6][1,4]diazepino [1,7b]isoquinolin-9(5H)-one* (**10e**), Yield: 38 mg (46%). White crystals. ^1^H NMR (400 MHz, CDCl_3_) δ 8.09 (d, *J* = 7.5 Hz, 1H), 7.47 (td, *J* = 7.4, 1.1 Hz, 1H), 7.38 (t, *J* = 7.5 Hz, 1H), 7.35–7.30 (m, 1H), 7.29–7.25 (m, 1H), 7.17 (d, *J* = 7.1 Hz, 1H), 7.05 (d, *J* = 7.9 Hz, 1H), 6.97 (t, *J* = 7.4 Hz, 1H), 5.01 (dd, *J* = 9.7, 4.4 Hz, 1H), 4.24 (dt, *J* = 13.9, 4.3 Hz, 1H), 3.69–3.51 (m, 2H), 3.14 (dd, *J* = 15.6, 4.2 Hz, 1H), 3.10–2.96 (m, 2H), 2.89 (s, 3H). ^13^C NMR (101 MHz, CDCl_3_) δ 163.4, 149.8, 138.0, 132.3, 131.8, 129.4, 129.1, 128.4, 127.0, 127.0, 126.9, 122.2, 118.2, 58.4, 56.0, 42.5, 41.7, 33.2. HRMS (ESI), *m/z* calcd. for C_18_H_19_N_2_O [M + H]^+^ 279.1492; found 279.1493.

### 4.6. Procedure for Synthesis of ***10a***, ***10c*** and ***10d***

Corresponding azide (0.315 mmol, 1.05 eq) and triphenylphosphine (82.6 mg, 0.315 mmol, 1.05 eq) were mixed in a sealed tube in dry toluene (2 mL) and heated to 110 °C for 1 h. The mixture was cooled down to room temperature. Homophthalic anhydride **5** (48.6 mg, 1 eq) or 5-NO_2_-substituted homophtalic anhydride [38] (62 mg, 1 eq; synthesis of compound **10c**) was added to the mixture and it was stirred for 2 h or 12 h (synthesis of compound **10c**) at room temperature. The solvent was evaporated and the residue was diluted with DCM, and the resulting mixture was extracted with a saturated solution of NaHCO_3_. Combined aqueous fractions were acidified with HCl (40%) to a pH lower than 7 (controlled by paper indicator)—acid precipitated. The resulting suspension was extracted with DCM. Combined organic layers were washed with water, brine, dried over anhydrous Na_2_SO_4_ and concentrated in vacuo. Crude product was diluted with DMSO. K_2_CO_3_ (1 eq.) was added to the mixture and it was heated to 100 °C/150 °C for 1–15 h (**10d**—100 °C, 15 h; **10a**—150 °C, 1 h; **10c**—100 °C, 1 h). The mixture was diluted with water and extracted with DCM, and combined organic fractions were washed with a saturated solution of NaHCO_3_, water and brine, then dried over anhydrous Na_2_SO_4_ and concentrated in vacuo. Products **10a** and **10c** were pure. Crude product **10d** was purified using column chromatography in a petroleum ether–ethyl acetate system (gradient elution 5–25%).

*14a-Methyl-6,7,14,14a-tetrahydro-9H-benzo [6,7][1,4]oxazepino [4,5b]isoquinolin-9-one* (**10a**), Yield: 72 mg (86%). White crystals. ^1^H NMR (400 MHz, CDCl_3_) δ 8.04 (d, *J* = 7.7 Hz, 1H), 7.48 (t, *J* = 7.1 Hz, 1H), 7.34 (t, *J* = 7.5 Hz, 1H), 7.27 (d, *J* = 7.6 Hz, 1H), 7.24–7.17 (m, 1H), 7.17–7.10 (m, 1H), 7.09–7.03 (m, 1H), 6.99 (t, *J* = 7.5 Hz, 1H), 4.78–4.65 (m, 1H), 4.17 (dd, *J* = 11.4, 3.2 Hz, 1H), 4.07 (td, *J* = 11.4, 3.4 Hz, 1H), 3.69–3.52 (m, 2H), 3.13 (d, *J* = 16.1 Hz, 1H), 1.81 (s, 3H). ^13^C NMR (101 MHz, CDCl_3_) δ 163.9, 156.4, 137.1, 136.6, 132.2, 129.1, 128.7, 128.5, 127.1, 127.0, 126.4, 124.2, 123.6, 70.2, 61.6, 41.5, 41.3, 26.1. HRMS (ESI), *m/z* calcd. for C_18_H_18_NO_2_ [M + H]^+^ 280.1332; found 280.1335.

*11-Nitro-6,7,14,14a-tetrahydro-9H-benzo [6,7][1,4]oxazepino [4,5b]isoquinolin-9-one* (**10c**), Yield: 46 mg (50%). Yellow amorphous solid. ^1^H NMR (400 MHz, CDCl_3_) δ 8.86 (d, *J* = 2.3 Hz, 1H), 8.37 (dd, *J* = 8.3, 2.4 Hz, 1H), 7.60 (d, *J* = 8.3 Hz, 1H), 7.27–7.18 (m, 1H), 7.14–7.06 (m, 1H), 6.98–6.86 (m, 2H), 5.20–5.11 (m, 1H), 4.87 (dt, *J* = 14.1, 1.9 Hz, 1H), 4.46–4.36 (m, 1H), 3.89 (td, *J* = 11.8, 2.4 Hz, 1H), 3.73–3.46 (m, 3H). ^13^C NMR (101 MHz, CDCl_3_) δ 161.1, 158.4, 147.6, 144.0, 132.7, 130.3, 129.7, 128.5, 126.7, 126.6, 124.2, 124.0, 122.6, 71.4, 56.4, 47.4, 31.5. HRMS (ESI), *m/z* calcd. for C_17_H_15_N_2_O_4_ [M + H]^+^ 311.1026; found 311.1029.

*1-Methoxy-6,7,14,14a-tetrahydro-9H-benzo [6,7][1,4]oxazepino [4,5b]isoquinolin-9-one* (**10d**), Yield: 46 mg (52%). White crystals. ^1^H NMR (400 MHz, CDCl_3_) δ 8.13 (d, *J* = 7.4 Hz, 1H), 7.49–7.43 (m, 1H), 7.39 (t, *J* = 7.5 Hz, 1H), 7.32–7.25 (m, 3H), 7.21 (d, *J* = 7.4 Hz, 1H), 6.81–6.71 (m, 2H), 5.48 (dd, *J* = 13.4, 3.5 Hz, 1H), 4.76 (dd, *J* = 14.4, 5.1 Hz, 1H), 4.33 (ddd, *J* = 12.3, 10.6, 5.2 Hz, 1H), 3.91–3.82 (m, 4H), 3.41 (t, *J* = 14.4 Hz, 1H), 3.24 (ddd, *J* = 14.3, 12.5, 5.2 Hz, 1H), 2.87 (dd, *J* = 15.6, 3.4 Hz, 1H). ^13^C NMR (101 MHz, CDCl_3_) δ 163.9, 156.8, 154.7, 138.1, 131.6, 129.6, 129.5, 128.2, 127.0, 126.9, 121.5, 115.8, 107.4, 69.0, 55.8, 53.8, 40.2, 34.3. HRMS (ESI), *m/z* calcd. for C_18_H_18_NO_3_ [M + H]^+^ 296.1281; found 296.1286.

### 4.7. (±)-(trans)-4-Oxo-1,3,4,6,7,12b-hexahydrobenzo[f][1,4]thiazino [4,3d][1,4]oxazepine-1-carboxylic acid (***16***)

Azide **1a** (60.1 mg, 0.315 mmol, 1.05 eq) and triphenylphosphine (82.6 mg, 0.315 mmol, 1.05 eq) were mixed in a sealed tube in dry toluene (2 mL) and heated to 110 °C for 1 h. The mixture was cooled down to room temperature. Thiodiglycolic acid **14** (45 mg, 0.3 mmol, 1 eq) and acetic anhydride (0.345 mmol, 1.15 eq) were added to the mixture and it was stirred for 24 h at 110 °C. The precipitate was washed with toluene, hexane-ether (1:1) and dried at 80 °C to give compound **16** (58 mg, 66%) as brownish crystals. ^1^H NMR (400 MHz, DMSO-*d*_6_) δ 12.93 (s, 1H), 7.44–7.32 (m, 2H), 7.18 (td, *J* = 7.4, 0.8 Hz, 1H), 7.10 (d, *J* = 7.9 Hz, 1H), 5.22 (d, *J* = 8.5 Hz, 1H), 4.49–4.36 (m, 1H), 4.17 (d, *J* = 8.5 Hz, 1H), 4.04 (dd, *J* = 11.2, 3.0 Hz, 1H), 3.97 (td, *J* = 11.3, 3.7 Hz, 1H), 3.70 (d, *J* = 14.5 Hz, 1H), 3.08 (d, *J* = 14.6 Hz, 1H), 2.93 (ddd, *J* = 15.3, 11.4, 4.3 Hz, 1H). ^13^C NMR(101 MHz, DMSO-*d*_6_) δ 172.2, 168.2, 155.3, 131.2, 130.1, 129.9, 124.9, 123.0, 71.4, 60.2, 45.3, 41.3, 28.8. HRMS (ESI), *m/z* calcd. for C_13_H_14_NO_4_S [M + H]^+^ 280.0638; found 280.0640.

### 4.8. 11-(4-Methoxyphenyl)-6,7,10,12a-tetrahydro-9H-benzo[f]pyrido [1,2d][1,4]oxazepin-9-one (***17***)

Azide **1a** (100.4 mg, 0.525 mmol, 1.05 eq), triphenylphosphine (137.7 mg, 0.525 mmol, 1.05 eq) and *p*-methoxyphenyl-substituted glutaconic acid **15** (109.1 mg, 0.5 mmol, 1 eq) were mixed in dry toluene (10 mL) in a round-bottom flask equipped with a Dean-Star trap and heated to reflux for 20 h. The solvent of the resulting mixture was evaporated, and the crude product was purified using column chromatography in a hexane–ethyl acetate system (5–40%) to give compound **17** (82 mg, 51%) as white crystals. ^1^H NMR (400 MHz, CDCl_3_) δ 7.48 (d, *J* = 8.8 Hz, 2H), 7.28 (td, *J* = 8.9, 7.6, 1.3 Hz, 1H), 7.23 (d, *J* = 7.6 Hz, 1H), 7.13 (d, *J* = 7.2 Hz, 1H), 7.08–7.02 (m, 1H), 6.98 (d, *J* = 8.8 Hz, 2H), 6.34 (dd, *J* = 5.7, 2.9 Hz, 1H), 5.50 (d, *J* = 4.0 Hz, 1H), 4.90 (dt, *J* = 14.3 Hz, 1.8, 1H), 4.50 (dt, *J* = 12.5, 2.3 Hz, 1H), 3.87 (s, 3H), 3.84–3.74 (m, 1H), 3.53–3.34 (m, 2H), 3.27 (dt, *J* = 20.2, 2.6 Hz, 1H). ^13^C NMR (101 MHz, CDCl_3_) δ 167.6, 159.8, 159.5, 136.0, 132.9, 130.5, 129.6, 127.1, 126.4, 124.1, 122.1, 117.8, 114.2, 72.4, 58.4, 55.4, 47.6, 35.1. HRMS (ESI), *m/z* calcd. for C_20_H_20_NO_3_ [M + H]^+^ 322.1438; found 322.1437.

### 4.9. (±)-(trans)-9-Oxo-6,7,14,14a-tetrahydro-9H-benzo [6,7][1,4]oxazepino [4,5b]isoquinoline-14-carboxylic acid (***6a’***)

Compound **6a** (100 mg, 0.323 mmol) was dissolved in DMF heated to 110 °C for 4 h. The solvent of the resulting mixture was evaporated, and the residue was washed with water and dried at 80 °C to give compound **6a’** (84 mg, 84%) as white crystals. ^1^H NMR (400 MHz, DMSO-*d*_6_) δ 13.08 (s, 1H), 7.78 (d, *J* = 7.5 Hz, 1H), 7.70 (d, *J* = 7.4 Hz, 1H), 7.62 (t, *J* = 7.0 Hz, 1H), 7.39 (t, *J* = 7.2 Hz, 1H), 7.22–7.11 (m, 1H), 7.03 (d, *J* = 7.7 Hz, 1H), 6.96–6.82 (m, 2H), 5.50 (s, 1H), 4.80–4.66 (m, 2H), 4.50 (d, *J* = 12.5 Hz, 1H), 3.71–3.57 (m, 1H), 3.38–3.30 (m, 1H). ^13^C NMR (101 MHz, DMSO-*d*_6_) δ 172.5, 161.9, 159.5, 136.0, 133.2, 132.8, 130.3, 129.5, 128.3, 128.2, 127.8, 127.4, 123.9, 122.2, 71.7, 57.9, 48.4, 45.3. HRMS (ESI), *m/z* calcd. for C_18_H_16_NO_4_ [M + H]^+^ 310.1074; found 310.1075.

## Data Availability

Data are available from the corresponding authors upon reasonable request.

## Supplementary Materials

The following supporting information can be downloaded at: https://www.mdpi.com/article/10.3390/molecules27238130/s1. Copies of NMR spectra. X-ray data [39,40,41]. Crystallographic data: CCDC 2207932 (**6a**), 2207933 (**6c**), 2207934 (**6d**), 2207935 (**6e**), 2207937 (**6f**), 2178410 (**6m**), 2207930 (**13**), 2207931 (**16**), 2207936 (**6a’**) contain the supplementary crystallographic data for this paper. These data can be obtained free of charge from The Cambridge Crystallographic Data Centre via www.ccdc.cam.ac.uk/structures (accessed on 17 September 2022).

## Sample Availability

Samples of the compounds **6** and **10** are available from the authors upon reasonable request.
