# Supplementary material for: One-Pot Sequence of Staudinger/aza-Wittig/Castagnoli–Cushman Reactions Provides Facile Access to Novel Natural-like Polycyclic Ring Systems
Source: Molecules. 2022 Nov 22;27(23):8130. doi: 10.3390/molecules27238130 (PMC9735558; doi:10.3390/molecules27238130)

ELECTRONIC SUPPORTING INFORMATION FOR

One-pot sequence of Staudinger/aza-Wittig/Castagnoli-Cushman reactions provides facile access to novel natural-like polycyclic ring system

Rodion Lebedev [1], Dmitry Dar'in [1], Grigory Kantin [1], Olga Bakulina [1], and Mikhail Krasavin [1,2*]

[1] Saint Petersburg State University, 26 Universitetskii prospect, Peterhof 198504 Russian Federation

[2] Immanuel Kant Baltic Federal University, Kaliningrad 236041 Russian Federation
* Correspondence: m.krasavin@spbu.ru (M.K.), URL: http://krasavin-group.org

Table of contents

Crystallographic data S2
References S2
Copies of NMR spectra S11

Crystallographic data

X-ray Single Crystal analysis was performed on Rigaku XtaLAB Synergy-S diffractometer with monochromated CuKα radiation. Crystal growth was performed by slow dilution of solution in DMSO at room temperature. The crystal was kept at 100 K during data collection. Using Olex2 [1], the structures were solved with the SHELXT [2] structure solution program using Intrinsic Phasing and refined with the SHELXL[3] refinement package using Least Squares minimization. Crystallographic data: CCDC 2207932 (**6a**), 2207933 (**6c**), 2207934 (**6d**), 2207935 (**6e**), 2207937 (**6f**), 2178410 (**6m**), 2207930 (**13**), 2207931 (**16**), 2207936 (**6a'**) contain the supplementary crystallographic data for this paper. These data can be obtained free of charge from The Cambridge Crystallographic Data Centre via www.ccdc.cam.ac.uk/structures.

References

1. Dolomanov, O. V.; Bourhis, L. J.; Gildea, R. J.; Howard, J. A. K.; Puschmann, H., OLEX2: a complete structure solution, refinement and analysis program. Journal of Applied Crystallography 2009, 42, (2), 339-341, 10.1107/s0021889808042726.
2. Sheldrick, G. M., SHELXT - integrated space-group and crystal-structure determination. Acta Crystallogr A Found Adv 2015, 71, (Pt 1), 3-8, 10.1107/S2053273314026370.
3. Sheldrick, G. M., Crystal structure refinement with SHELXL. Acta Crystallogr C Struct Chem 2015, 71, (Pt 1), 3-8, 10.1107/S2053229614024218.

**Table S1. Crystal data and ORTEP representation for 6a (CCDC 2207932)**	
**Empirical formula**	$C_{18}H_{15}NO_4$
**Formula weight**	309.31
**Temperature/K**	100.15
**Crystal system**	triclinic
**Space group**	P-1
**a/Å**	8.8480(4)
**b/Å**	8.8775(4)
**c/Å**	11.0352(5)
**α/°**	68.579(4)
**β/°**	66.496(4)
**γ/°**	65.896(4)
**Volume/Å^3**	704.05(6)
**Z**	2
**ρ_{calc}g/cm^3**	1.459
**μ/mm^{-1}**	0.857
**F(000)**	324.0
**Crystal size/mm^3**	0.1 × 0.08 × 0.04
**Radiation**	CuKα (λ = 1.54184)
**2Θ range for data collection/°**	9.006 to 138.454
**Index ranges**	$-10 \leq h \leq 10$, $-10 \leq k \leq 10$, $-11 \leq l \leq 13$
**Reflections collected**	5951
**Independent reflections**	2625 [R_{int} = 0.0194, R_{sigma} = 0.0276]
**Data/restraints/parameters**	2625/0/209
**Goodness-of-fit on F^2**	1.055
**Final R indexes [I>=2σ (I)]**	R_1 = 0.0336, wR_2 = 0.0872
**Final R indexes [all data]**	R_1 = 0.0378, wR_2 = 0.0904
**Largest diff. peak/hole / e Å^{-3}**	0.26/-0.21

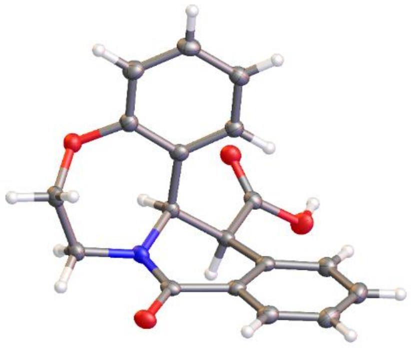

Figure S1. ORTEP representation of compound **6a** (thermal ellipsoids are shown at 50% probability)

Table S2. Crystal data and ORTEP representation for 6d (2207933)	
**Empirical formula**	$C_{18}H_{14}ClNO_4$
**Formula weight**	343.75
**Temperature/K**	100.15
**Crystal system**	triclinic
**Space group**	P-1
**a/Å**	8.7472(6)
**b/Å**	9.9319(8)
**c/Å**	10.2459(8)
**α/°**	69.488(7)
**β/°**	69.344(7)
**γ/°**	72.157(7)
**Volume/Å³**	762.94(11)
**Z**	2
**ρ_{calc}g/cm³**	1.496
**μ/mm⁻¹**	2.427
**F(000)**	356
**Crystal size/mm³**	0.1 × 0.07 × 0.03
**Radiation**	CuKα (λ = 1.54184)
**2Θ range for data collection/°**	9.586 to 152.44
**Index ranges**	-10 ≤ h ≤ 10, -8 ≤ k ≤ 12, -12 ≤ l ≤ 12
**Reflections collected**	5927
**Independent reflections**	3117 [R_{int} = 0.0360, R_{sigma} = 0.0415]
**Data/restraints/parameters**	3117/0/218
**Goodness-of-fit on F²**	1.058
**Final R indexes [I>=2σ (I)]**	R1 = 0.0396, wR2 = 0.1018
**Final R indexes [all data]**	R1 = 0.0487, wR2 = 0.1087
**Largest diff. peak/hole / e Å⁻³**	0.34/-0.40

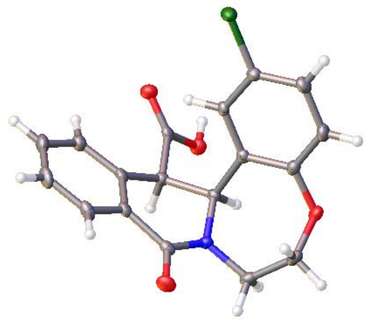

Figure S2. ORTEP representation of compound **6c** (thermal ellipsoids are shown at 50% probability)

Table S3. Crystal data and ORTEP representation for 6d (2207934)	
**Empirical formula**	$C_{20}H_{20}BrNO_5S$
**Formula weight**	466.34
**Temperature/K**	100.15
**Crystal system**	triclinic
**Space group**	P-1
**a/Å**	9.4897(5)
**b/Å**	9.8117(5)
**c/Å**	11.0978(4)
**α/°**	98.873(4)
**β/°**	97.974(4)
**γ/°**	98.680(5)
**Volume/Å^3**	995.28(8)
**Z**	2
**ρ_{calc}g/cm^3**	1.556
**μ/mm^{-1}**	4.074
**F(000)**	476
**Crystal size/mm^3**	0.2 × 0.16 × 0.1
**Radiation**	CuKα (λ = 1.54184)
**2Θ range for data collection/°**	8.176 to 152.868
**Index ranges**	$-11 \leq h \leq 11, -12 \leq k \leq 11, -13 \leq l \leq 8$
**Reflections collected**	7904
**Independent reflections**	4058 [R_{int} = 0.0323, R_{sigma} = 0.0387]
**Data/restraints/parameters**	4058/0/256
**Goodness-of-fit on F^2**	1.036
**Final R indexes [I>=2σ (I)]**	R_1 = 0.0321, wR_2 = 0.0811
**Final R indexes [all data]**	R_1 = 0.0379, wR_2 = 0.0859
**Largest diff. peak/hole / e Å^{-3}**	0.47/-0.72

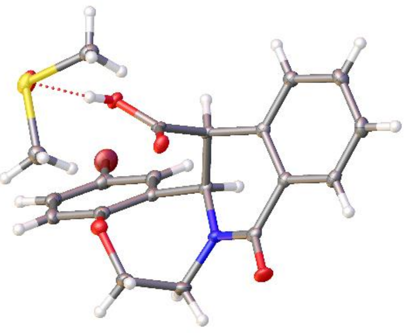

Figure S3. ORTEP representation of compound **6d** (thermal ellipsoids are shown at 50% probability)

Table S4. Crystal data and ORTEP representation for 6e (2207935)	
**Empirical formula**	$C_{24}H_{18.67}N_{2.67}O_8$
**Formula weight**	472.41
**Temperature/K**	100.15
**Crystal system**	monoclinic
**Space group**	Cc
**a/Å**	14.1681(3)
**b/Å**	12.9329(3)
**c/Å**	8.3714(2)
**α/°**	90
**β/°**	94.263(2)
**γ/°**	90
**Volume/Å^3**	1529.69(6)
**Z**	3
**ρ_{calc}g/cm^3**	1.538
**μ/mm^{-1}**	0.993
**F(000)**	736
**Crystal size/mm^3**	? × ? × ?
**Radiation**	CuKα (λ = 1.54184)
**2Θ range for data collection/°**	9.27 to 160.626
**Index ranges**	$-17 \le h \le 17, -16 \le k \le 16, -10 \le l \le 10$
**Reflections collected**	7704
**Independent reflections**	2624 [R_{int} = 0.0396, R_{sigma} = 0.0400]
**Data/restraints/parameters**	2624/2/236
**Goodness-of-fit on F^2**	1.094
**Final R indexes [I>=2σ (I)]**	R_1 = 0.0326, wR_2 = 0.0857
**Final R indexes [all data]**	R_1 = 0.0346, wR_2 = 0.0867
**Largest diff. peak/hole / e Å^{-3}**	0.20/-0.17

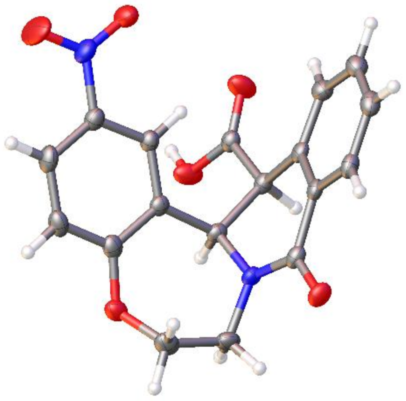

Figure S4. ORTEP representation of compound **6e** (thermal ellipsoids are shown at 50% probability)

Table S5. Crystal data and ORTEP representation for 6f (2207937)	
**Empirical formula**	$C_{20}H_{22}NO_{6.5}S_{0.5}$
**Formula weight**	396.41
**Temperature/K**	100.15
**Crystal system**	monoclinic
**Space group**	C2
**a/Å**	24.8119(3)
**b/Å**	7.35890(10)
**c/Å**	20.2825(3)
**α/°**	90
**β/°**	97.5290(10)
**γ/°**	90
**Volume/Å^3**	3671.42(9)
**Z**	8
**ρ_{calc}g/cm^3**	1.434
**μ/mm^{-1}**	1.405
**F(000)**	1672
**Crystal size/mm^3**	0.15 × 0.09 × 0.05
**Radiation**	CuKα (λ = 1.54184)
**2Θ range for data collection/°**	7.188 to 152.338
**Index ranges**	$-31 \leq h \leq 31$, $-9 \leq k \leq 8$, $-25 \leq l \leq 22$
**Reflections collected**	8474
**Independent reflections**	5471 [R_{int} = 0.0332, R_{sigma} = 0.0380]
**Data/restraints/parameters**	5471/1/517
**Goodness-of-fit on F^2**	1.05
**Final R indexes [I>=2σ (I)]**	R_1 = 0.0362, wR_2 = 0.0958
**Final R indexes [all data]**	R_1 = 0.0386, wR_2 = 0.0979
**Largest diff. peak/hole / e Å^{-3}**	0.51/-0.37

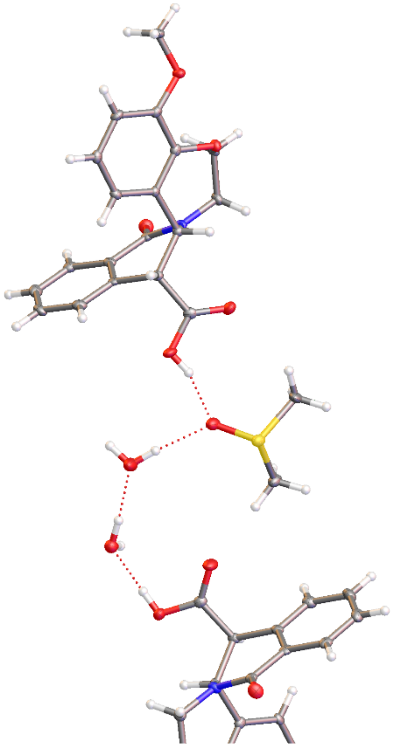

Figure S5. ORTEP representation of compound **6f** (thermal ellipsoids are shown at 50% probability)

Table S6. Crystal data and ORTEP representation for 6m (2178410)	
**Empirical formula**	$C_{22}H_{17}NO_4$
**Formula weight**	359.36
**Temperature/K**	100.15
**Crystal system**	triclinic
**Space group**	P-1
**a/Å**	8.3167(3)
**b/Å**	9.0975(4)
**c/Å**	11.7175(7)
**α/°**	73.755(4)
**β/°**	75.582(4)
**γ/°**	87.460(3)
**Volume/Å^3**	824.05(7)
**Z**	2
**ρ_{calc}g/cm^3**	1.448
**μ/mm^{-1}**	0.82
**F(000)**	376
**Crystal size/mm^3**	0.16 × 0.12 × 0.04
**Radiation**	CuKα (λ = 1.54184)
**2Θ range for data collection/°**	8.11 to 160.482
**Index ranges**	$-8 \leq h \leq 10$, $-11 \leq k \leq 11$, $-14 \leq l \leq 14$
**Reflections collected**	10082
**Independent reflections**	3430 [R_{int} = 0.0513, R_{sigma} = 0.0439]
**Data/restraints/parameters**	3430/0/245
**Goodness-of-fit on F^2**	1.122
**Final R indexes [I>=2σ (I)]**	R_1 = 0.0982, wR_2 = 0.3030
**Final R indexes [all data]**	R_1 = 0.1037, wR_2 = 0.3048
**Largest diff. peak/hole / e Å^{-3}**	0.85/-0.52

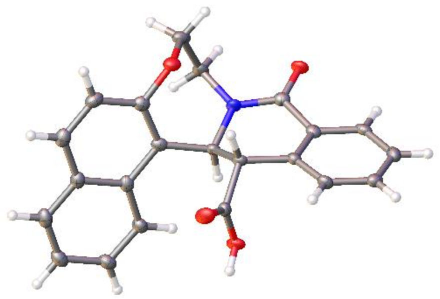

Figure S6. ORTEP representation of compound **6m** (thermal ellipsoids are shown at 50% probability)

Table S7. Crystal data and ORTEP representation for 13 (2207930)

**Empirical formula**	$C_{16.8}H_{16}N_{1.6}O_{3.2}$
**Formula weight**	291.51
**Temperature/K**	100.15
**Crystal system**	monoclinic
**Space group**	Ia
**a/Å**	13.1227(3)
**b/Å**	8.3329(2)
**c/Å**	16.2683(3)
**α/°**	90
**β/°**	93.697(2)
**γ/°**	90
**Volume/Å^3**	1775.24(7)
**Z**	5
**ρ_{calc}g/cm^3**	1.363
**μ/mm^{-1}**	0.78
**F(000)**	768
**Crystal size/mm^3**	0.28 × 0.18 × 0.12
**Radiation**	CuKα (λ = 1.54184)
**2Θ range for data collection/°**	10.9 to 152.632
**Index ranges**	$-13 \leq h \leq 16$, $-10 \leq k \leq 10$, $-19 \leq l \leq 20$
**Reflections collected**	7455
**Independent reflections**	2852 [R_{int} = 0.0213, R_{sigma} = 0.0208]
**Data/restraints/parameters**	2852/2/246
**Goodness-of-fit on F^2**	1.057
**Final R indexes [I>=2σ (I)]**	R_1 = 0.0288, wR_2 = 0.0756
**Final R indexes [all data]**	R_1 = 0.0289, wR_2 = 0.0758
**Largest diff. peak/hole / e Å^{-3}**	0.30/-0.17

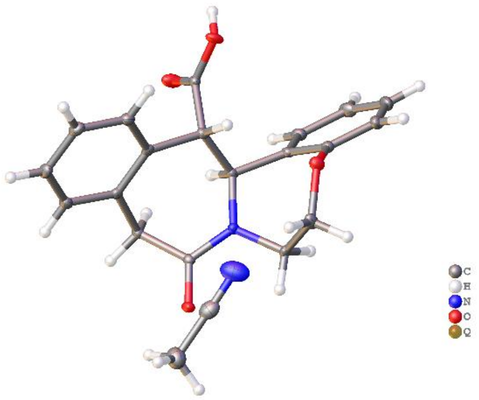

Figure S7. ORTEP representation of compound **13** (thermal ellipsoids are shown at 50% probability)

Table S8. Crystal data and ORTEP representation for 16 (2207931)	
**Empirical formula**	$C_{13}H_{13}NO_4S$
**Formula weight**	279.3
**Temperature/K**	100.15
**Crystal system**	monoclinic
**Space group**	$P2_1/c$
**a/Å**	13.3435(2)
**b/Å**	8.09740(10)
**c/Å**	11.9013(2)
**α/°**	90
**β/°**	109.952(2)
**γ/°**	90
**Volume/Å^3**	1208.73(3)
**Z**	4
**ρ_{calc}g/cm^3**	1.535
**μ/mm^{-1}**	2.494
**F(000)**	584
**Crystal size/mm^3**	0.14 × 0.12 × 0.08
**Radiation**	CuKα (λ = 1.54184)
**2Θ range for data collection/°**	7.048 to 138.17
**Index ranges**	$-16 \leq h \leq 15$, $-9 \leq k \leq 9$, $-14 \leq l \leq 14$
**Reflections collected**	5547
**Independent reflections**	2252 [R_{int} = 0.0271, R_{sigma} = 0.0261]
**Data/restraints/parameters**	2252/0/173
**Goodness-of-fit on F^2**	1.051
**Final R indexes [I>=2σ (I)]**	R_1 = 0.0319, wR_2 = 0.0858
**Final R indexes [all data]**	R_1 = 0.0329, wR_2 = 0.0869
**Largest diff. peak/hole / e Å^{-3}**	0.27/-0.28

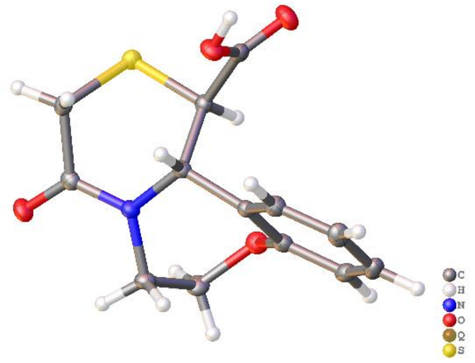

Figure S8. ORTEP representation of compound **16** (thermal ellipsoids are shown at 50% probability)

Table S9. Crystal data and ORTEP representation for 6a' (2207936)	
**Empirical formula**	$C_{18}H_{15}NO_4$
**Formula weight**	309.31
**Temperature/K**	100.15
**Crystal system**	monoclinic
**Space group**	$P2_1/c$
**a/Å**	10.9679(3)
**b/Å**	9.2236(2)
**c/Å**	14.4744(3)
**α/°**	90
**β/°**	100.629(2)
**γ/°**	90
**Volume/Å^3**	1439.16(6)
**Z**	4
**ρ_{calc}g/cm^3**	1.428
**μ/mm^{-1}**	0.839
**F(000)**	648.0
**Crystal size/mm^3**	0.06 × 0.04 × 0.02
**Radiation**	CuKα (λ = 1.54184)
**2Θ range for data collection/°**	8.202 to 159.218
**Index ranges**	$-13 \leq h \leq 13$, $-10 \leq k \leq 11$, $-18 \leq l \leq 18$
**Reflections collected**	10838
**Independent reflections**	3036 [R_{int} = 0.0495, R_{sigma} = 0.0406]
**Data/restraints/parameters**	3036/0/209
**Goodness-of-fit on F^2**	1.035
**Final R indexes [I>=2σ (I)]**	R_1 = 0.0415, wR_2 = 0.1040
**Final R indexes [all data]**	R_1 = 0.0487, wR_2 = 0.1081
**Largest diff. peak/hole / e Å^{-3}**	0.22/-0.23

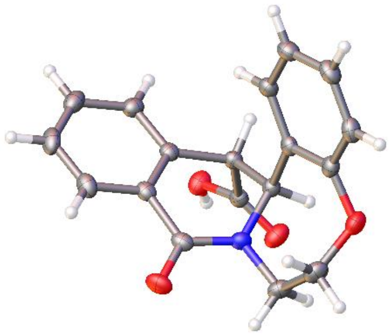

Figure S9. ORTEP representation of compound **6a'** (thermal ellipsoids are shown at 50% probability)

Copies of ^{1}H 400.13 MHz, $CDCl_3$ and ^{13}C 100.61 MHz, $CDCl_3$ spectra of **1a**

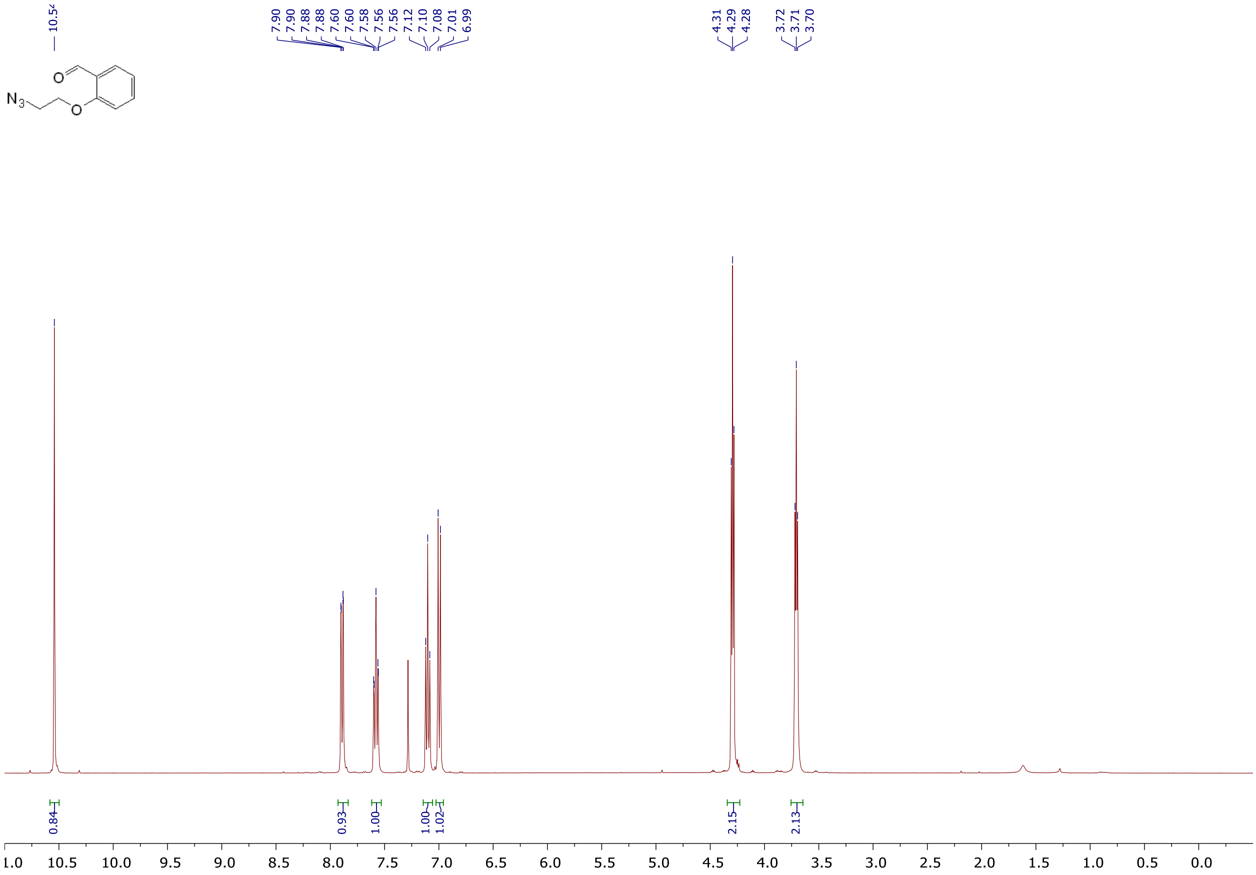

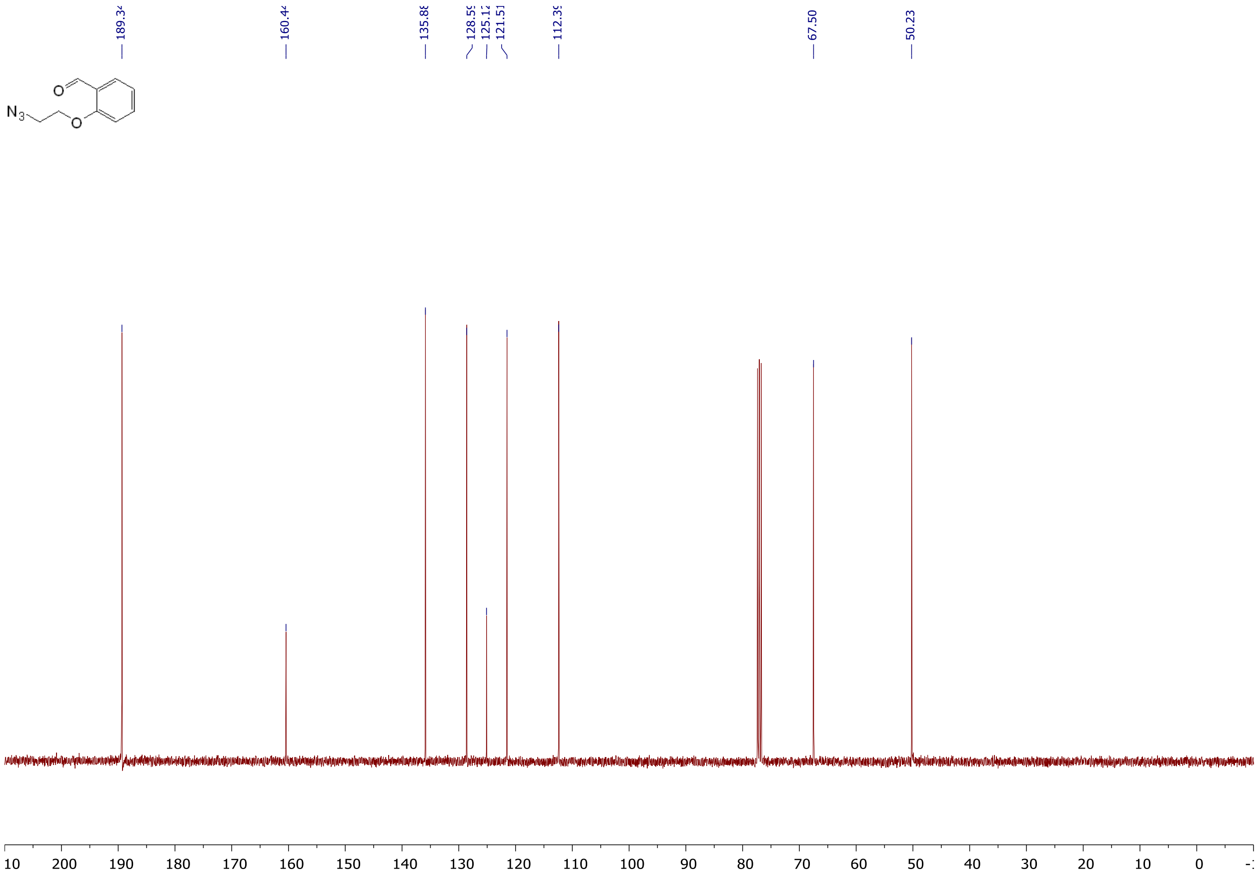

Copies of ^{1}H 400.13 MHz, $CDCl_3$, ^{13}C 100.61 MHz, $CDCl_3$ and ^{19}F 376.50 MHz, $CDCl_3$ spectra of **1b**

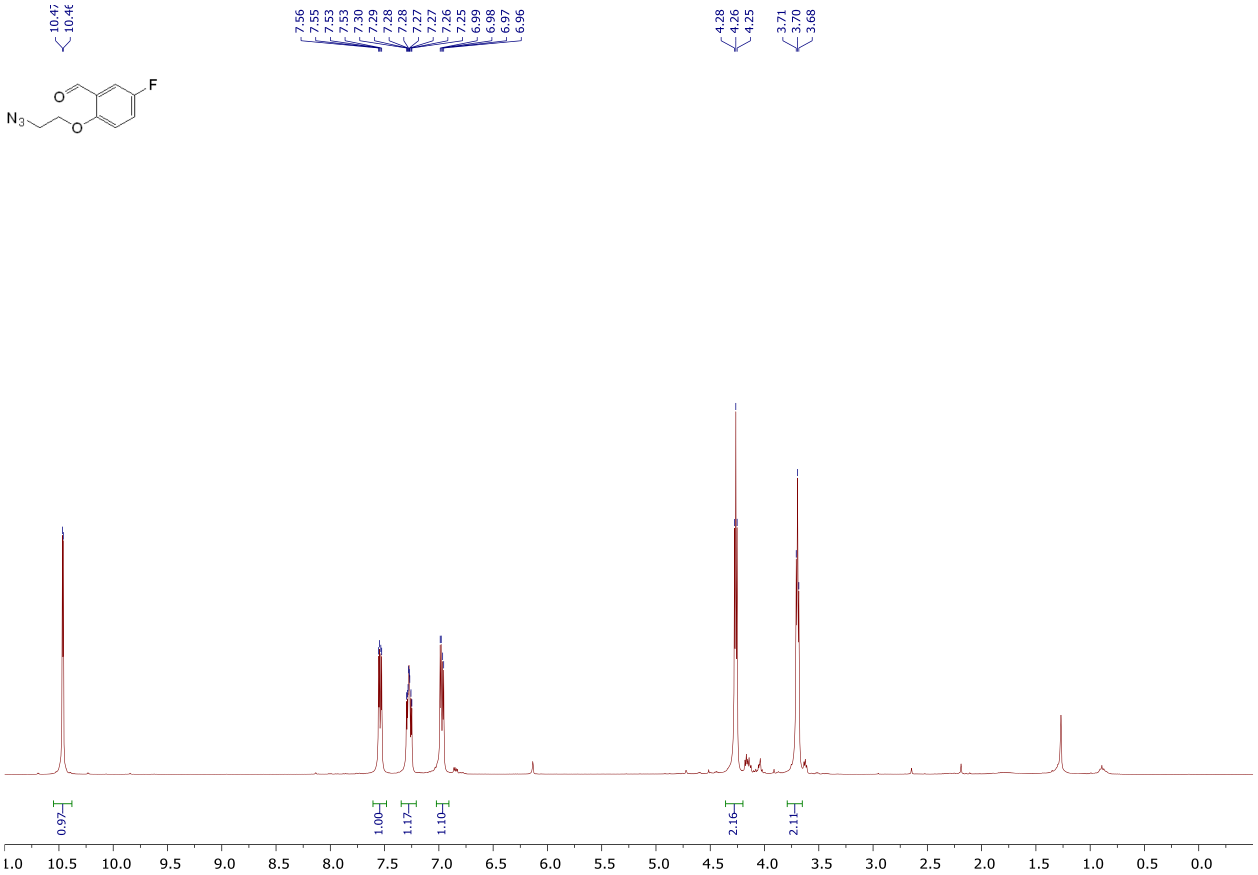

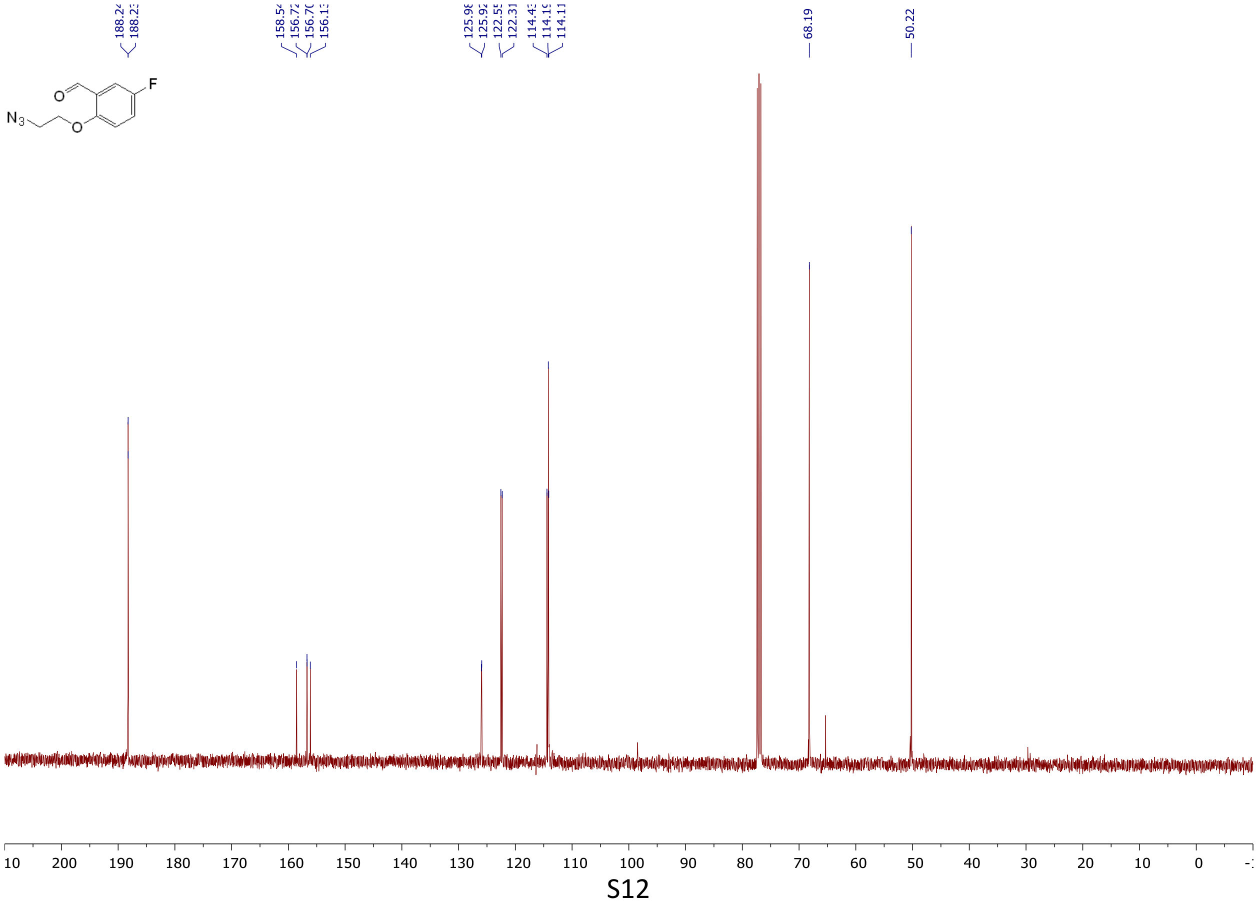

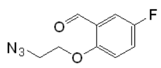
O
F
N_3
O

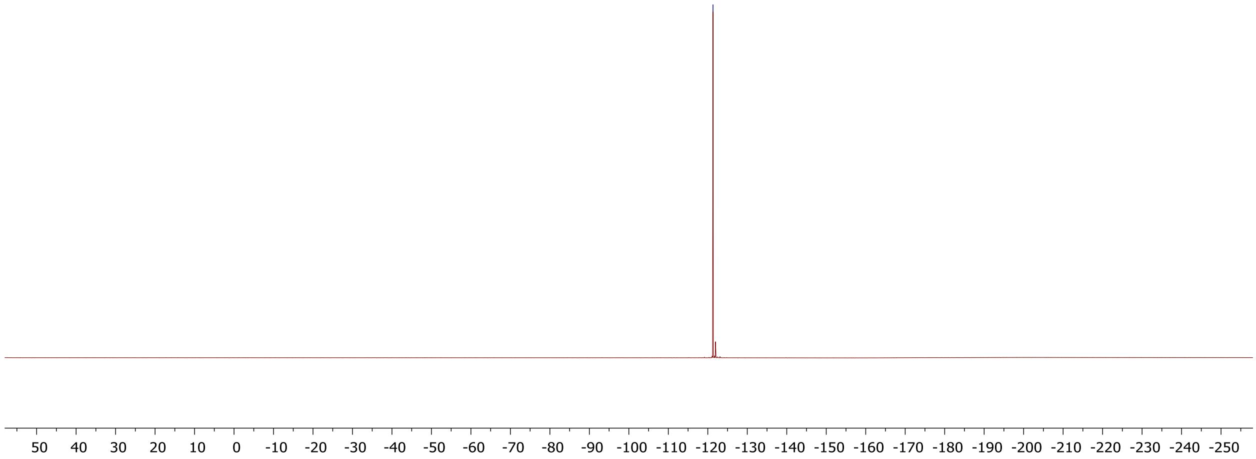
-121.3
50 40 30 20 10 0 -10 -20 -30 -40 -50 -60 -70 -80 -90 -100 -110 -120 -130 -140 -150 -160 -170 -180 -190 -200 -210 -220 -230 -240 -250

Copies of ^{1}H 400.13 MHz, $CDCl_3$ and ^{13}C 100.61 MHz, $CDCl_3$ spectra of **1c**

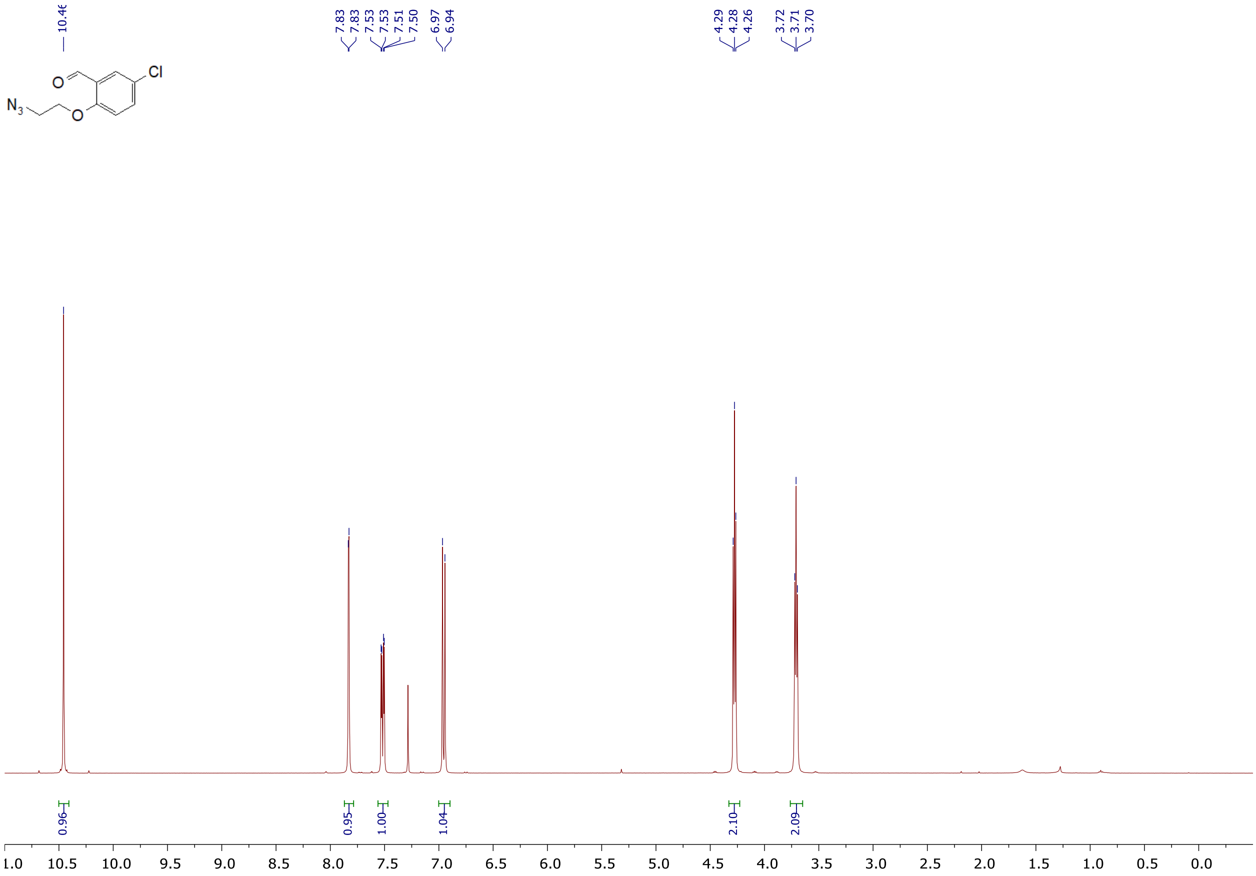

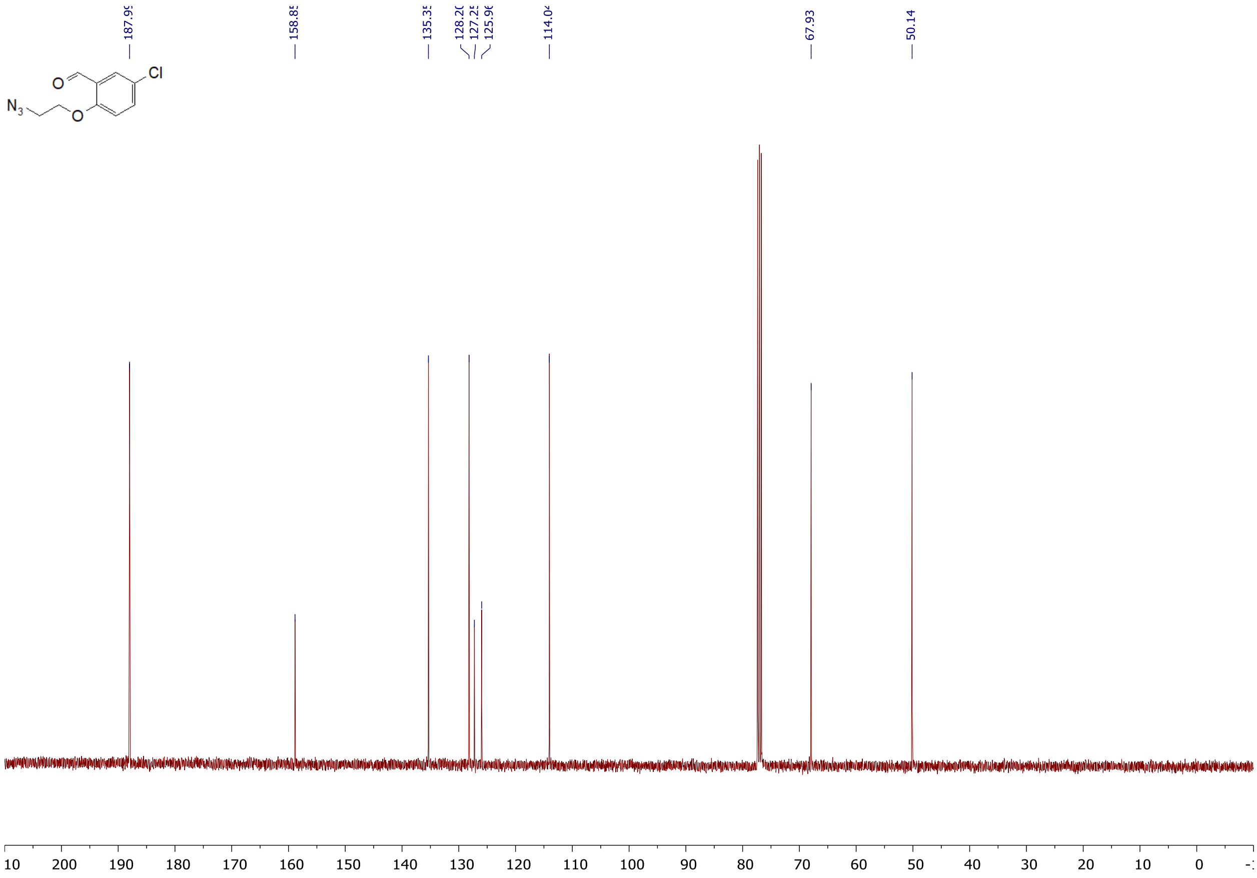

Copies of ^{1}H 400.13 MHz, $CDCl_3$ and ^{13}C 100.61 MHz, $CDCl_3$ spectra of **1d**

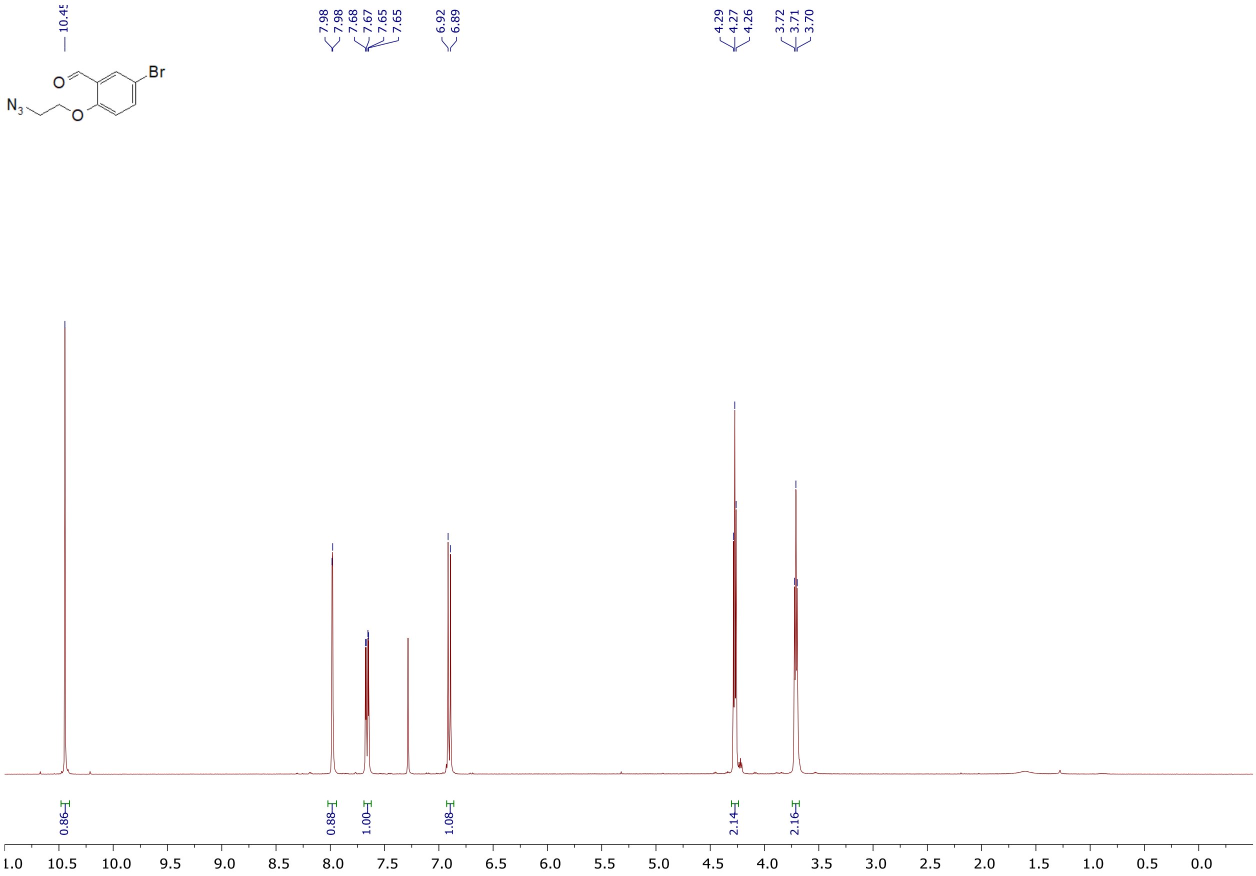

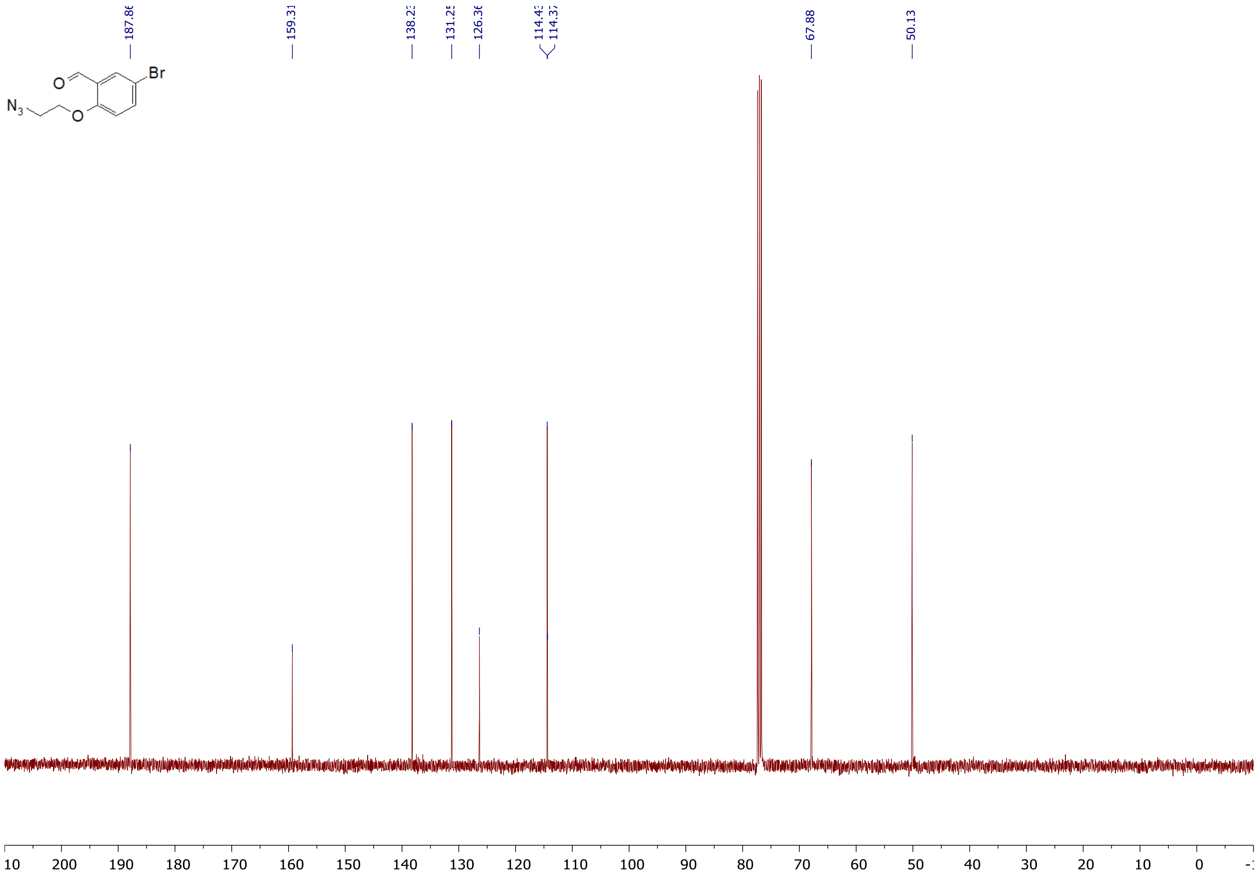

Copies of ^{1}H 400.13 MHz, $CDCl_3$ and ^{13}C 100.61 MHz, $CDCl_3$ spectra of **1e**

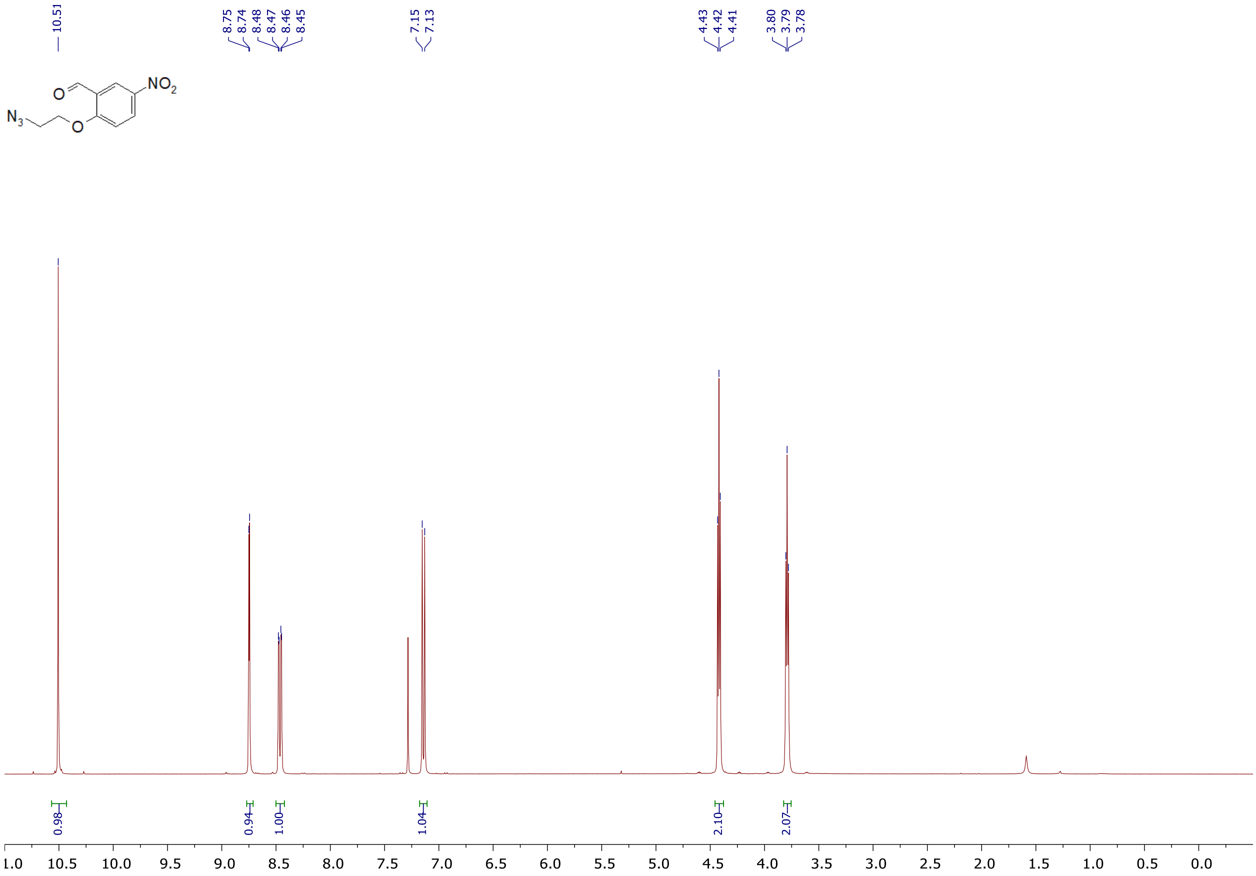

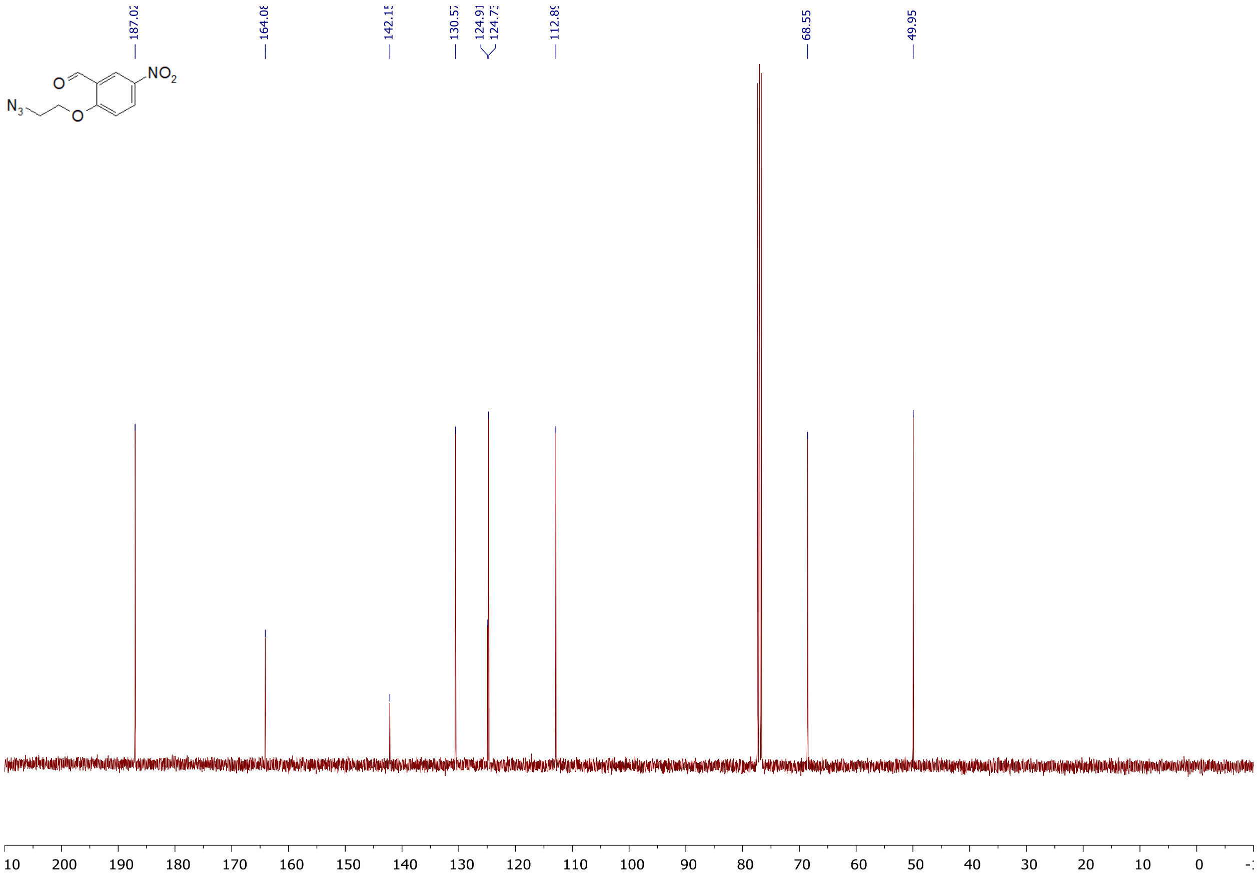

Copies of ^{1}H 400.13 MHz, $CDCl_3$ and ^{13}C 100.61 MHz, $CDCl_3$ spectra of **1f**

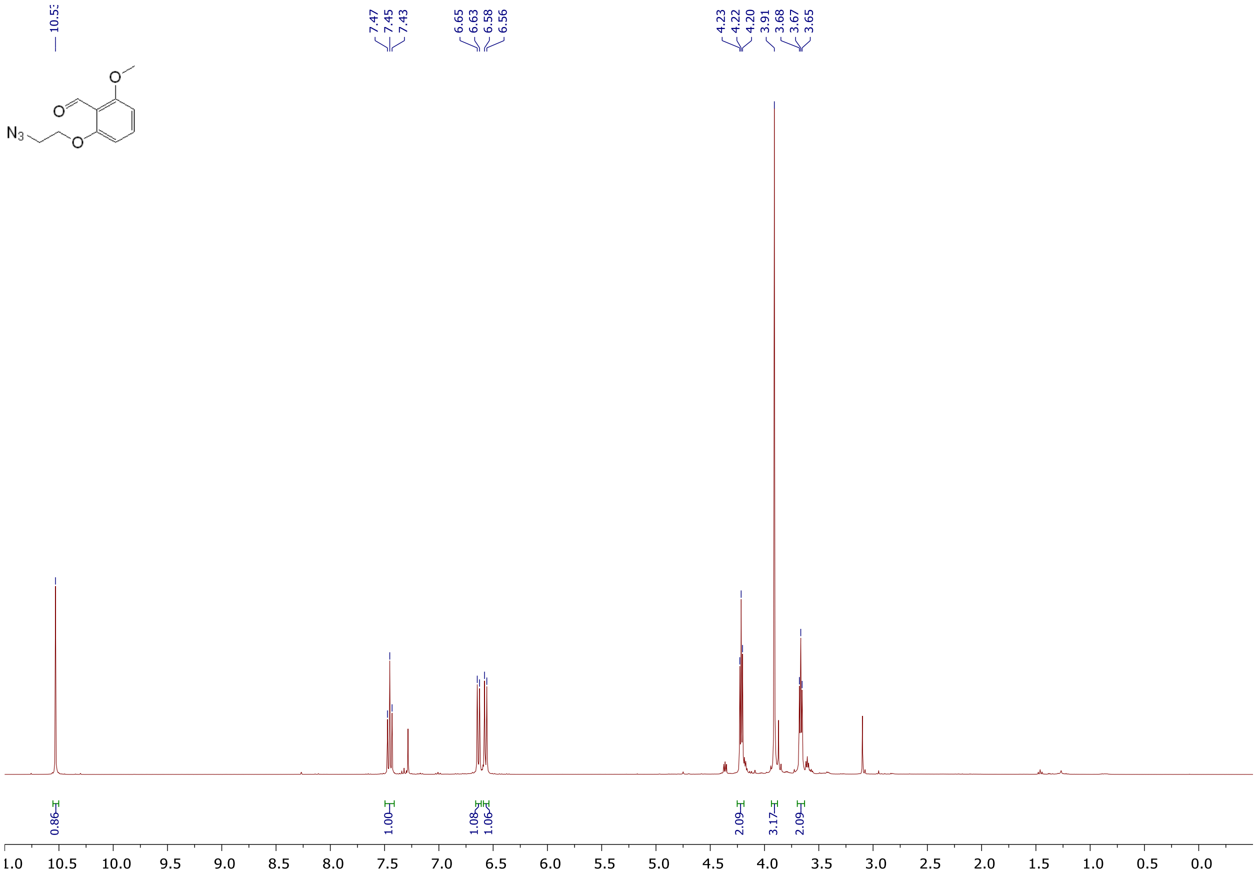

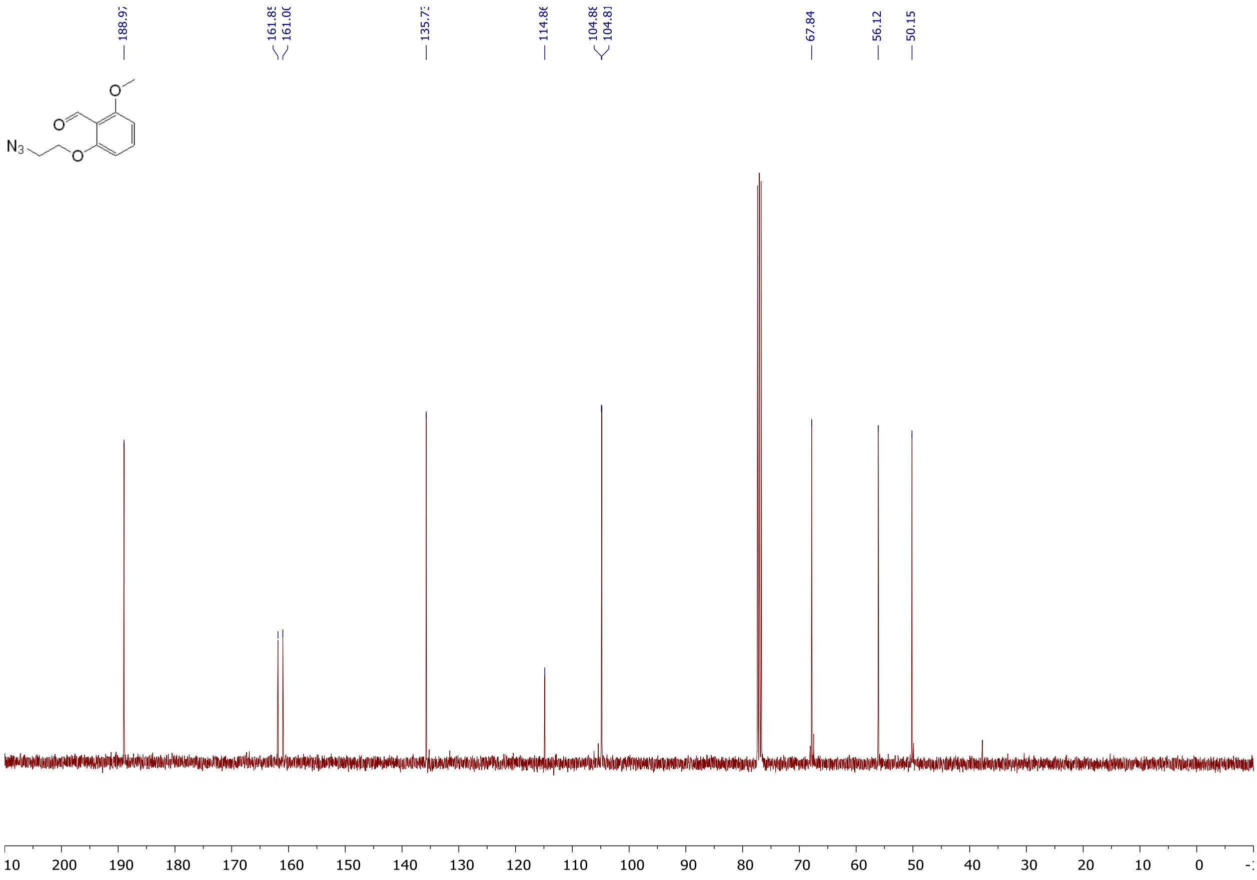

Copies of ^{1}H 400.13 MHz, $CDCl_3$ and ^{13}C 100.61 MHz, $CDCl_3$ spectra of **1g**

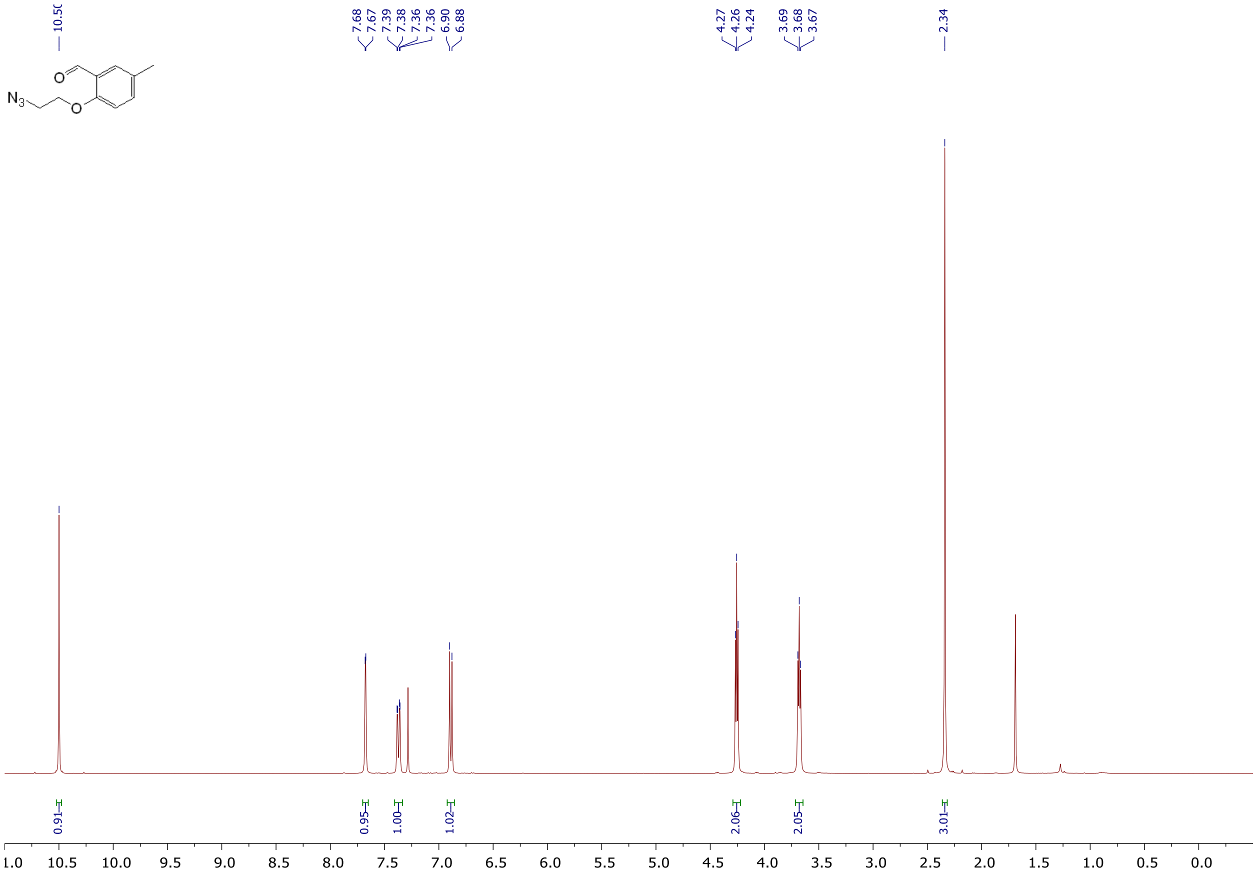

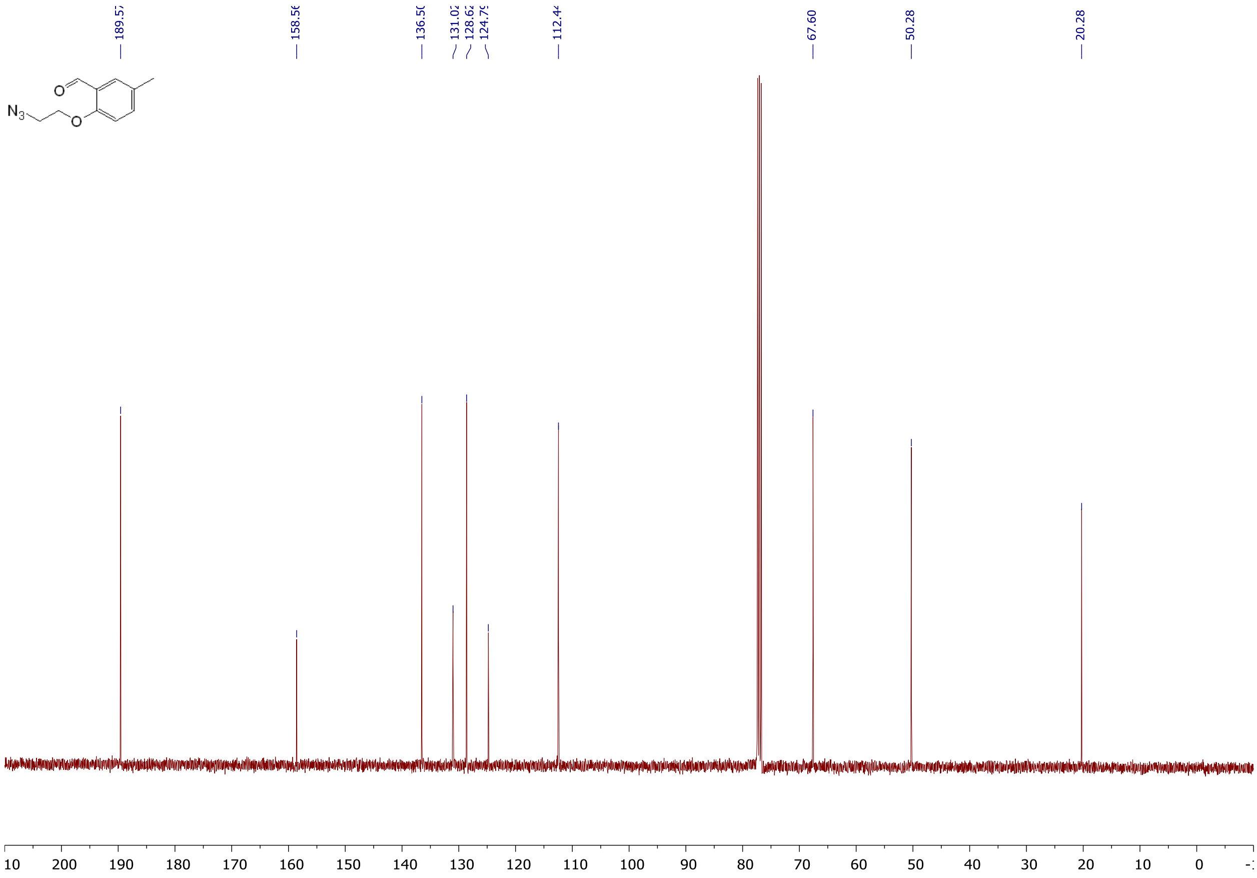

Copies of ^{1}H 400.13 MHz, $CDCl_3$ and ^{13}C 100.61 MHz, $CDCl_3$ spectra of **1h**

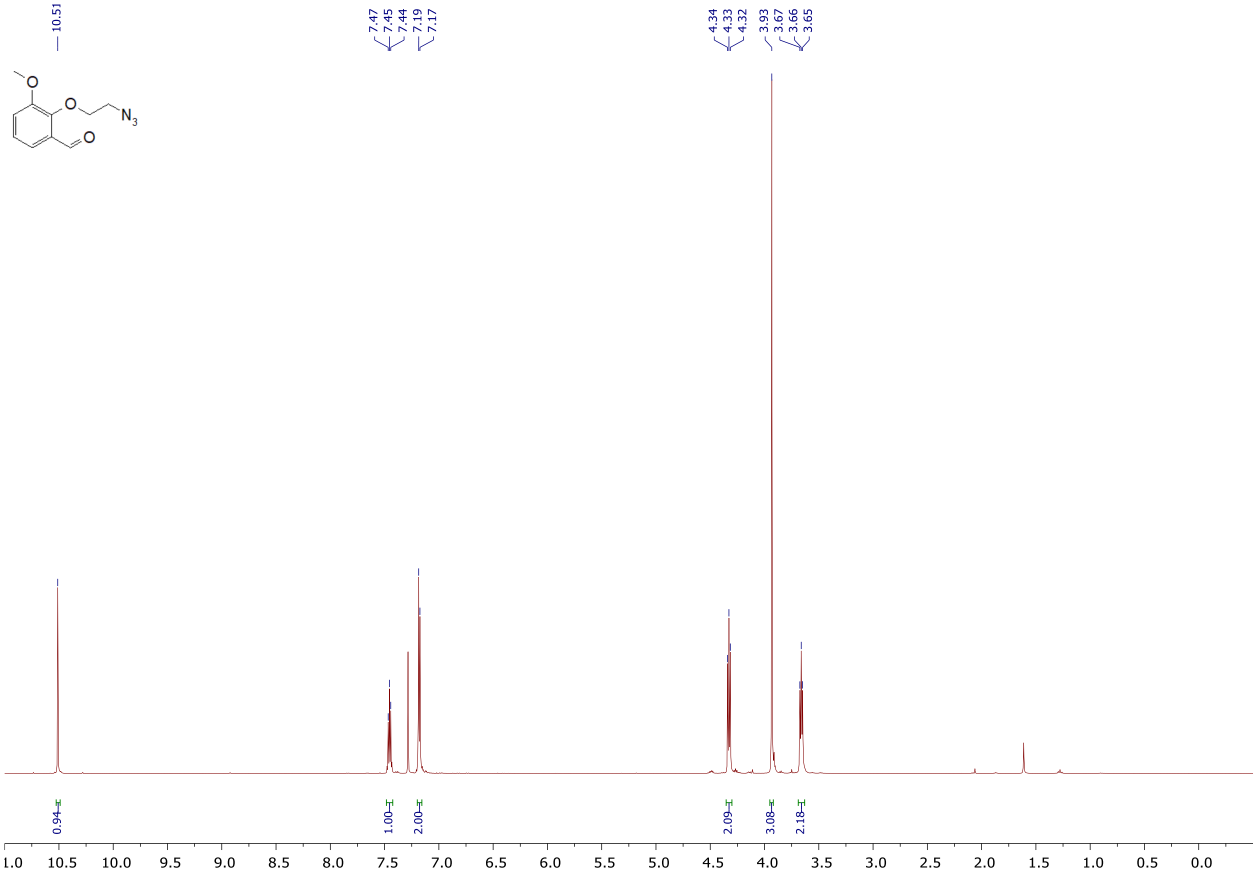

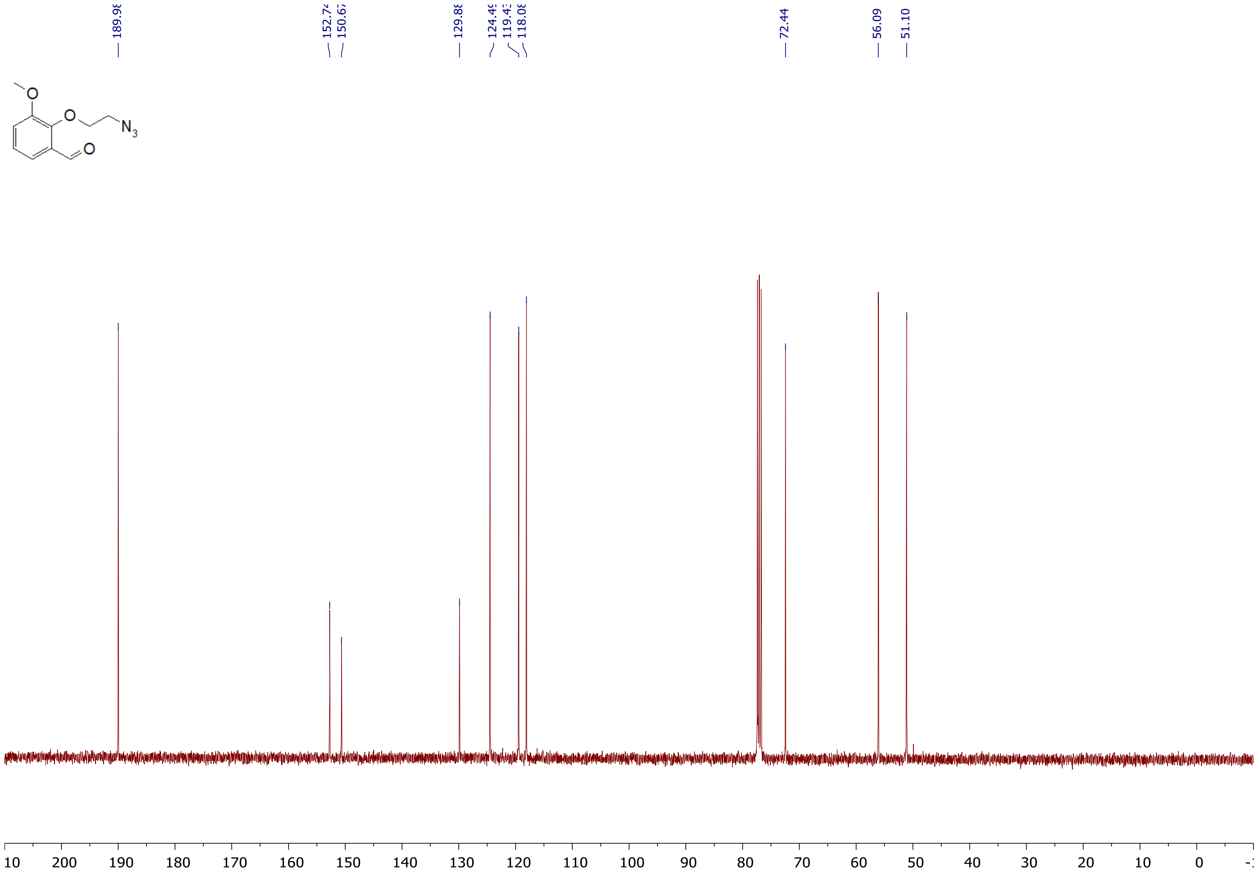

Copies of ^{1}H 400.13 MHz, $CDCl_3$ and ^{13}C 100.61 MHz, $CDCl_3$ spectra of **1i**

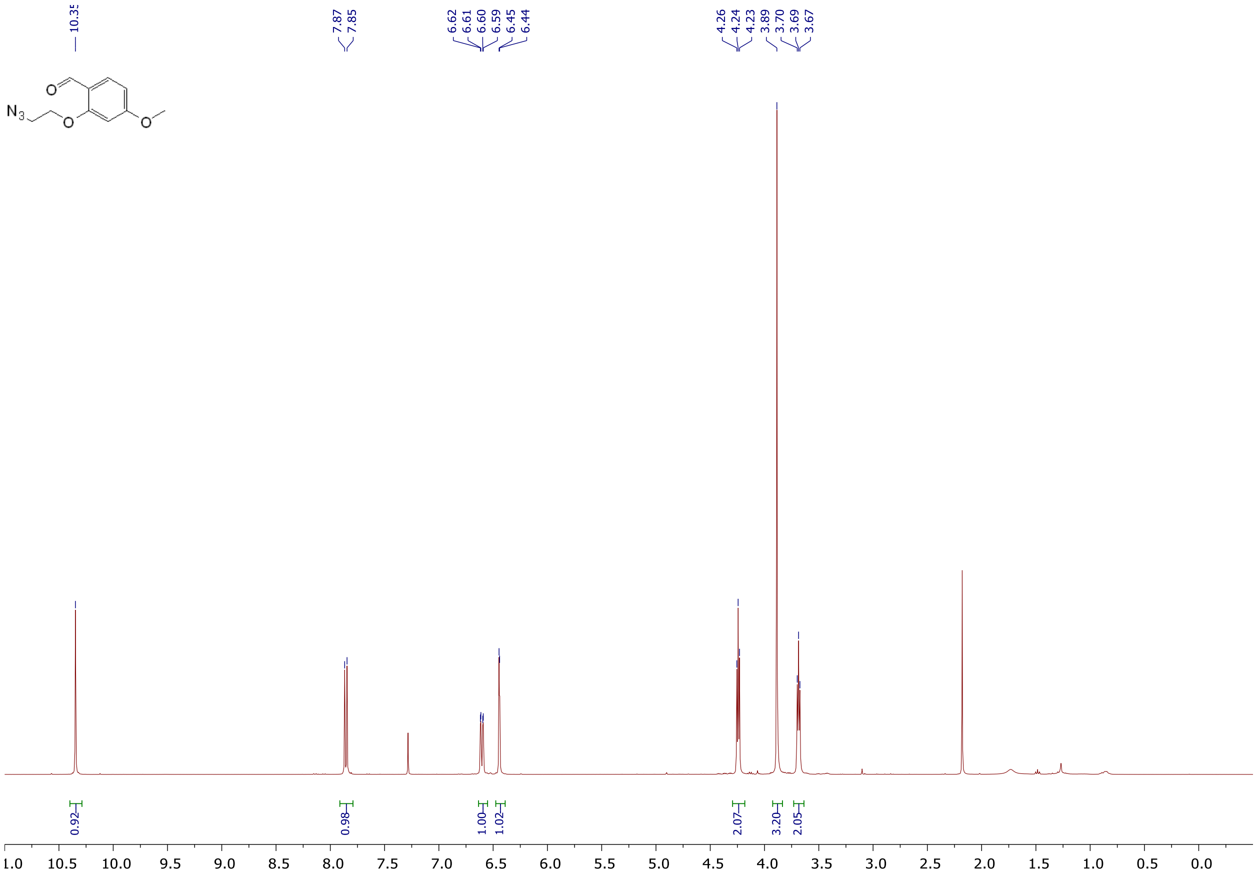

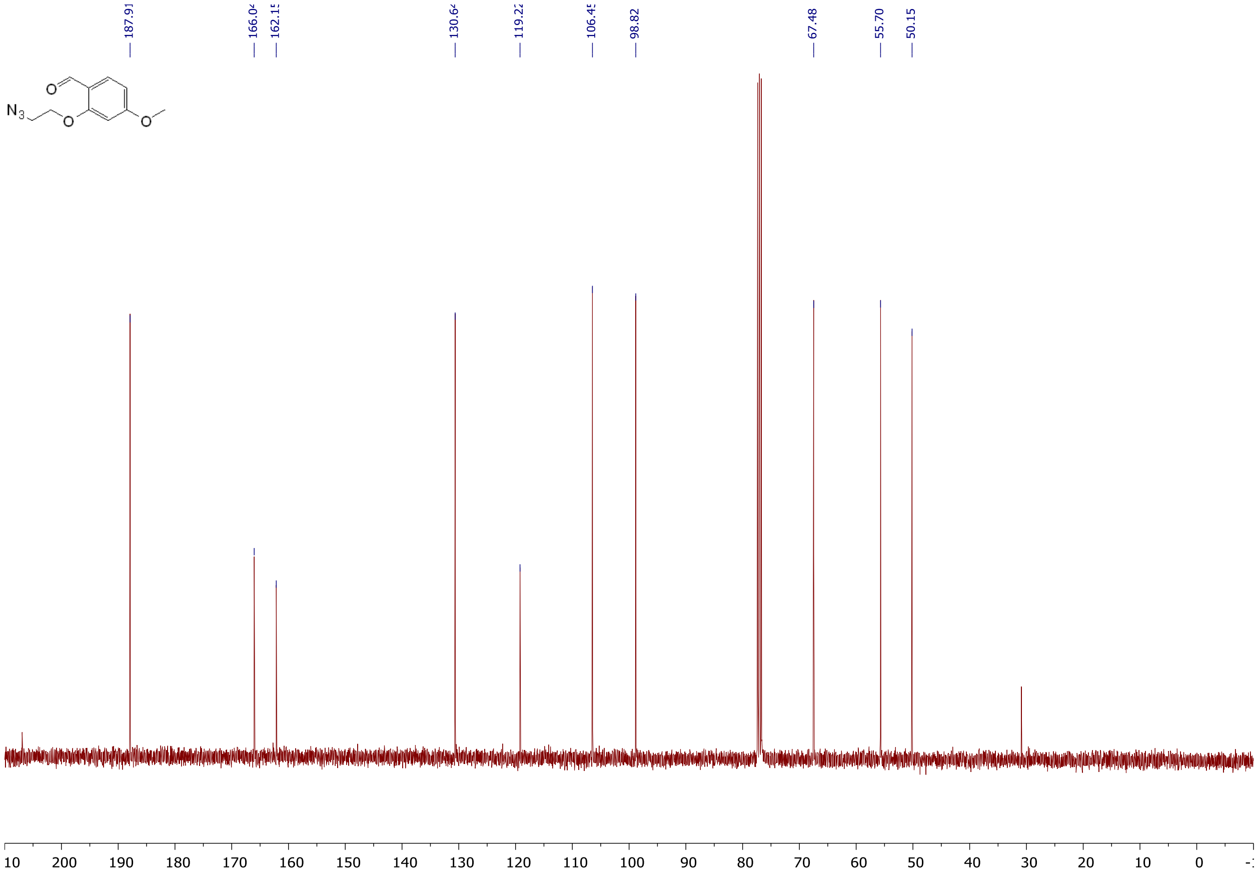

Copies of ^{1}H 400.13 MHz, CDCl$_3$ and ^{13}C 100.61 MHz, CDCl$_3$ spectra of **1j**

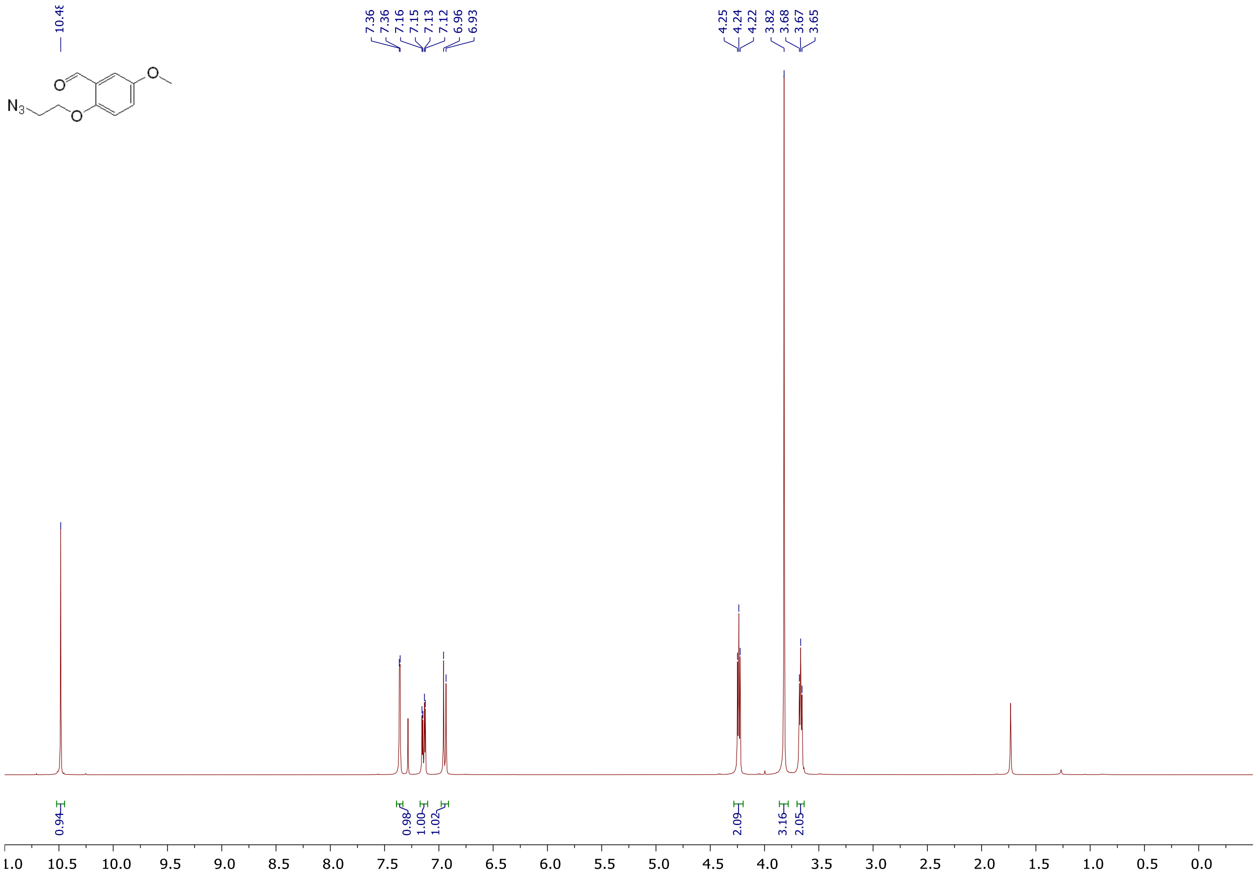

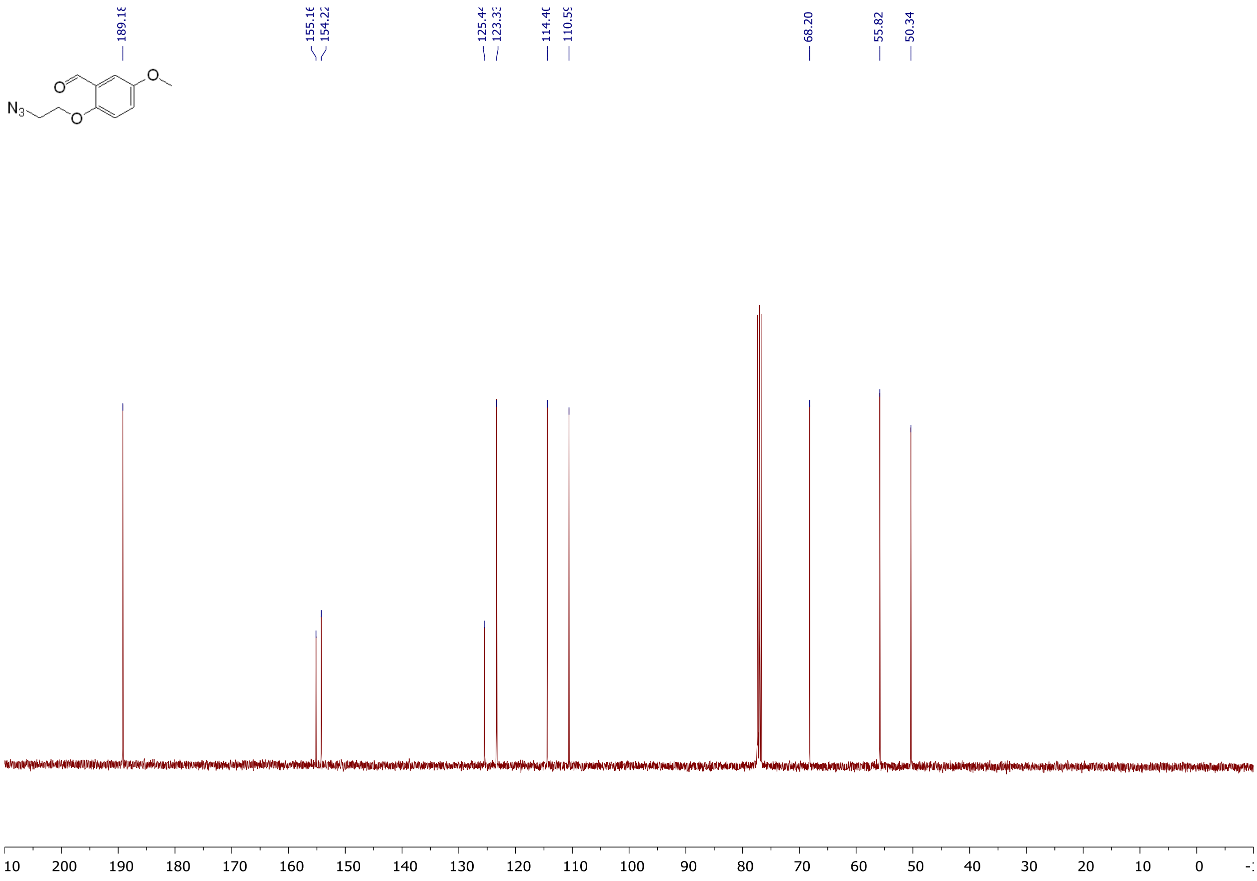

Copies of ^{1}H 400.13 MHz, $CDCl_3$ and ^{13}C 100.61 MHz, $CDCl_3$ spectra of **1k**

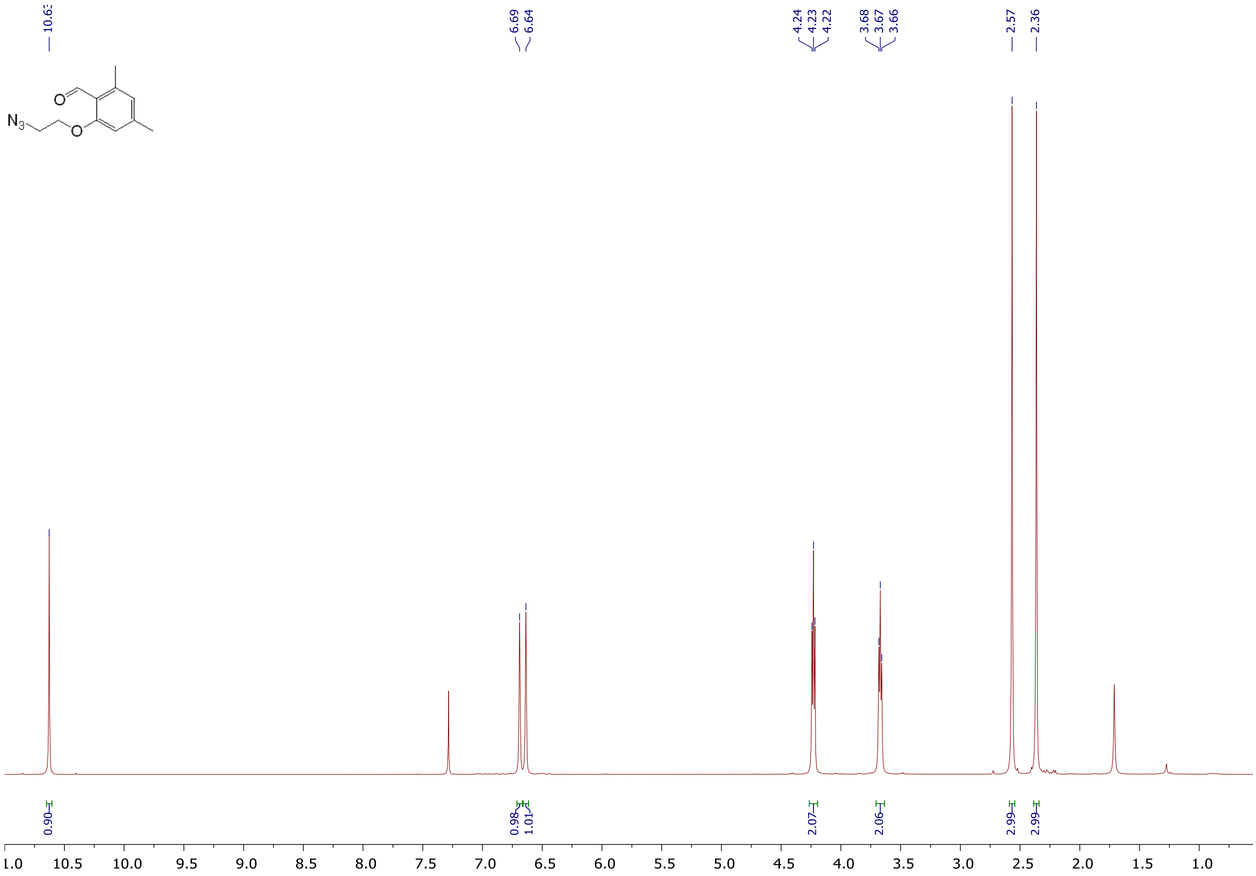

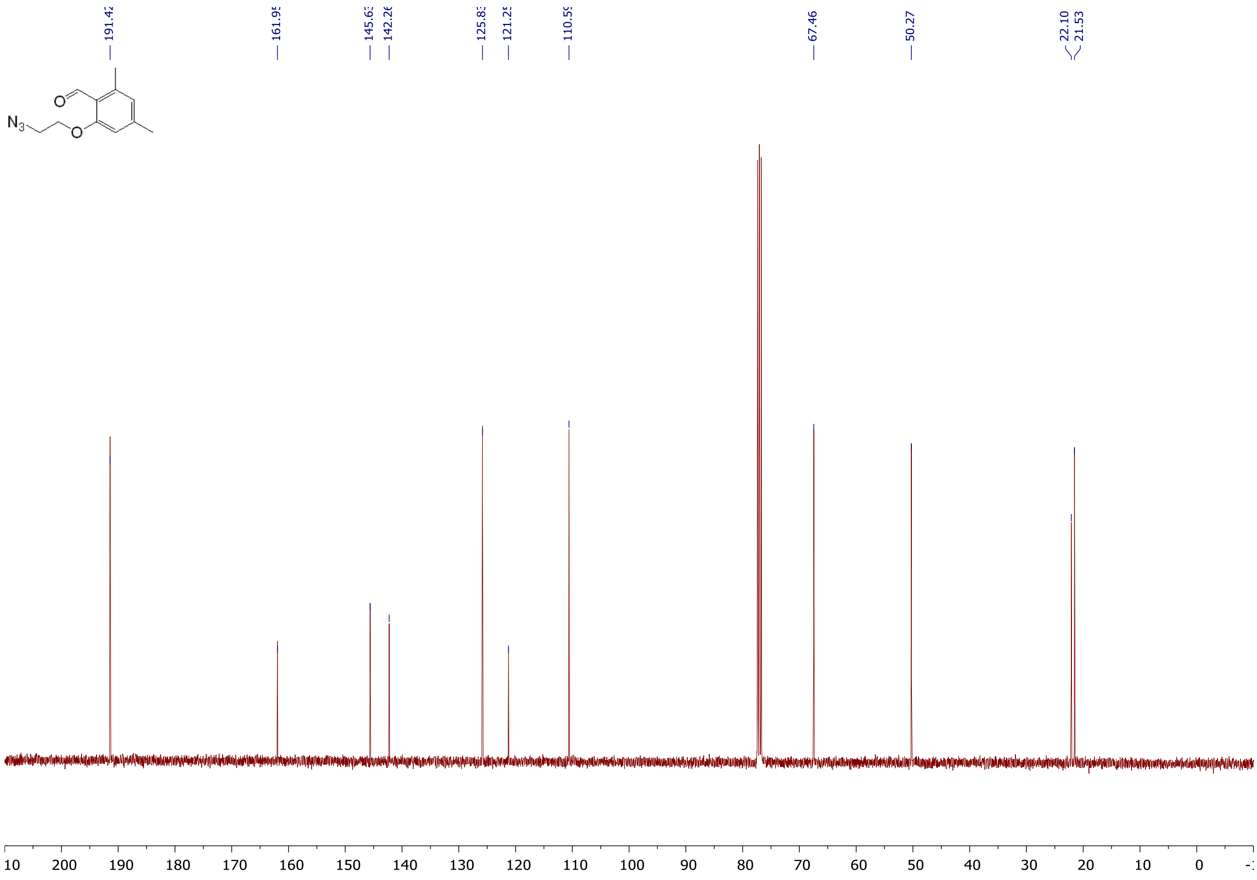

Copies of ^{1}H 400.13 MHz, $CDCl_3$ and ^{13}C 100.61 MHz, $CDCl_3$ spectra of **1l**

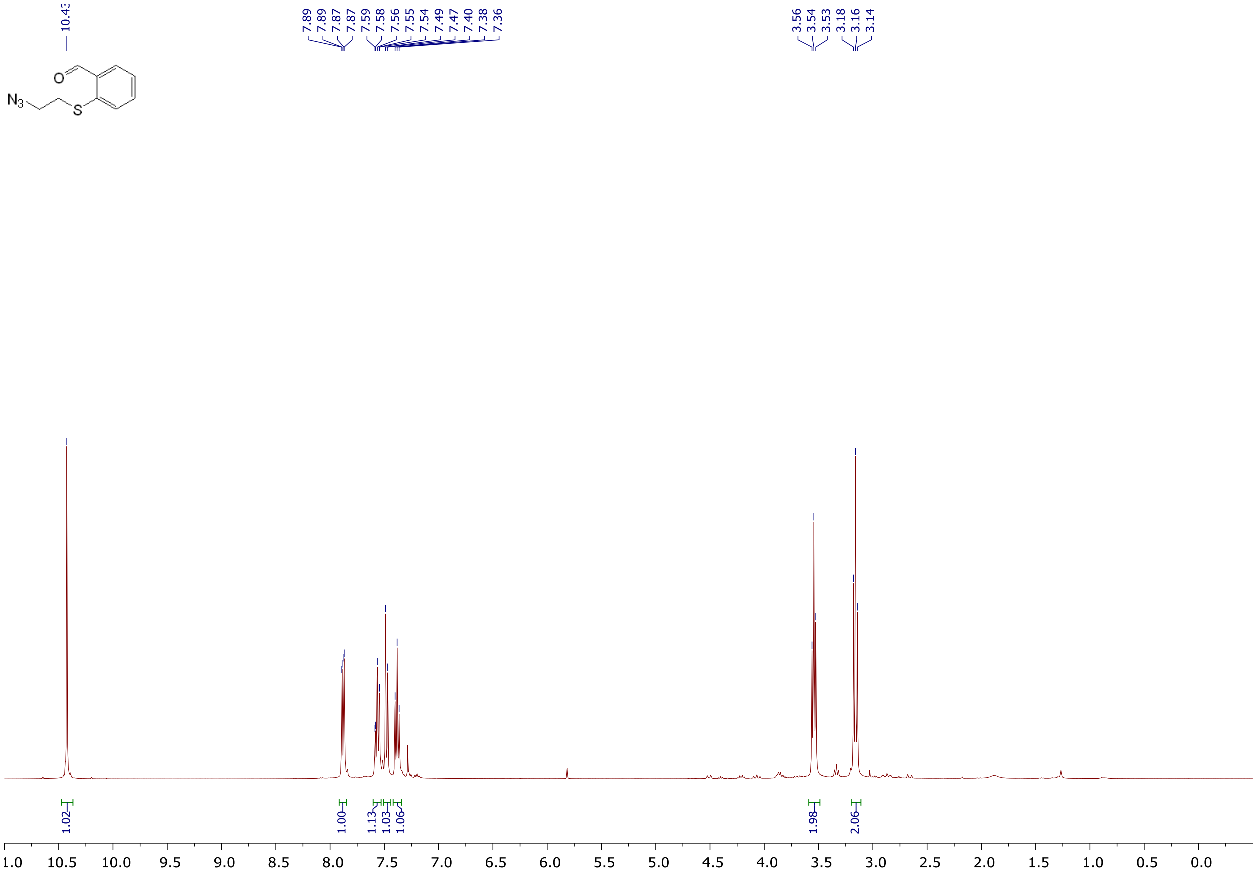

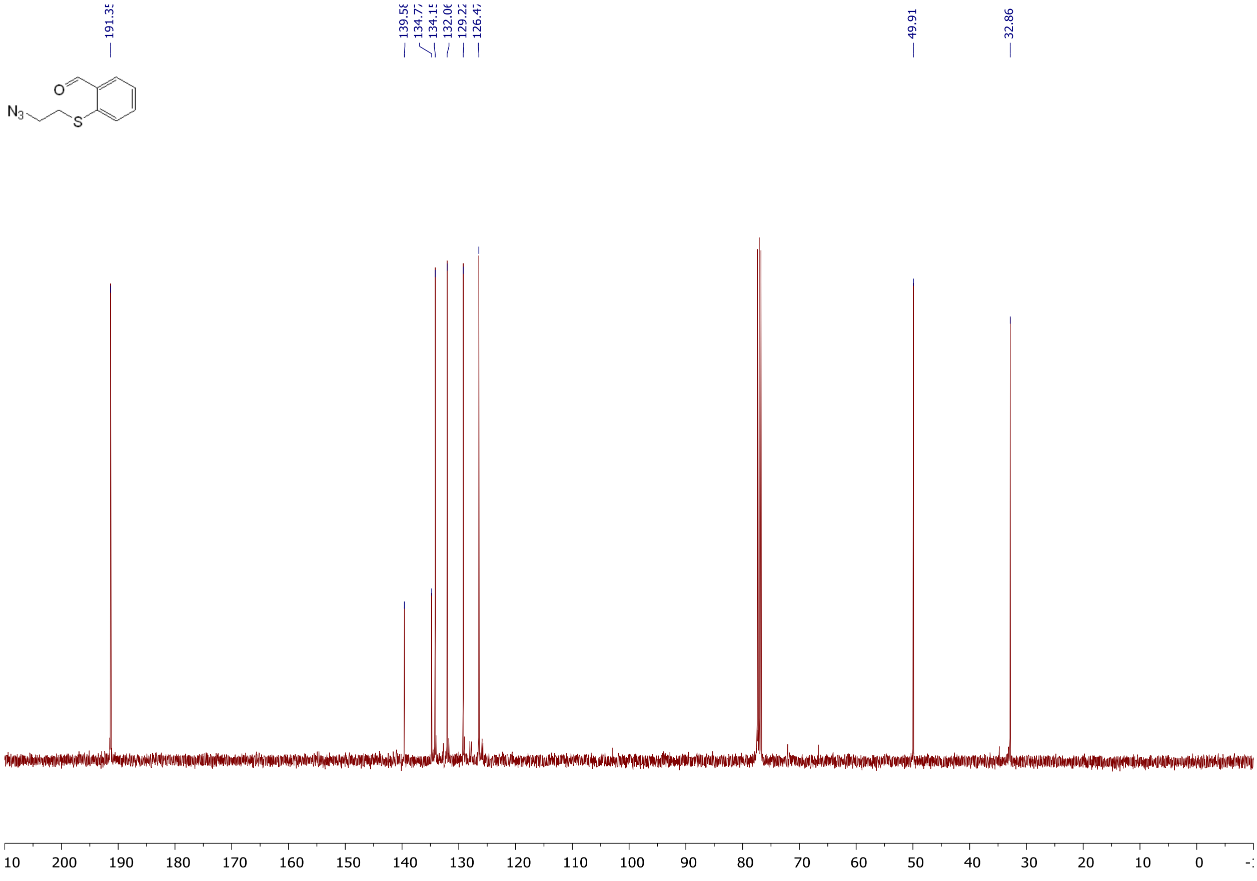

Copies of ^{1}H 400.13 MHz, $CDCl_3$ and ^{13}C 100.61 MHz, $CDCl_3$ spectra of **1m**

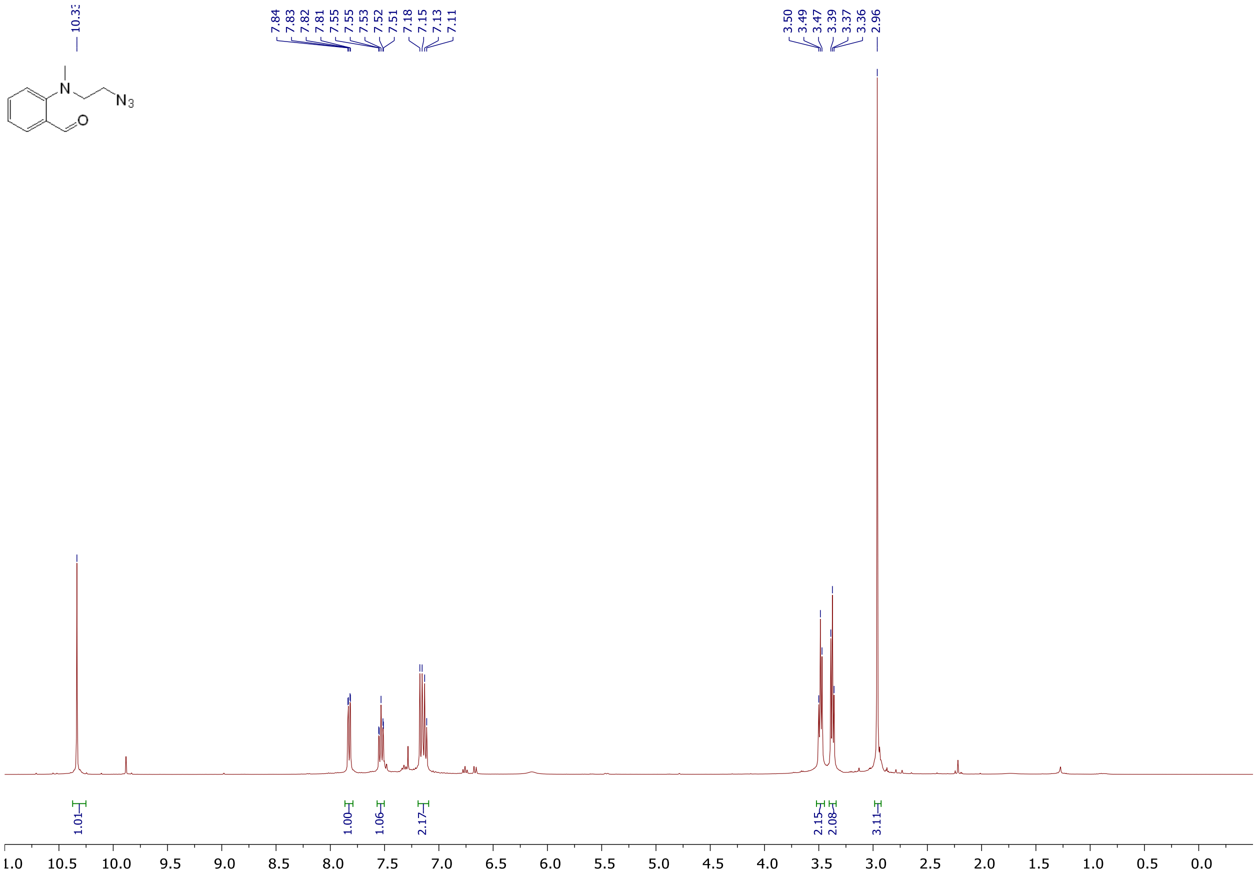

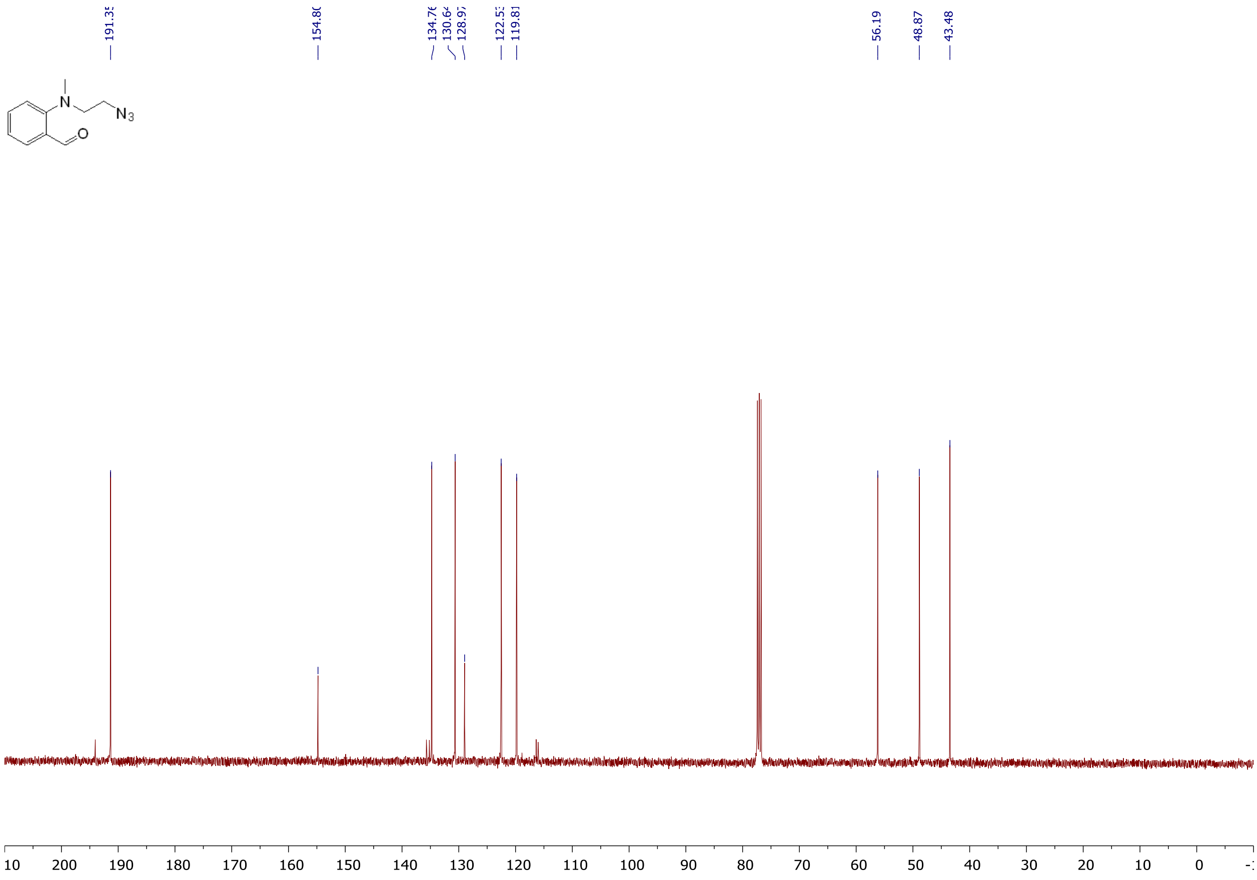

Copies of ^{1}H 400.13 MHz, DMSO-d_6 and ^{13}C 100.61 MHz, DMSO-d_6 spectra of **6a**

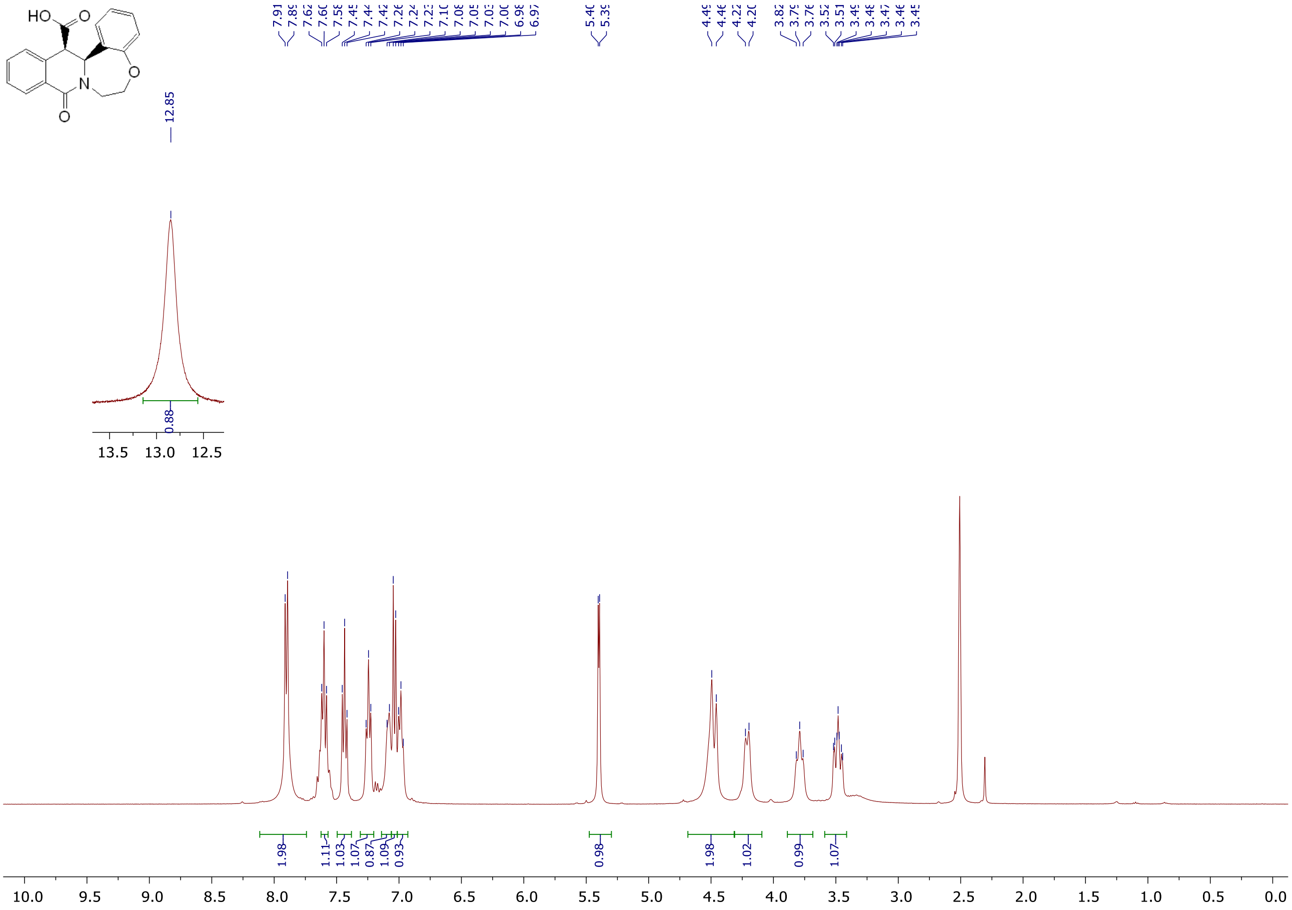

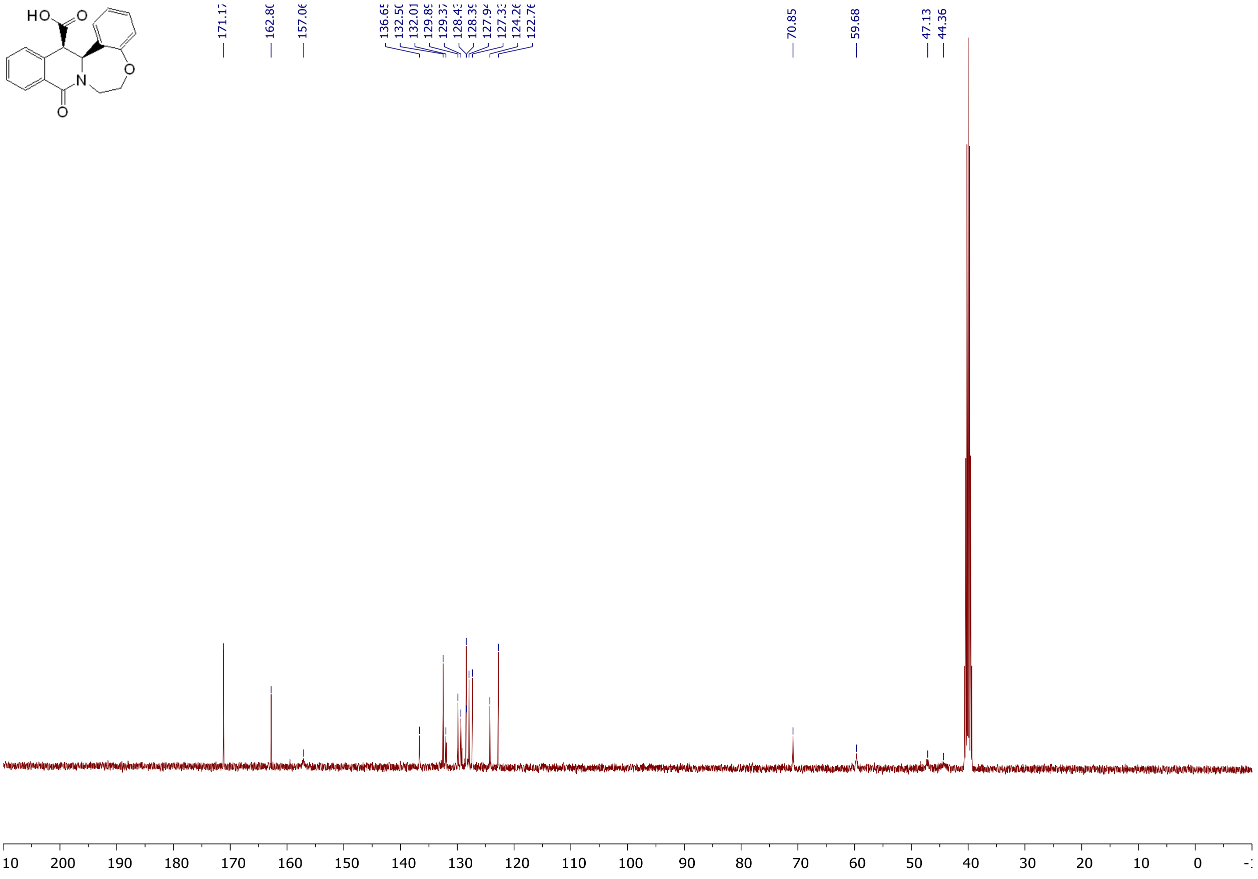

Copies of ^{1}H 400.13 MHz, DMSO-d_6 and ^{13}C 100.61 MHz, DMSO-d_6 spectra of **6a’**

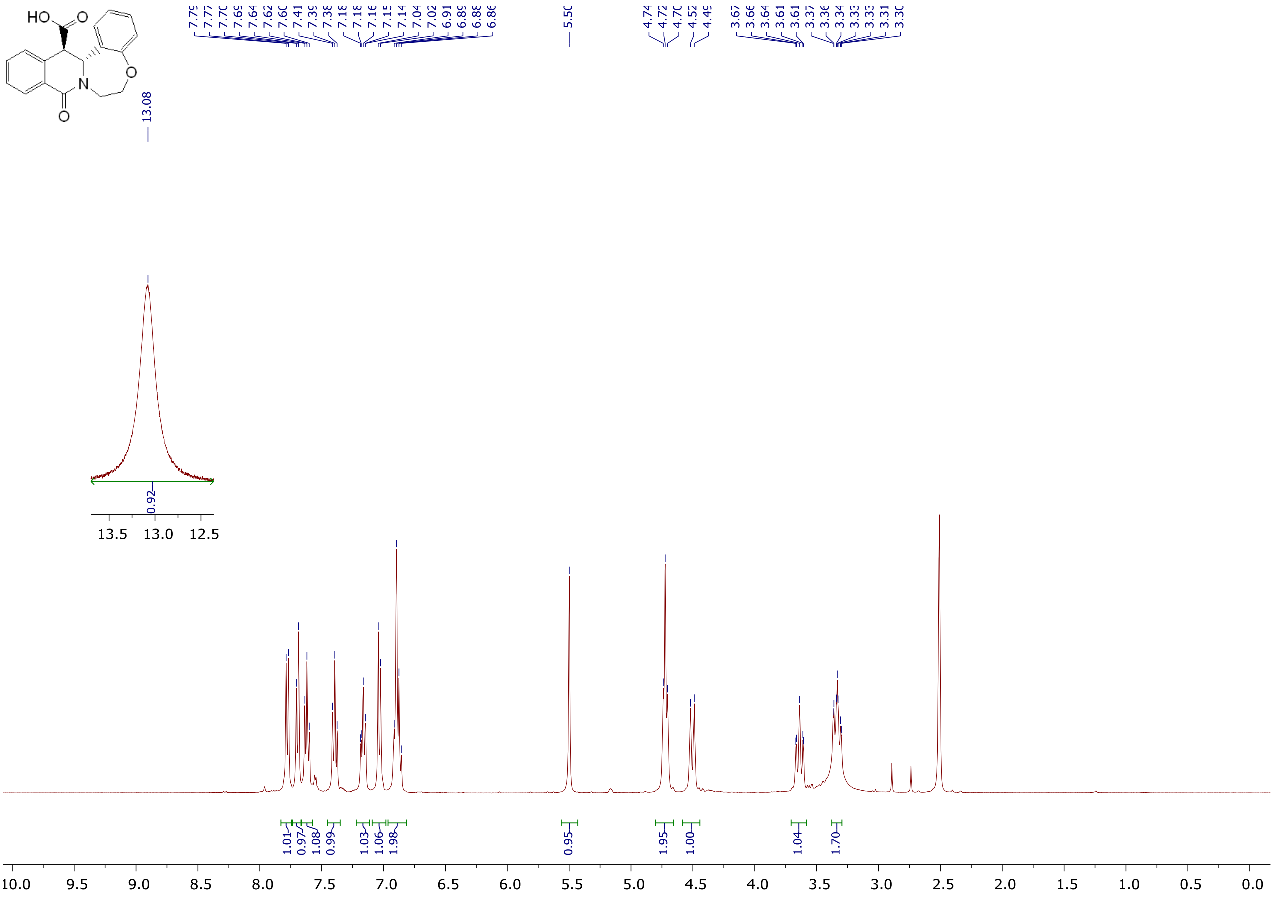

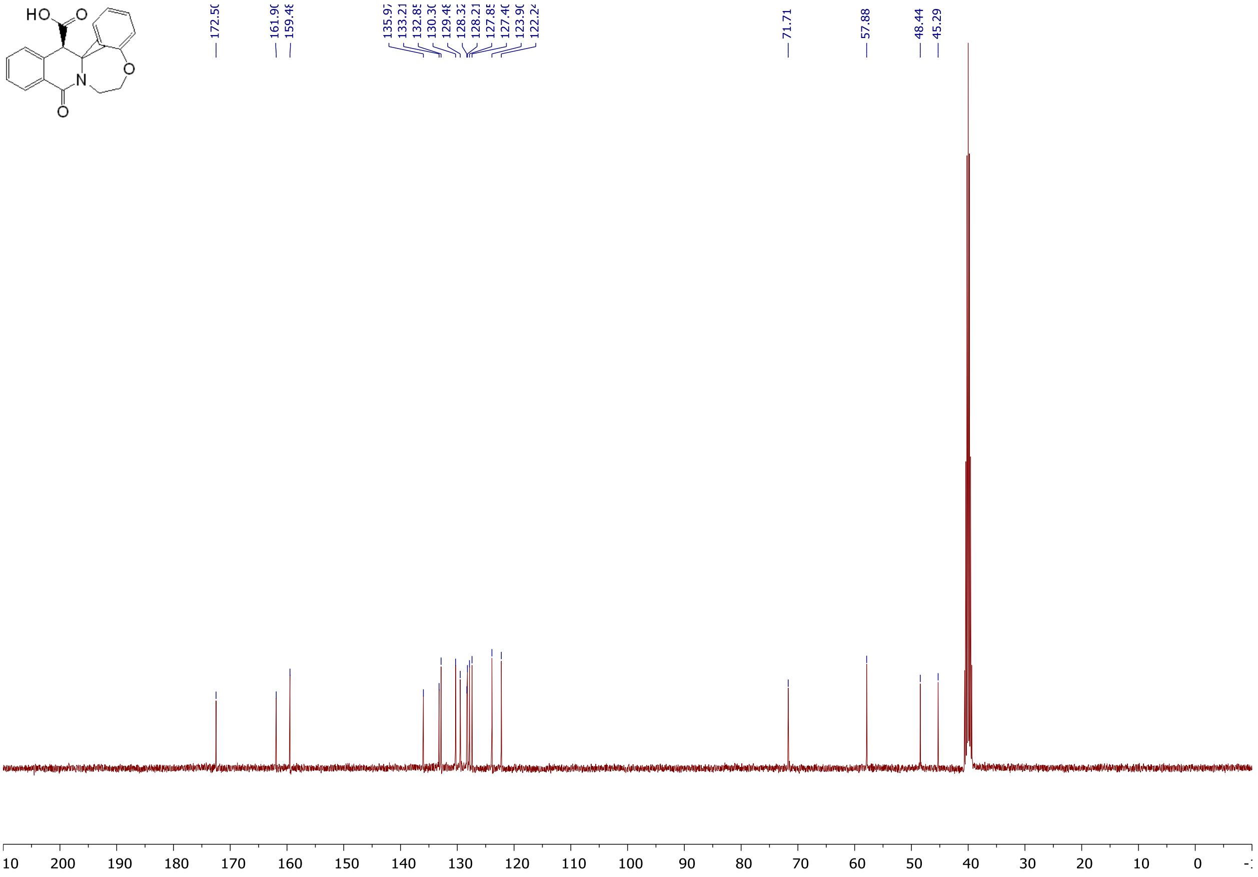

Copies of ^{1}H 400.13 MHz, $CDCl_3$, ^{13}C 100.61 MHz, $CDCl_3$ and ^{19}F 376.50 MHz, $CDCl_3$ spectra of **6b**

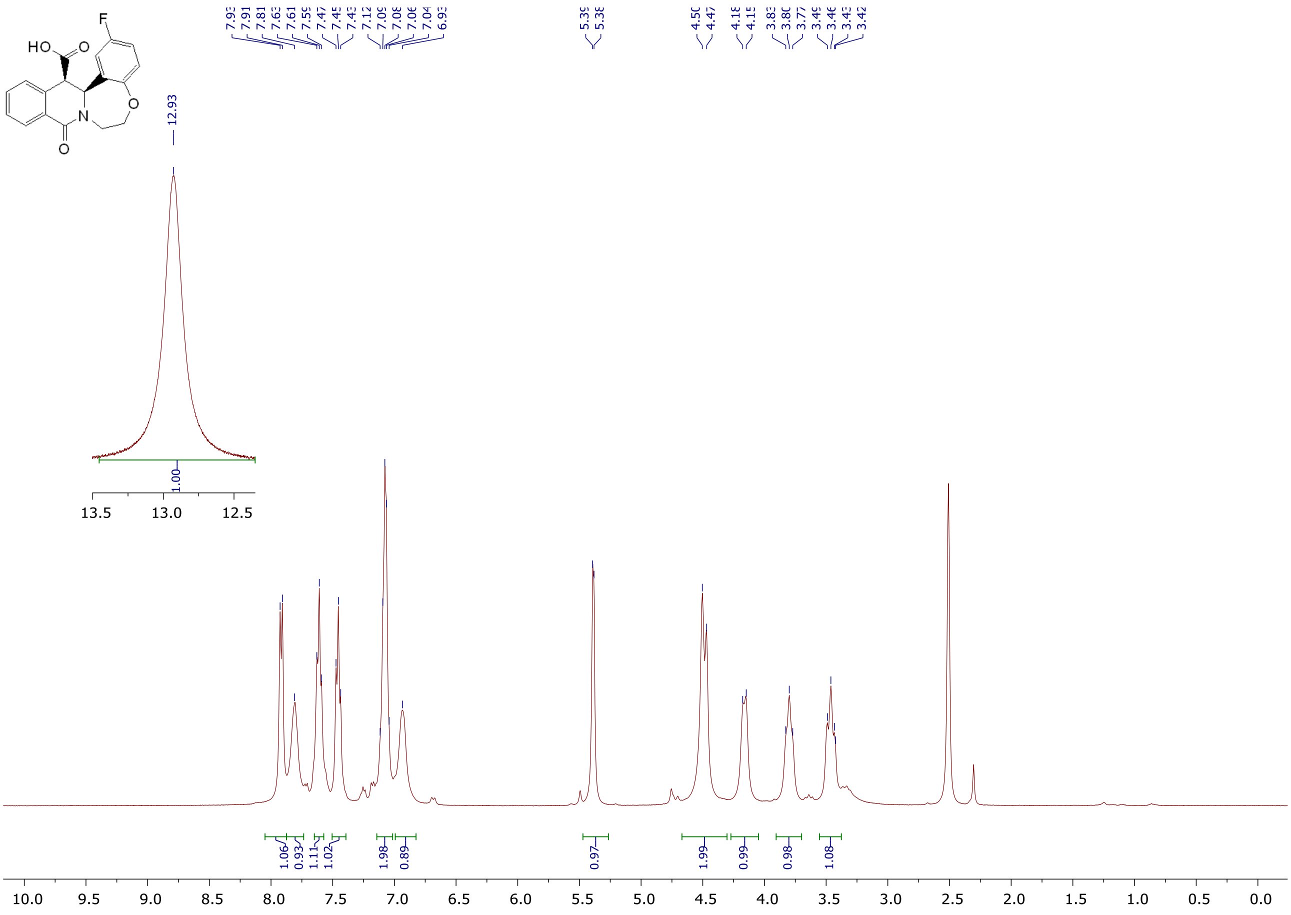

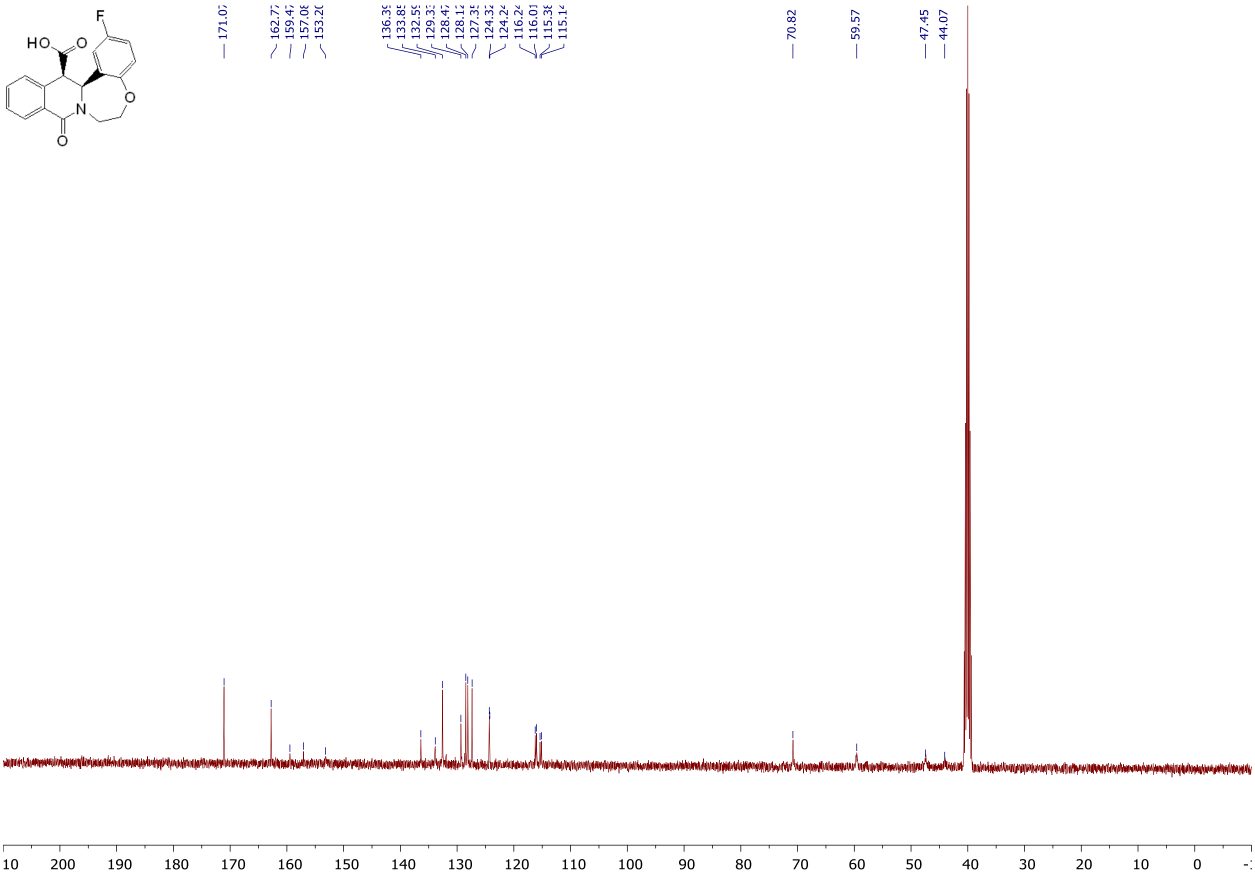

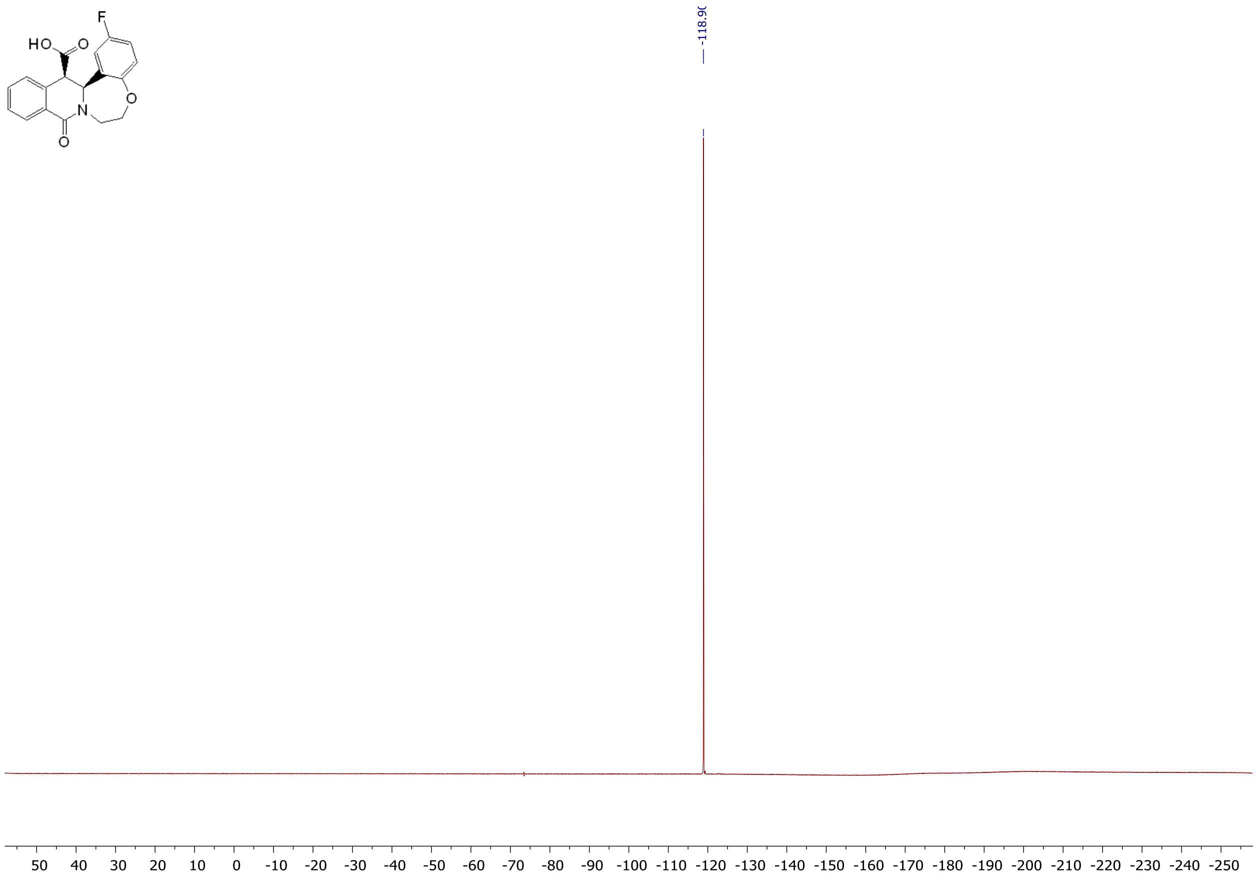
F
HO
O
N
O
O
-118.9
50 40 30 20 10 0 -10 -20 -30 -40 -50 -60 -70 -80 -90 -100 -110 -120 -130 -140 -150 -160 -170 -180 -190 -200 -210 -220 -230 -240 -250

Copies of ^{1}H 400.13 MHz, DMSO-d_6 and ^{13}C 100.61 MHz, DMSO-d_6 spectra of **6c**

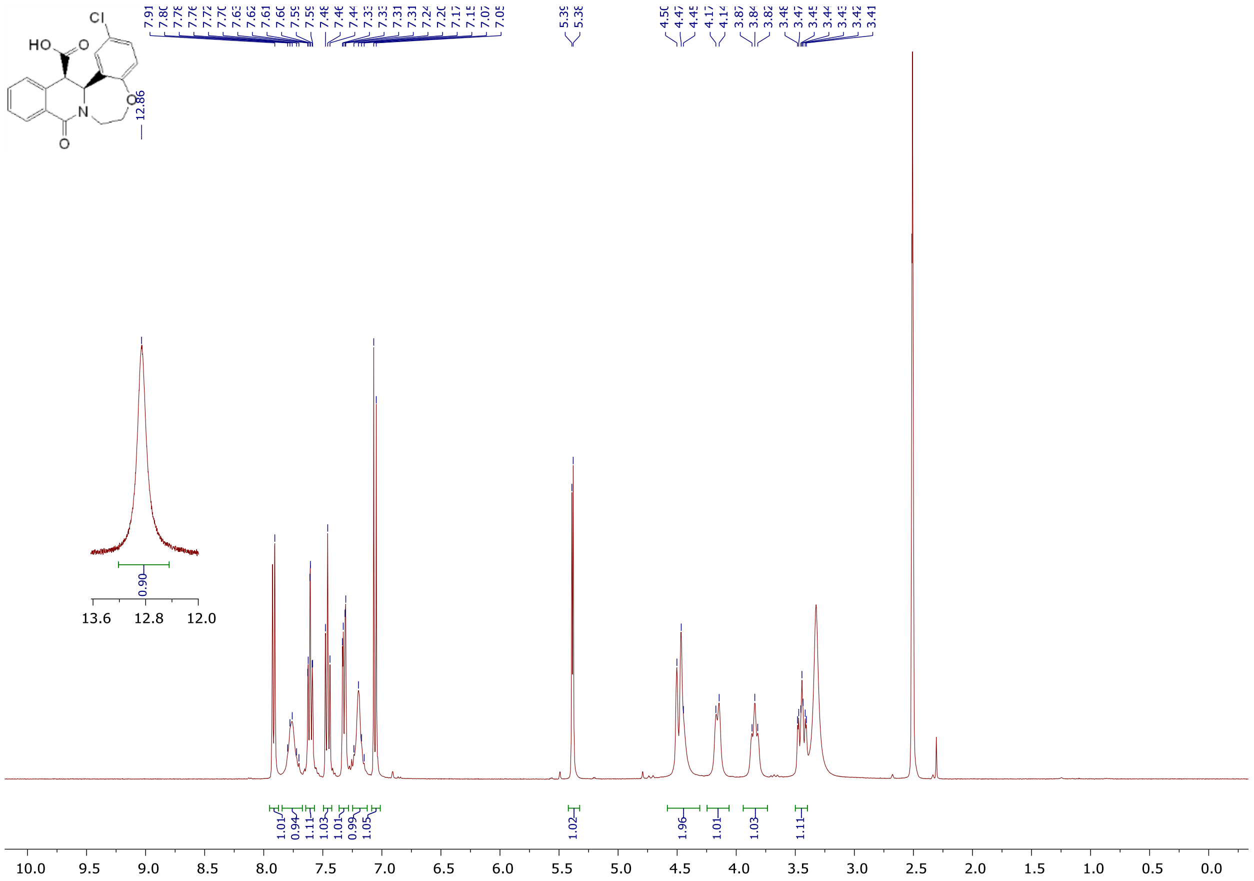

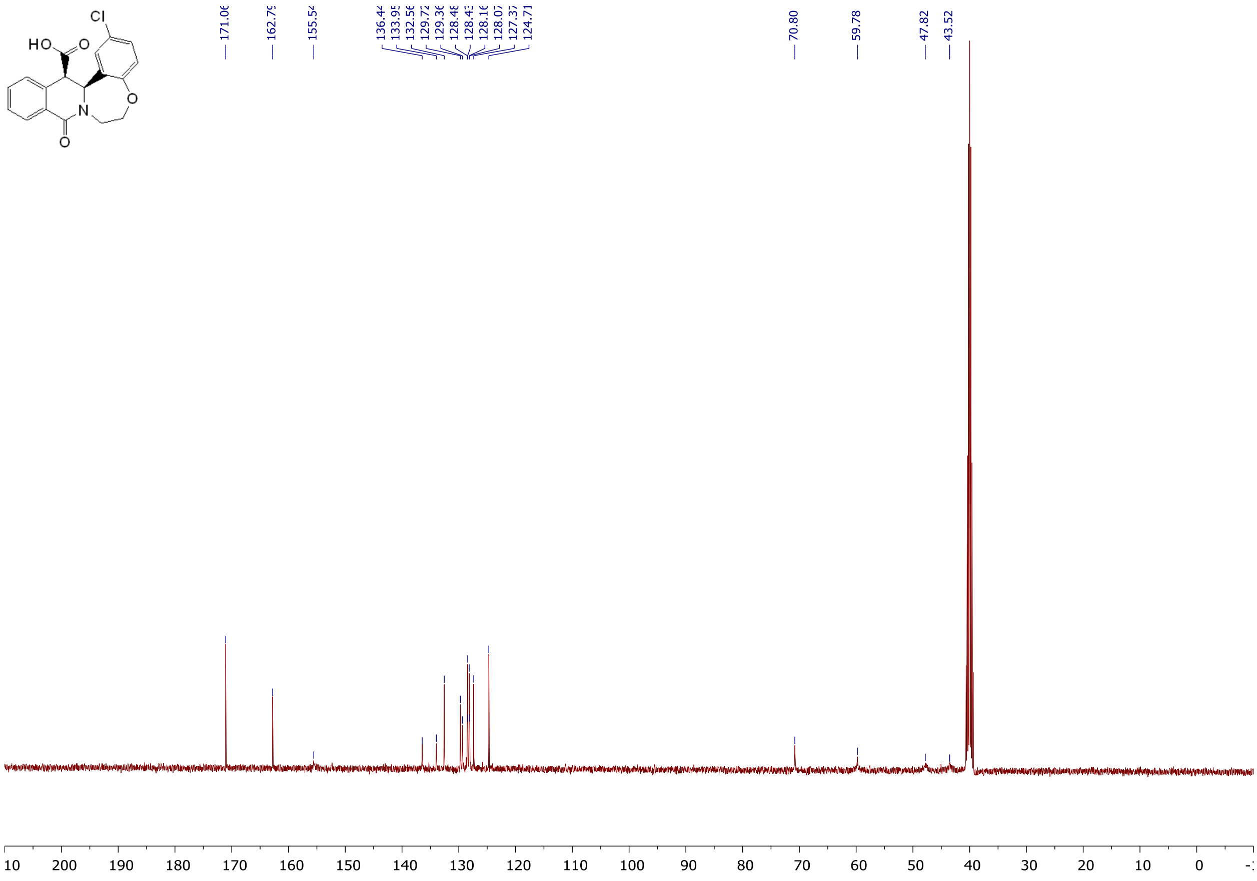

Copies of ^{1}H 400.13 MHz, DMSO-d_6 and ^{13}C 100.61 MHz, DMSO-d_6 spectra of **6d**

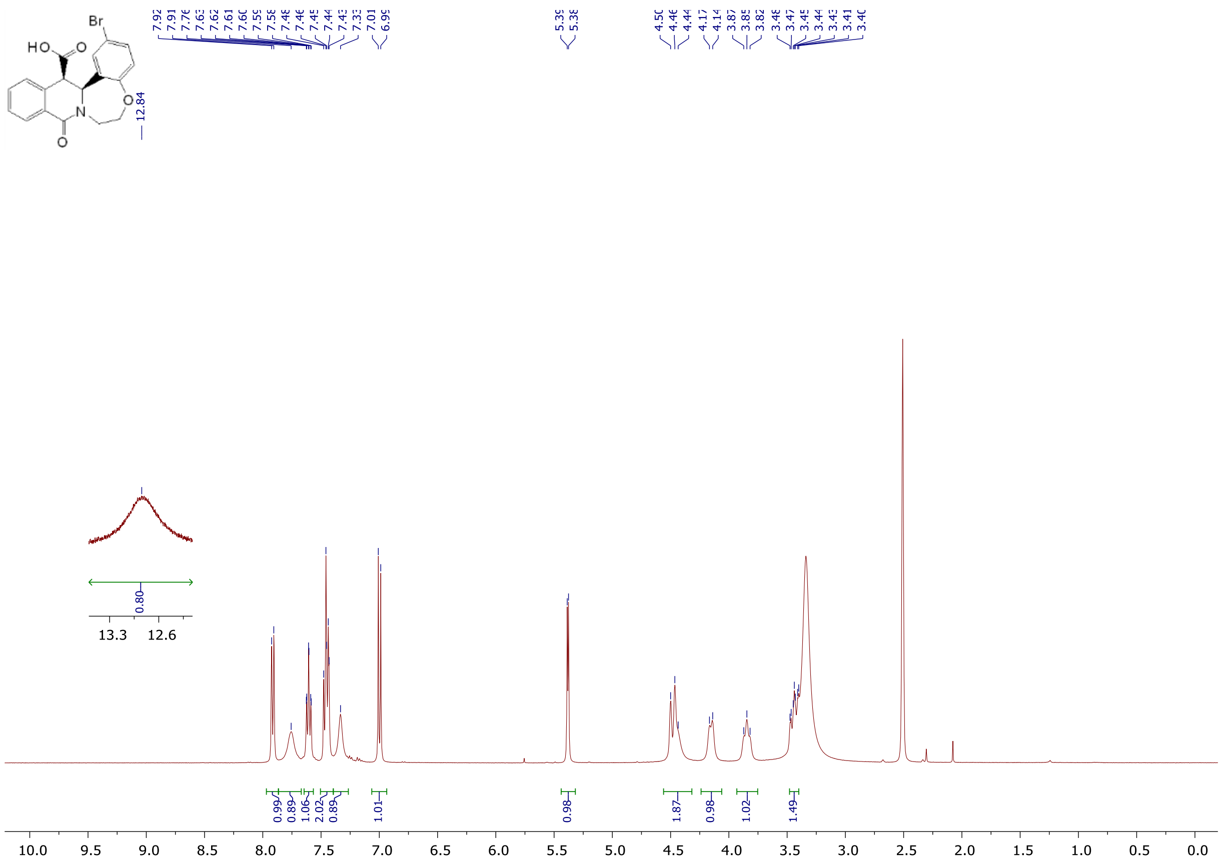

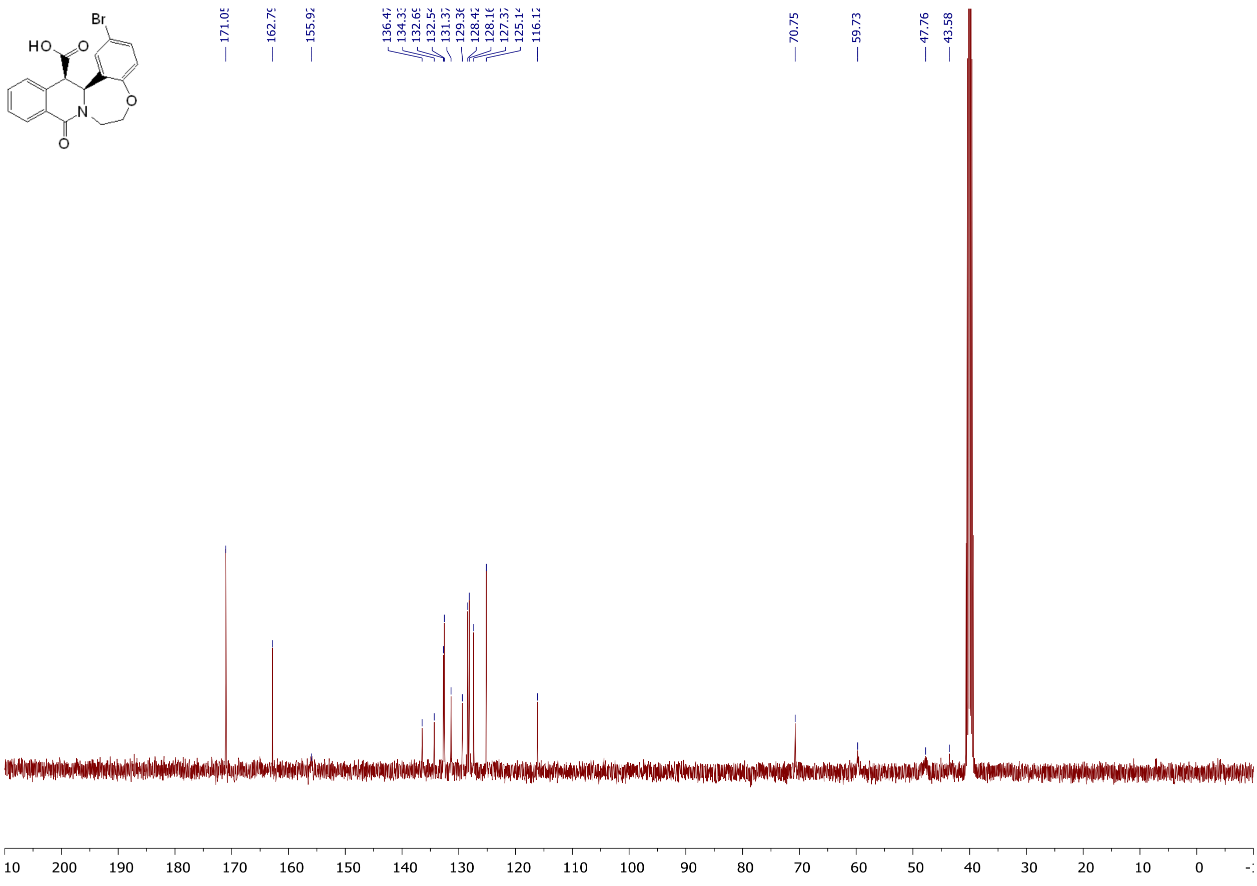

Copies of ^{1}H 400.13 MHz, DMSO-d_6 and ^{13}C 100.61 MHz, DMSO-d_6 spectra of **6e**

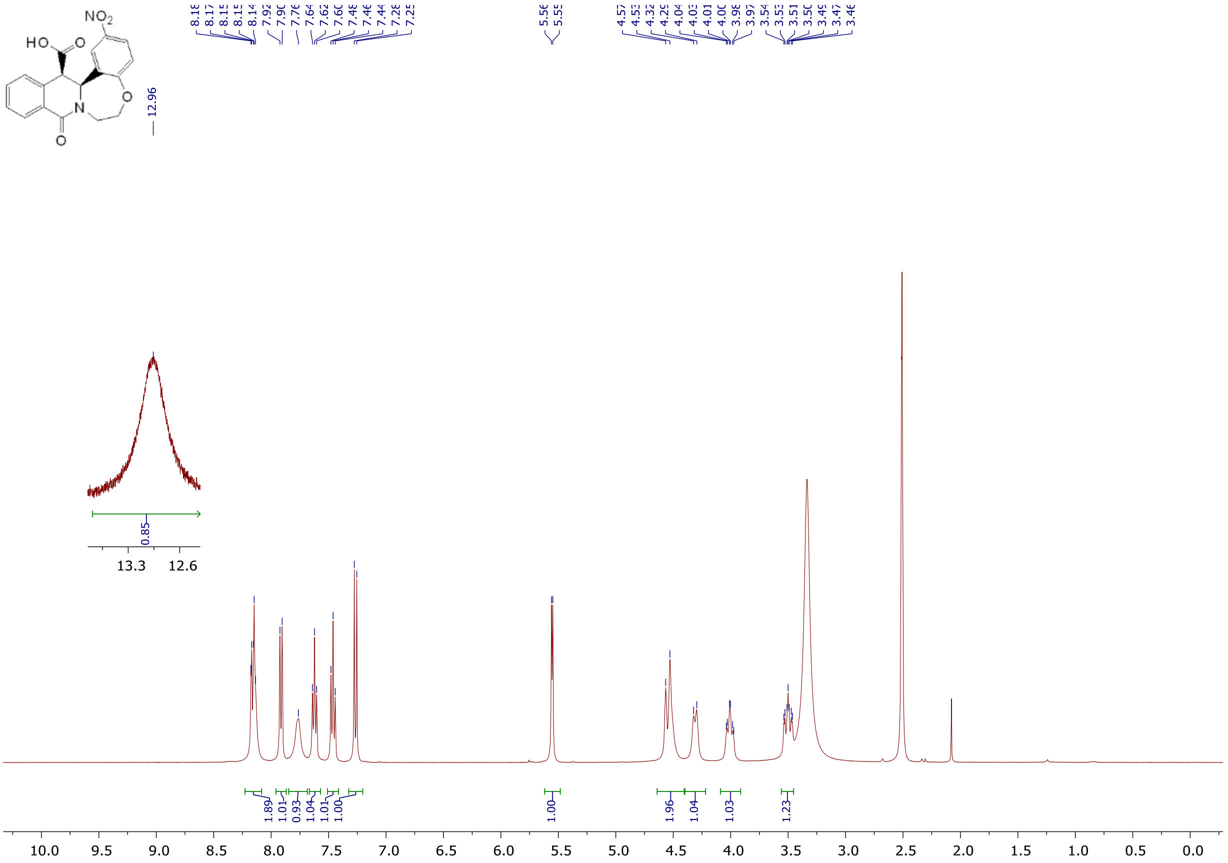

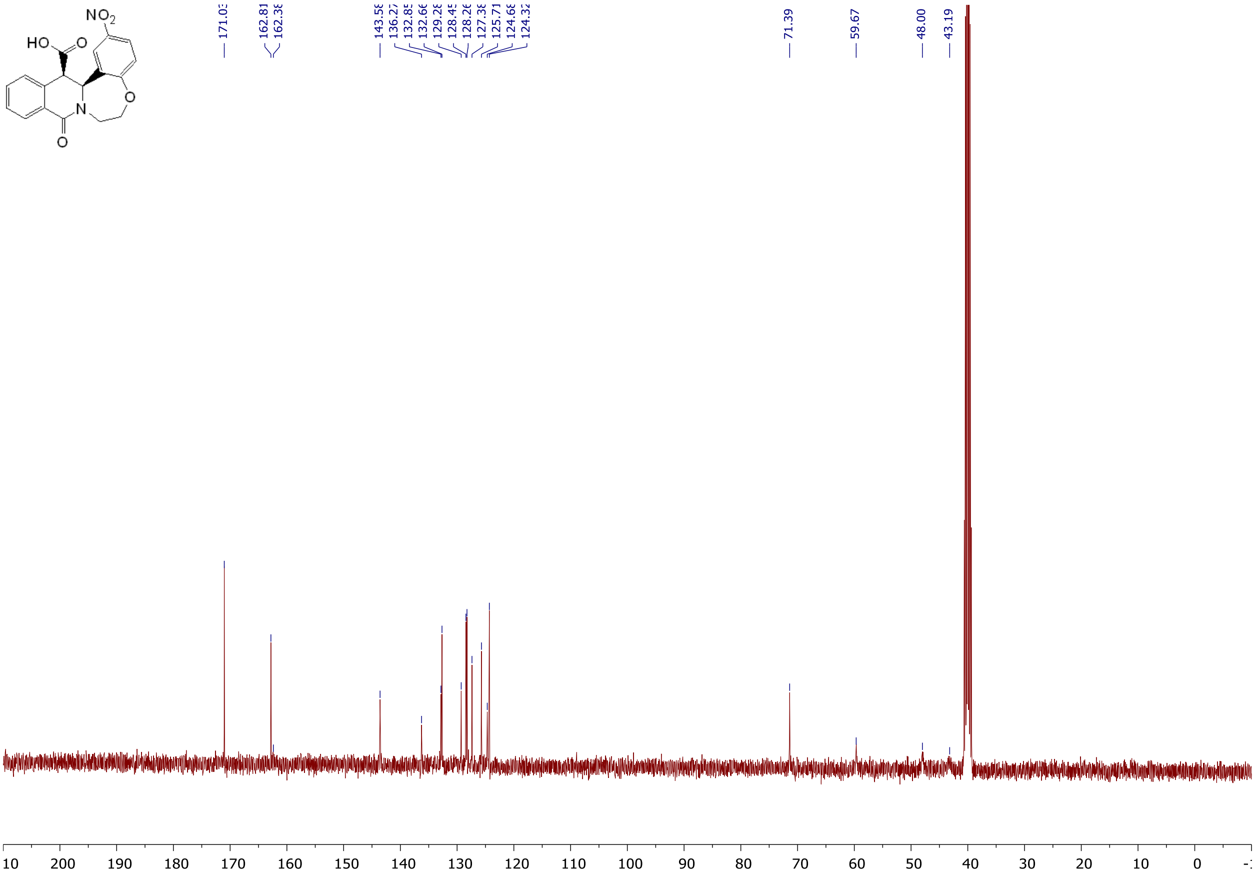

Copies of ^{1}H 400.13 MHz, DMSO-d_6 and ^{13}C 100.61 MHz, DMSO-d_6 spectra of **6f**

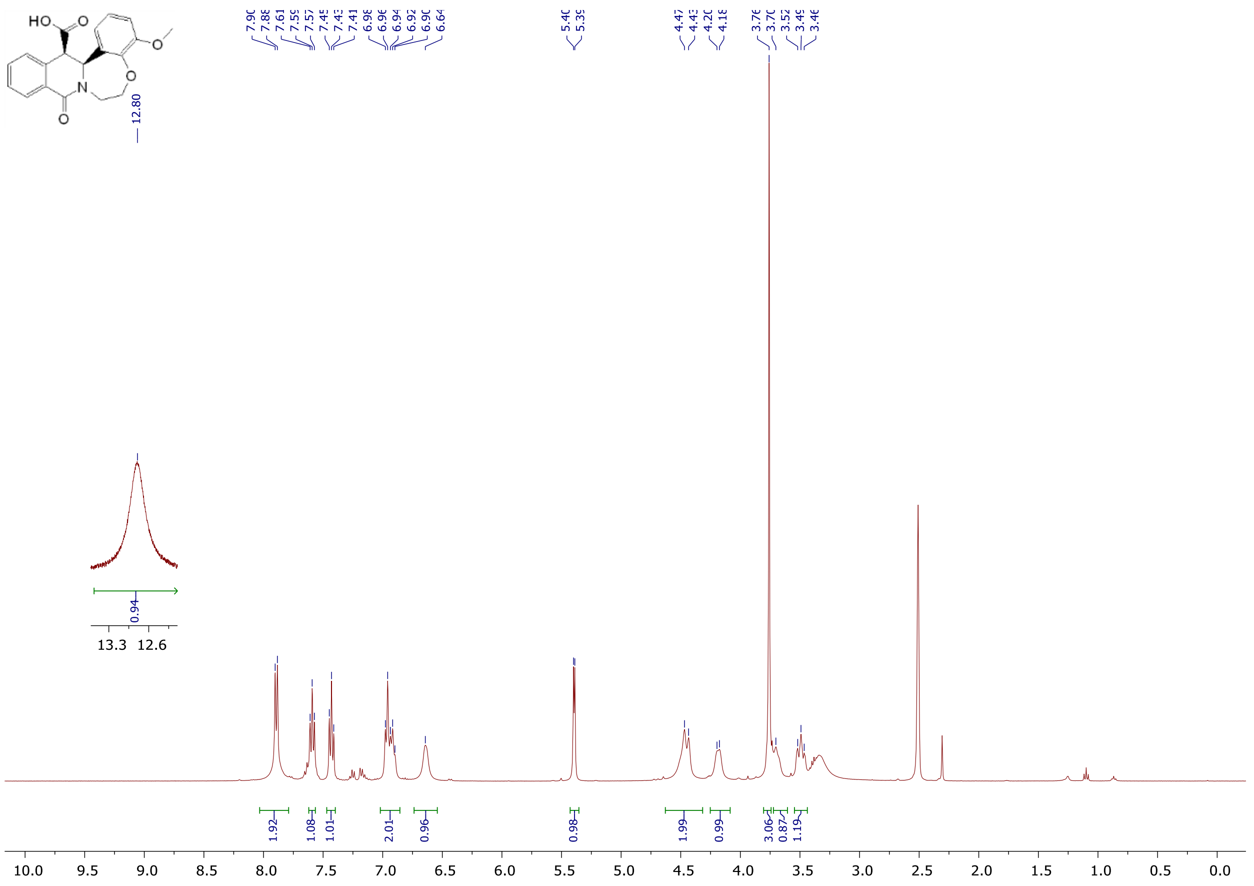

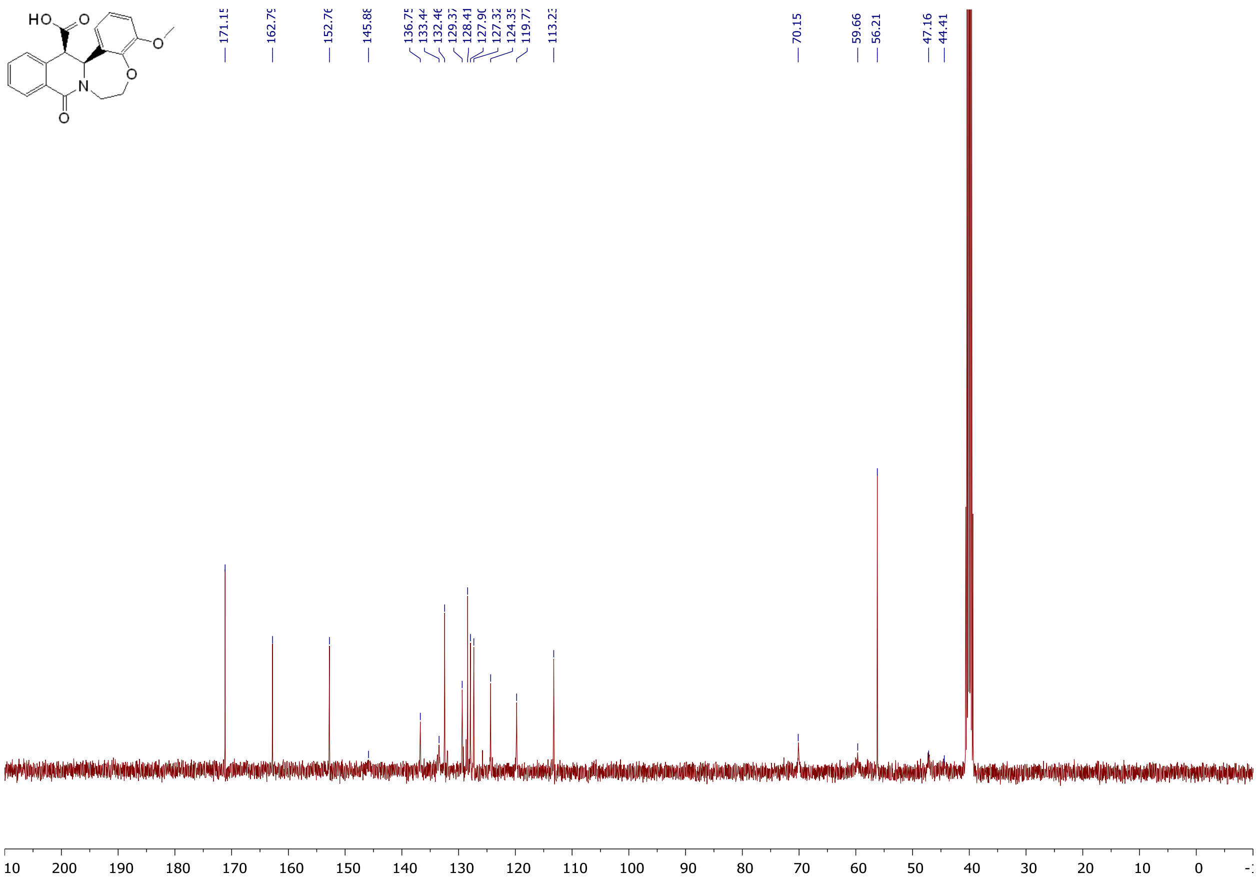

Copies of ^{1}H 400.13 MHz, DMSO-d_6 and ^{13}C 100.61 MHz, DMSO-d_6 spectra of **6g**

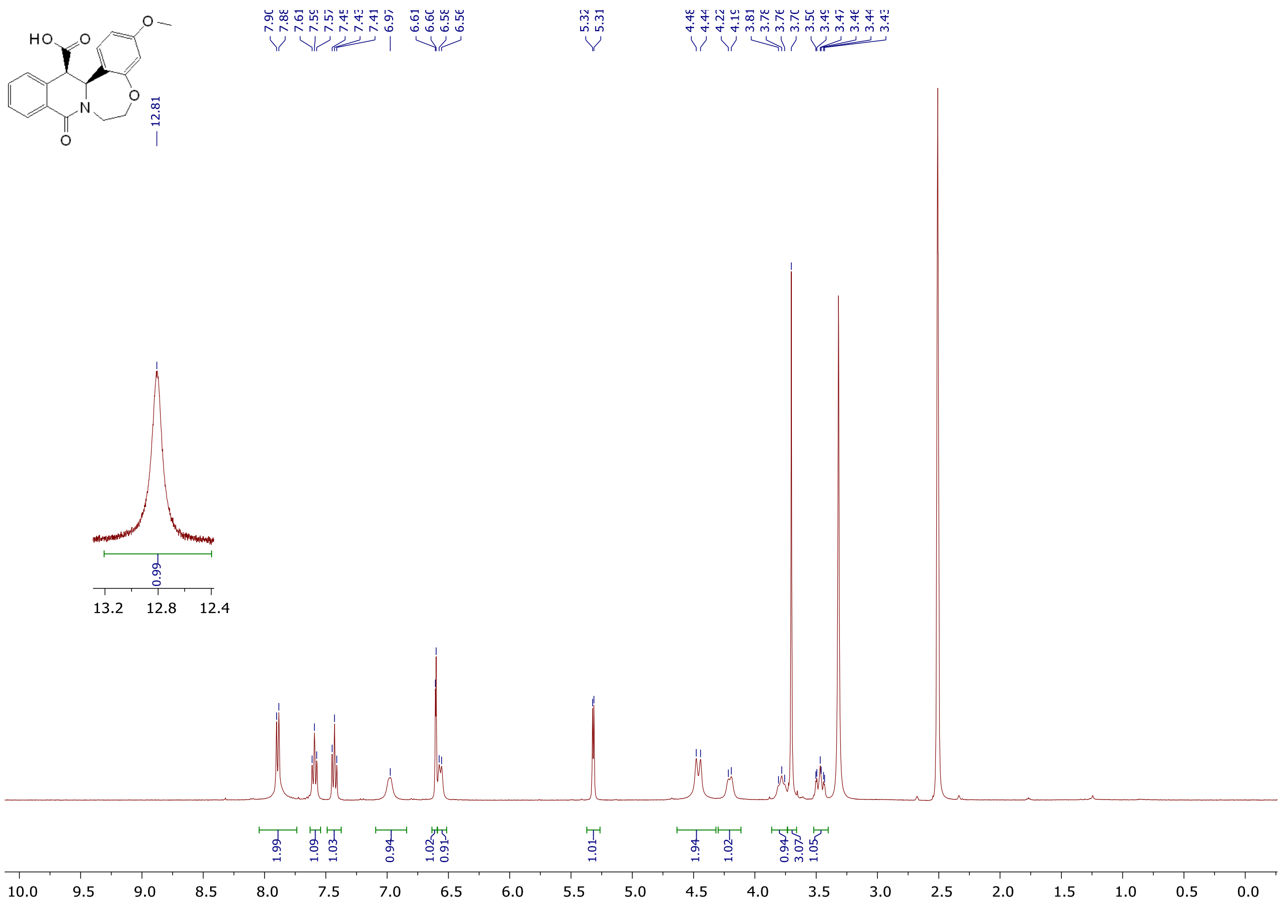

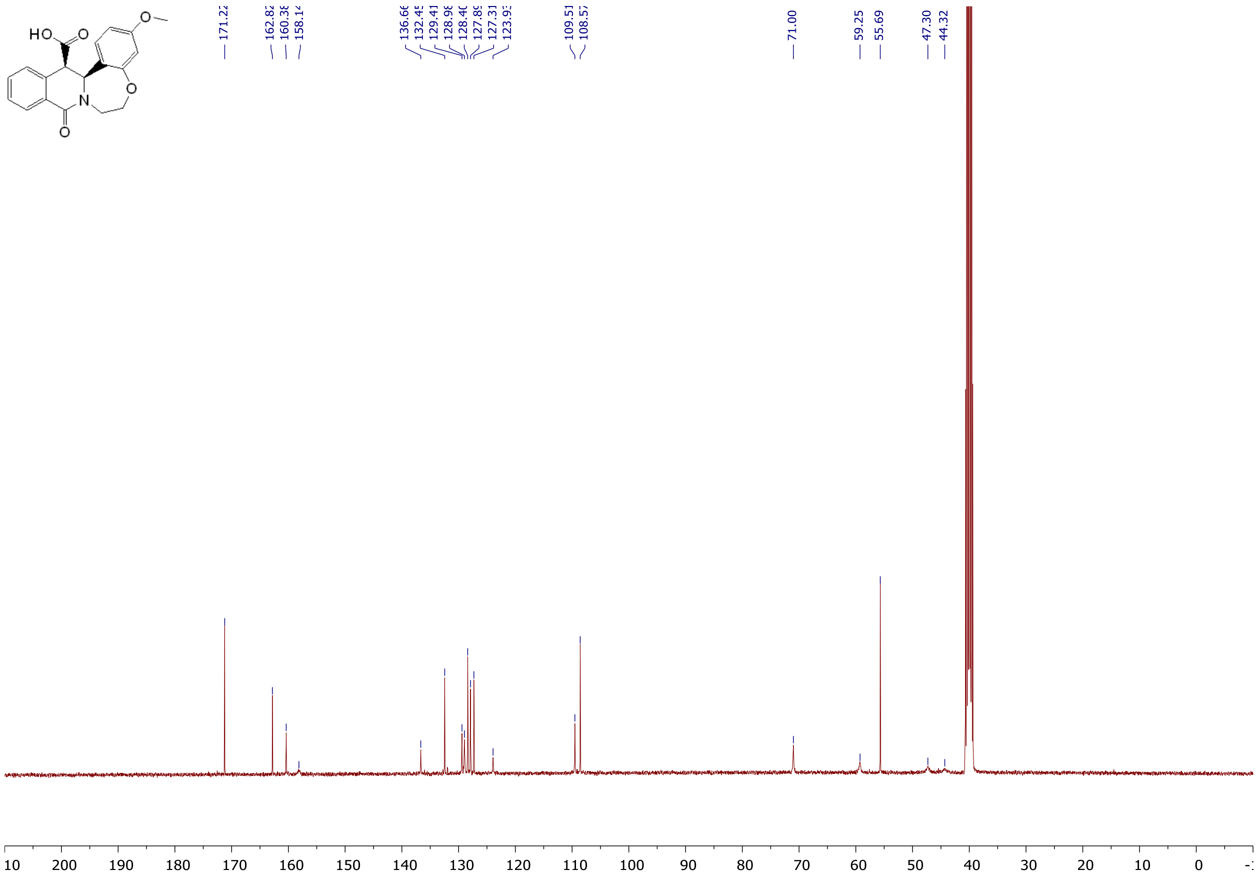

Copies of ^{1}H 400.13 MHz, DMSO-d_6 and ^{13}C 100.61 MHz, DMSO-d_6 spectra of **6h**

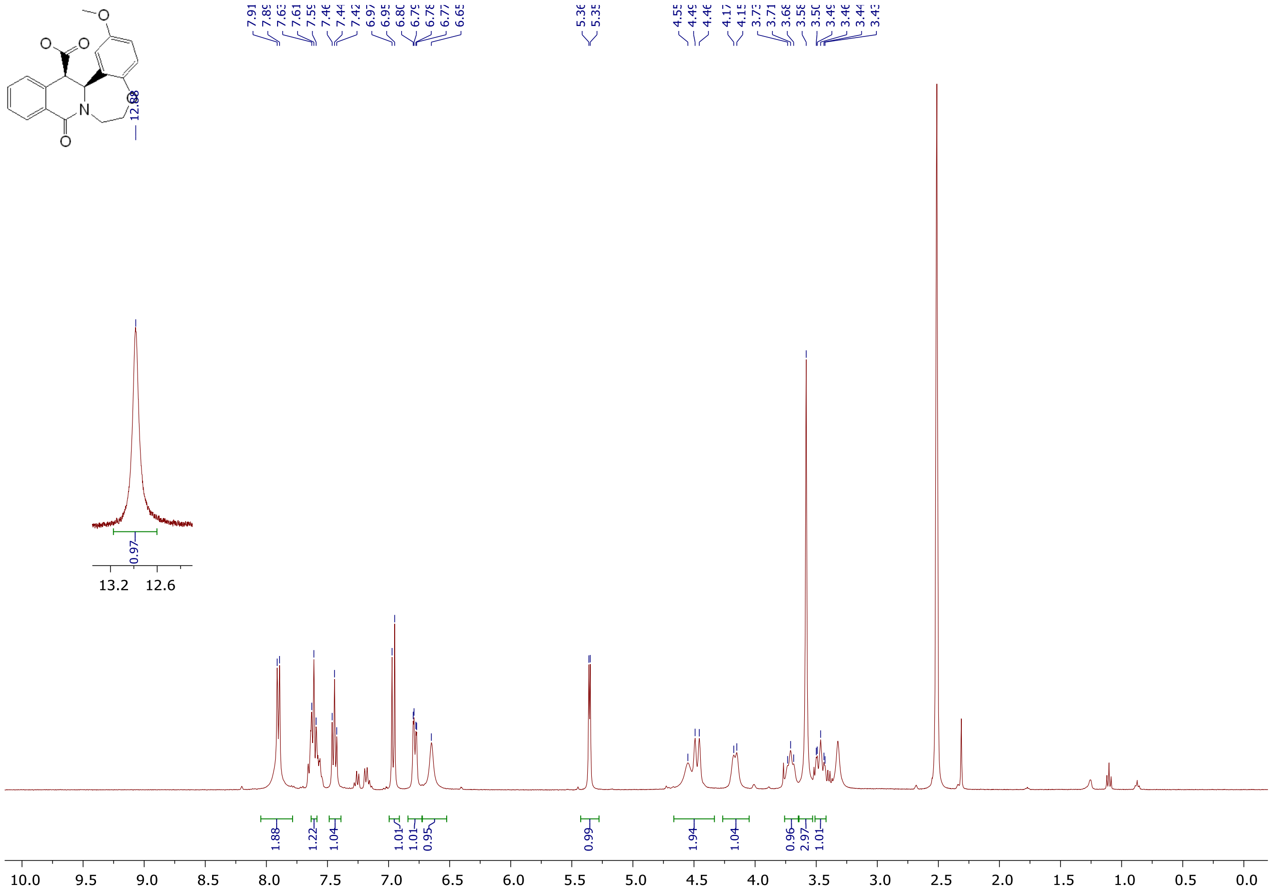

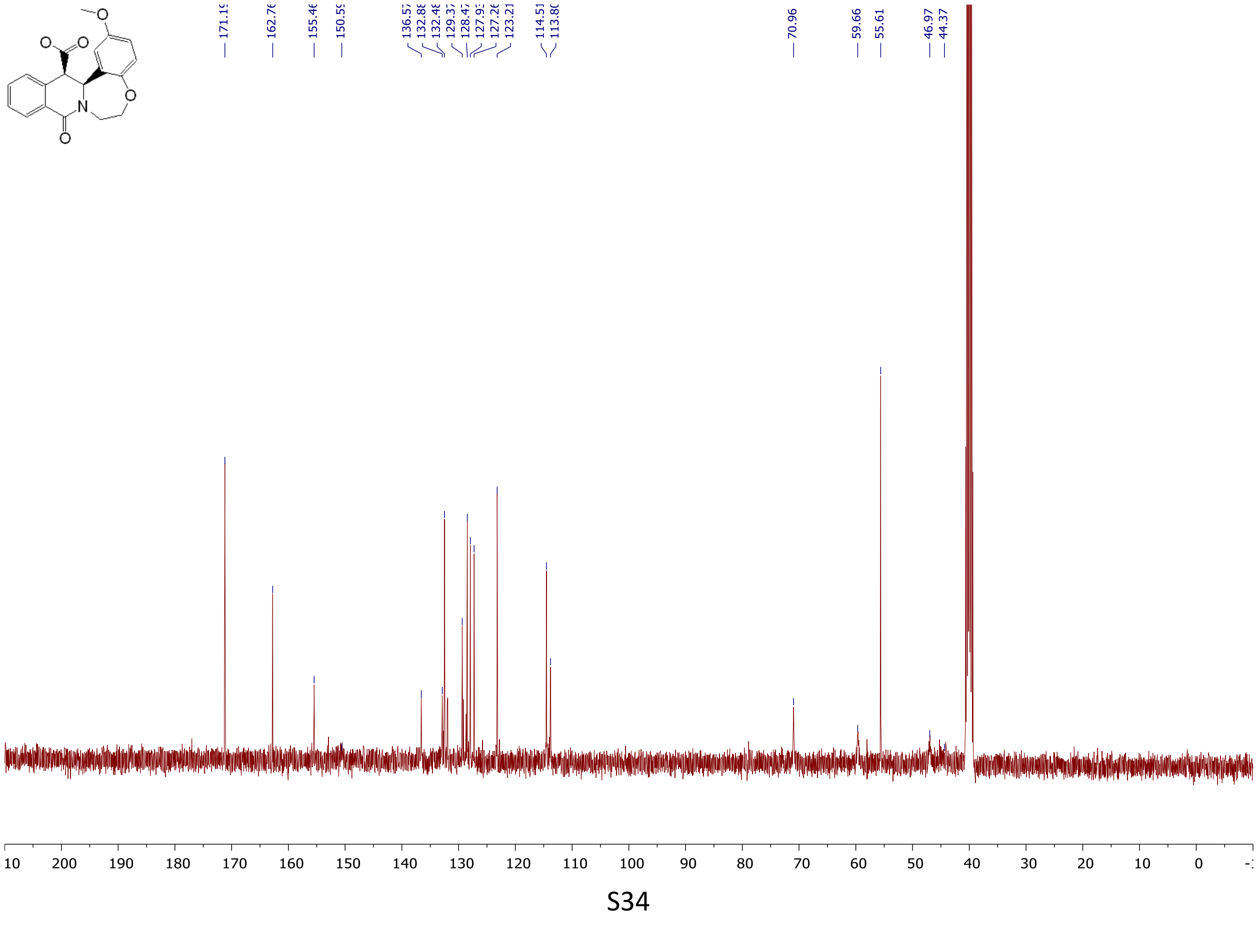

Copies of ^{1}H 400.13 MHz, DMSO-d_6 and ^{13}C 100.61 MHz, DMSO-d_6 spectra of **6i**

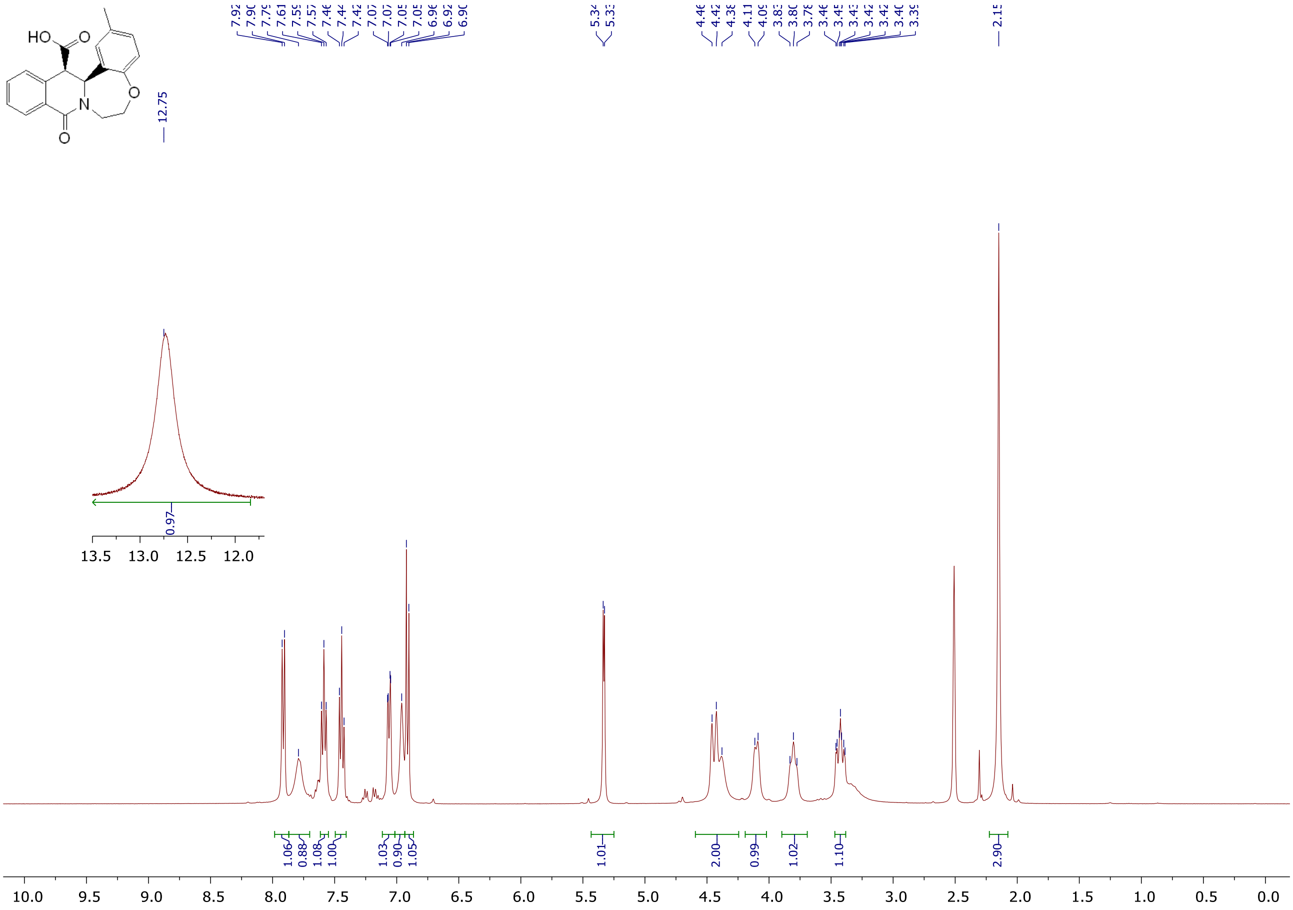

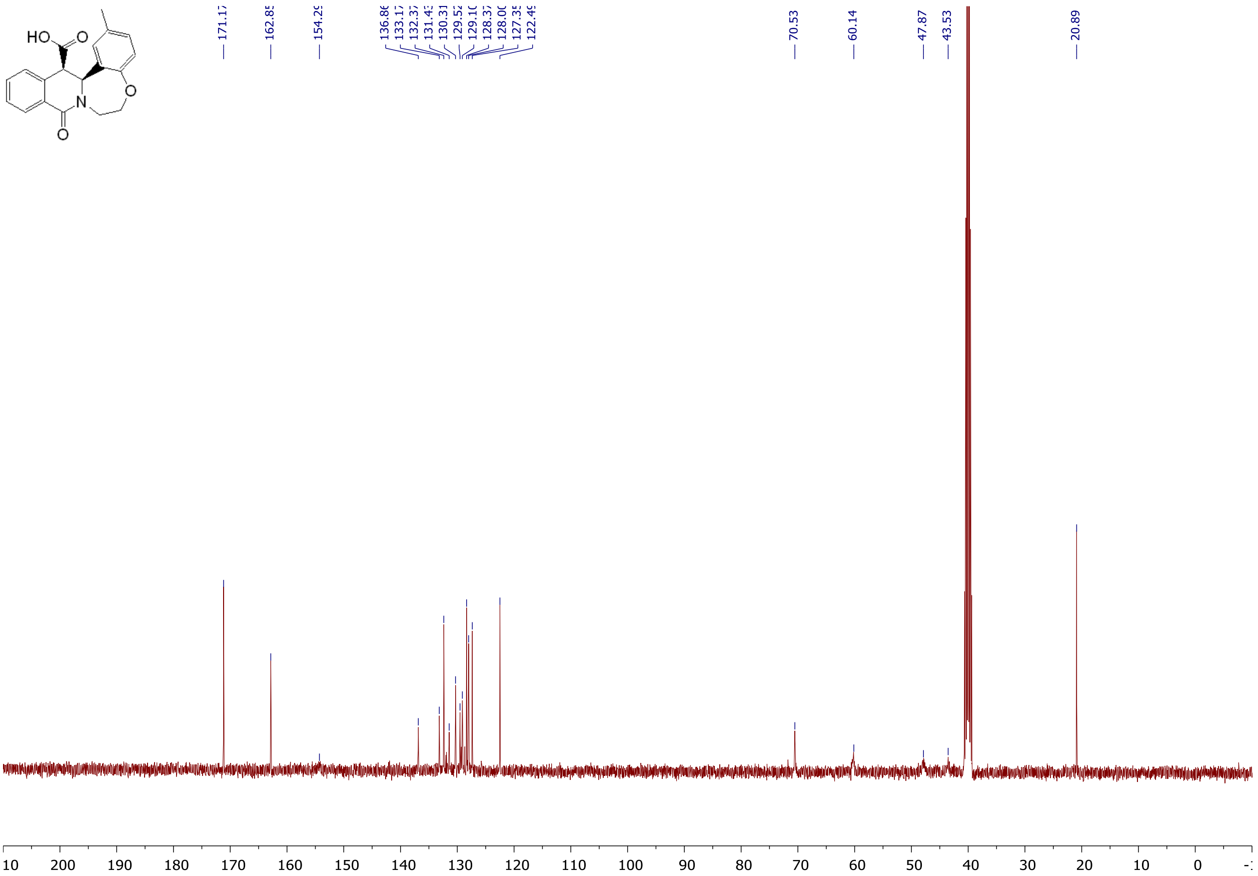

Copies of ^{1}H 400.13 MHz, DMSO-d_6 and ^{13}C 100.61 MHz, DMSO-d_6 spectra of **6j**

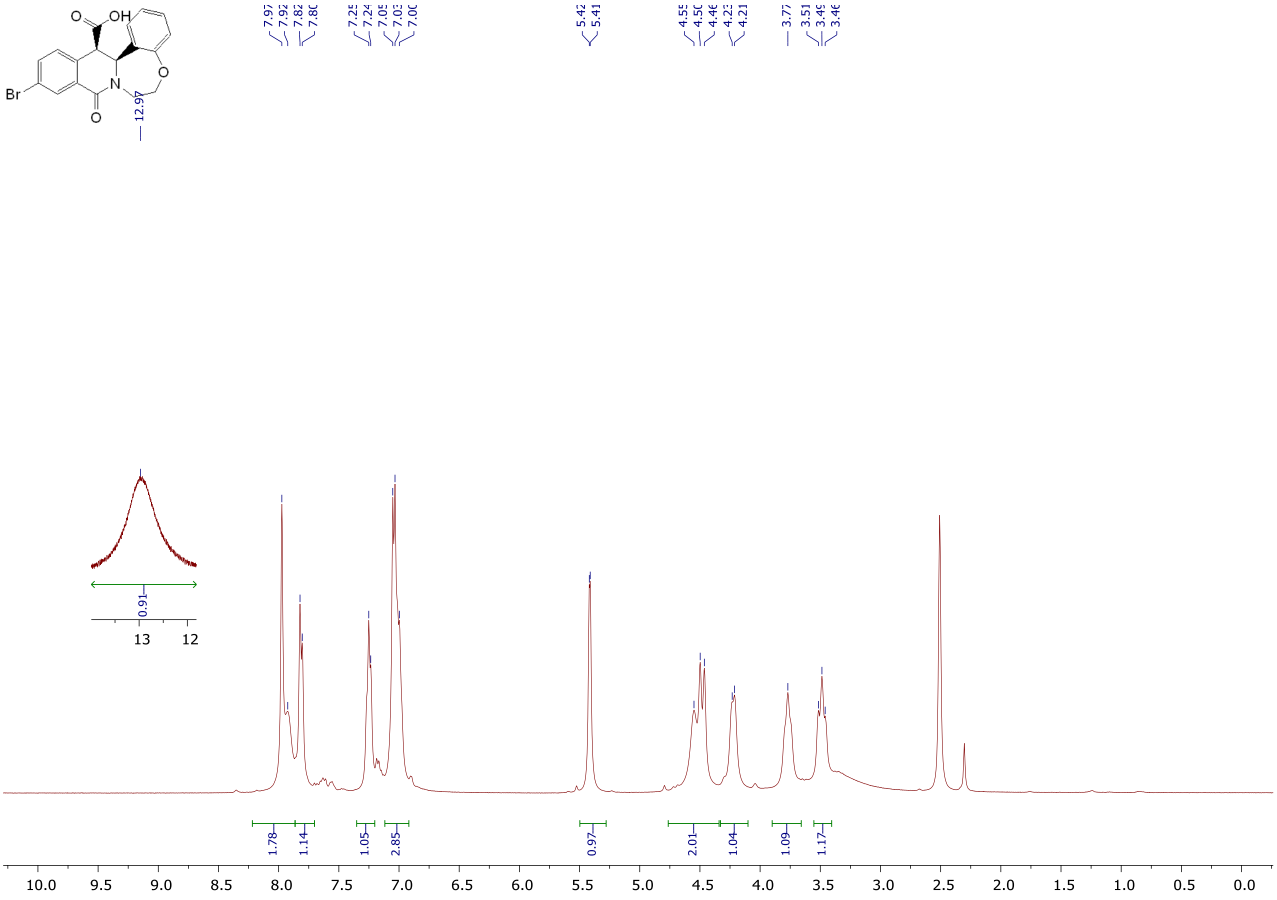

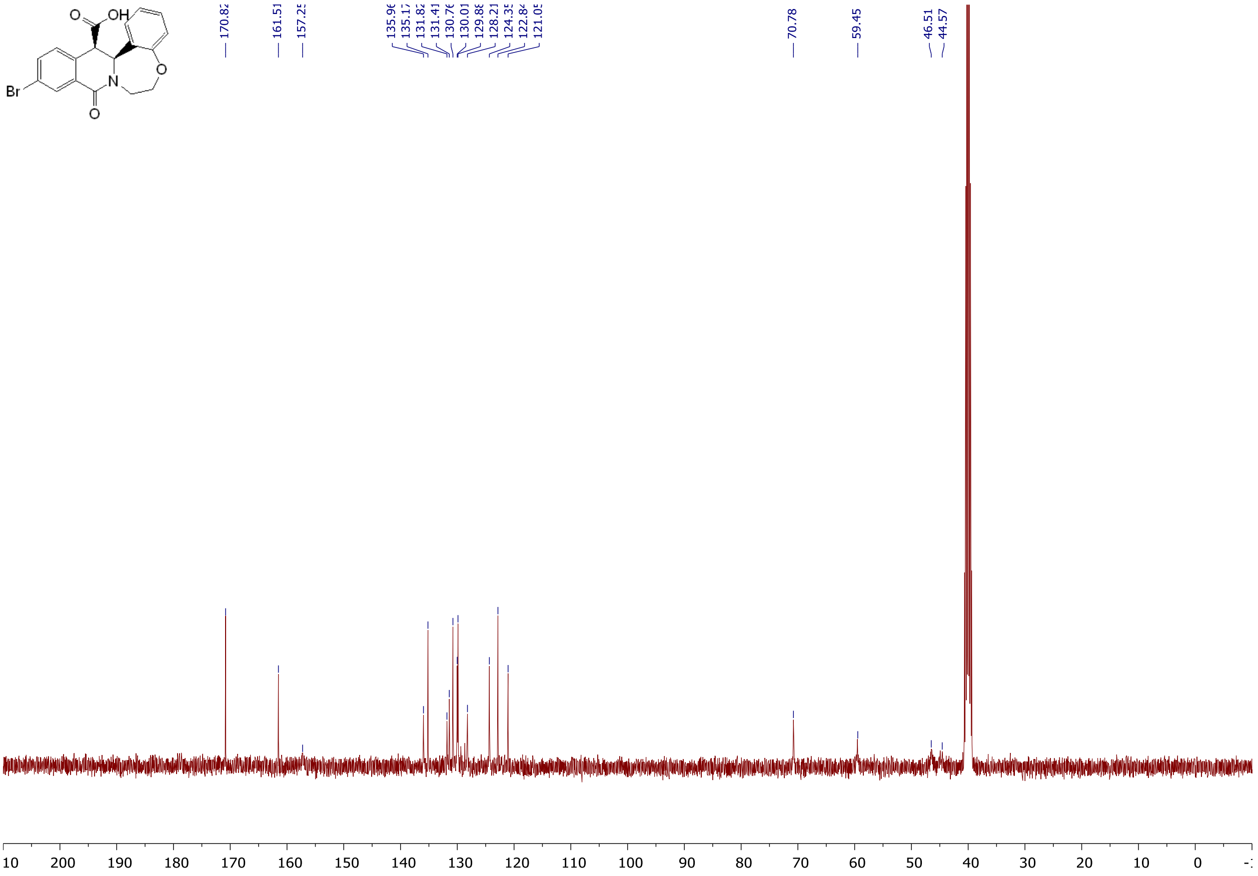

Copies of ^{1}H 400.13 MHz, DMSO-d_6 and ^{13}C 100.61 MHz, DMSO-d_6 spectra of **6k**

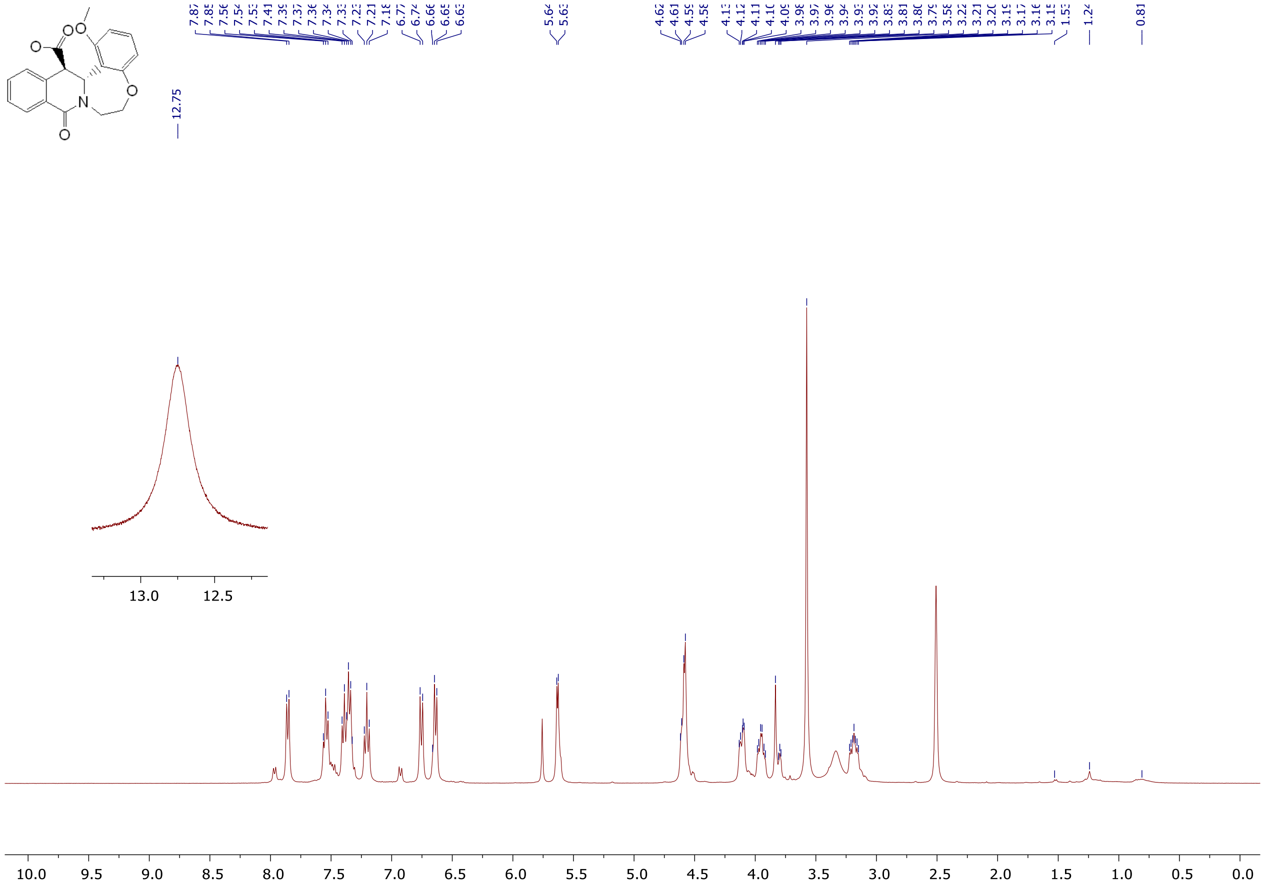

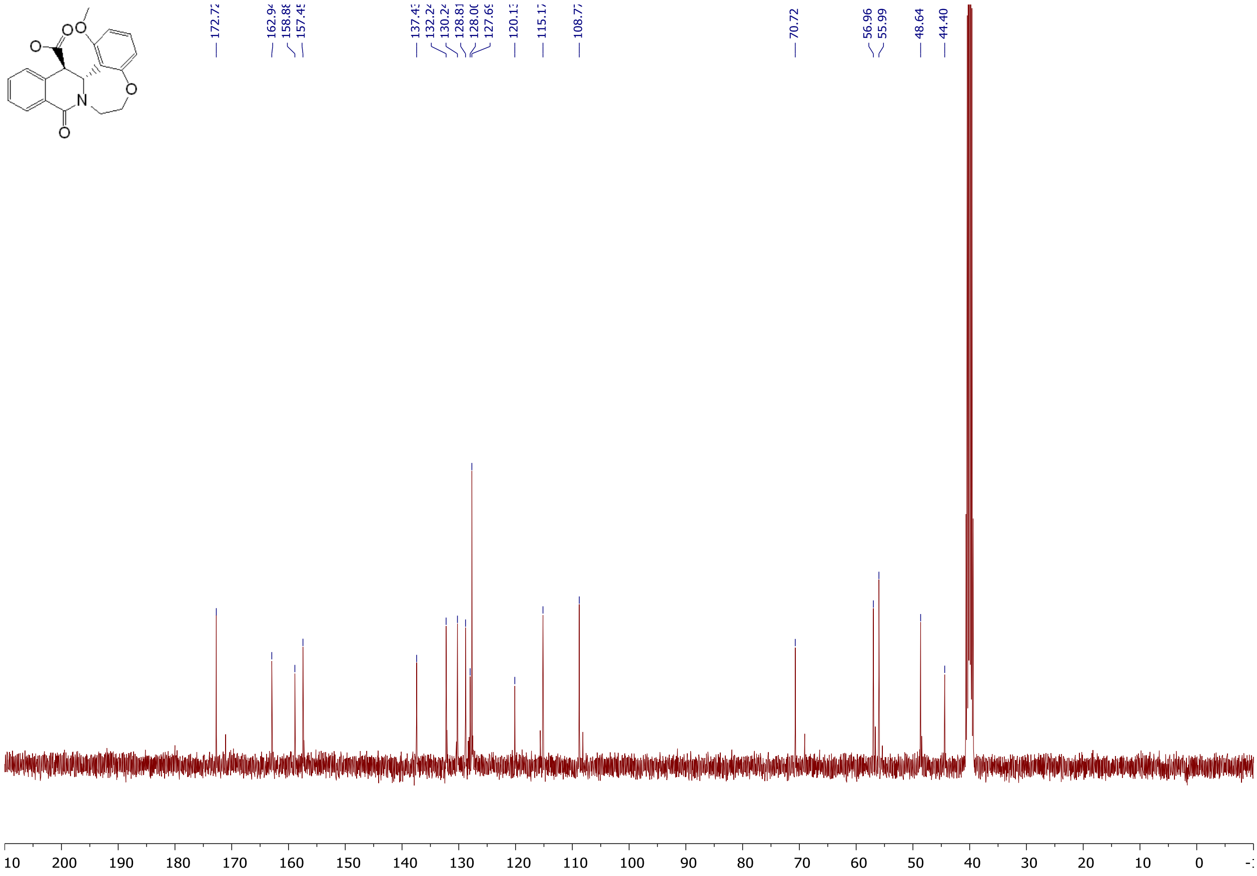

Copies of ^{1}H 400.13 MHz, DMSO-d_6 and ^{13}C 100.61 MHz, DMSO-d_6 spectra of **6l**

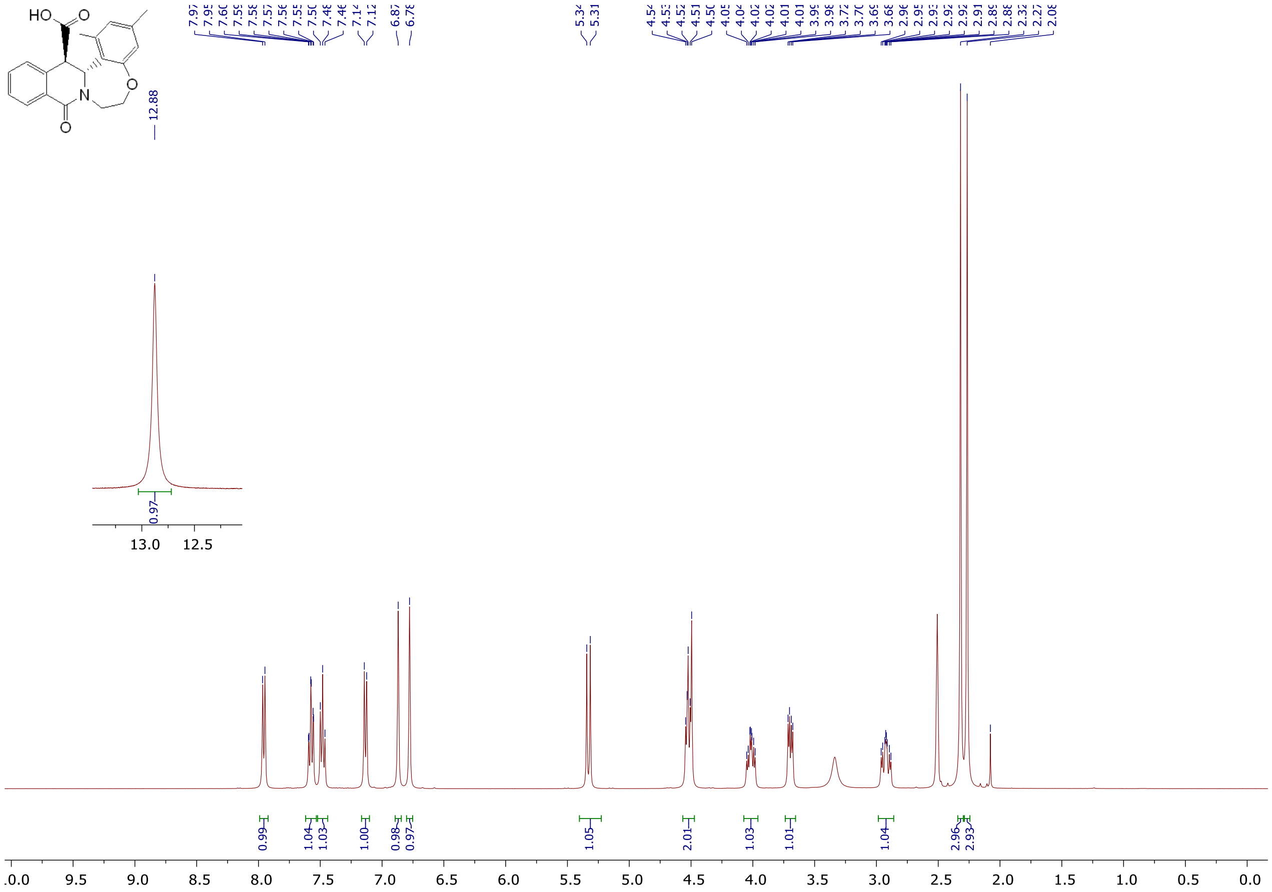

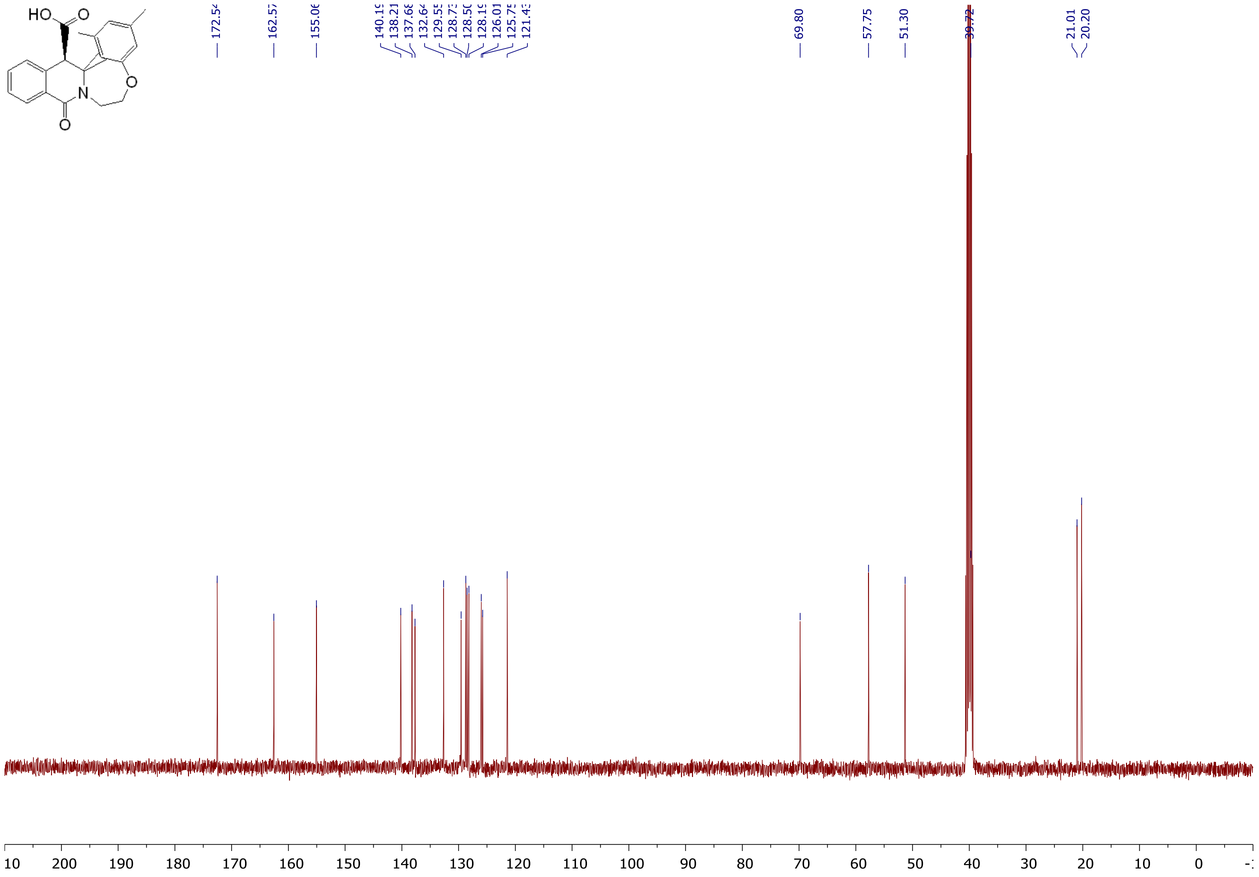

Copies of ^{1}H 400.13 MHz, DMSO-d_6 and ^{13}C 100.61 MHz, DMSO-d_6 spectra of **6m**

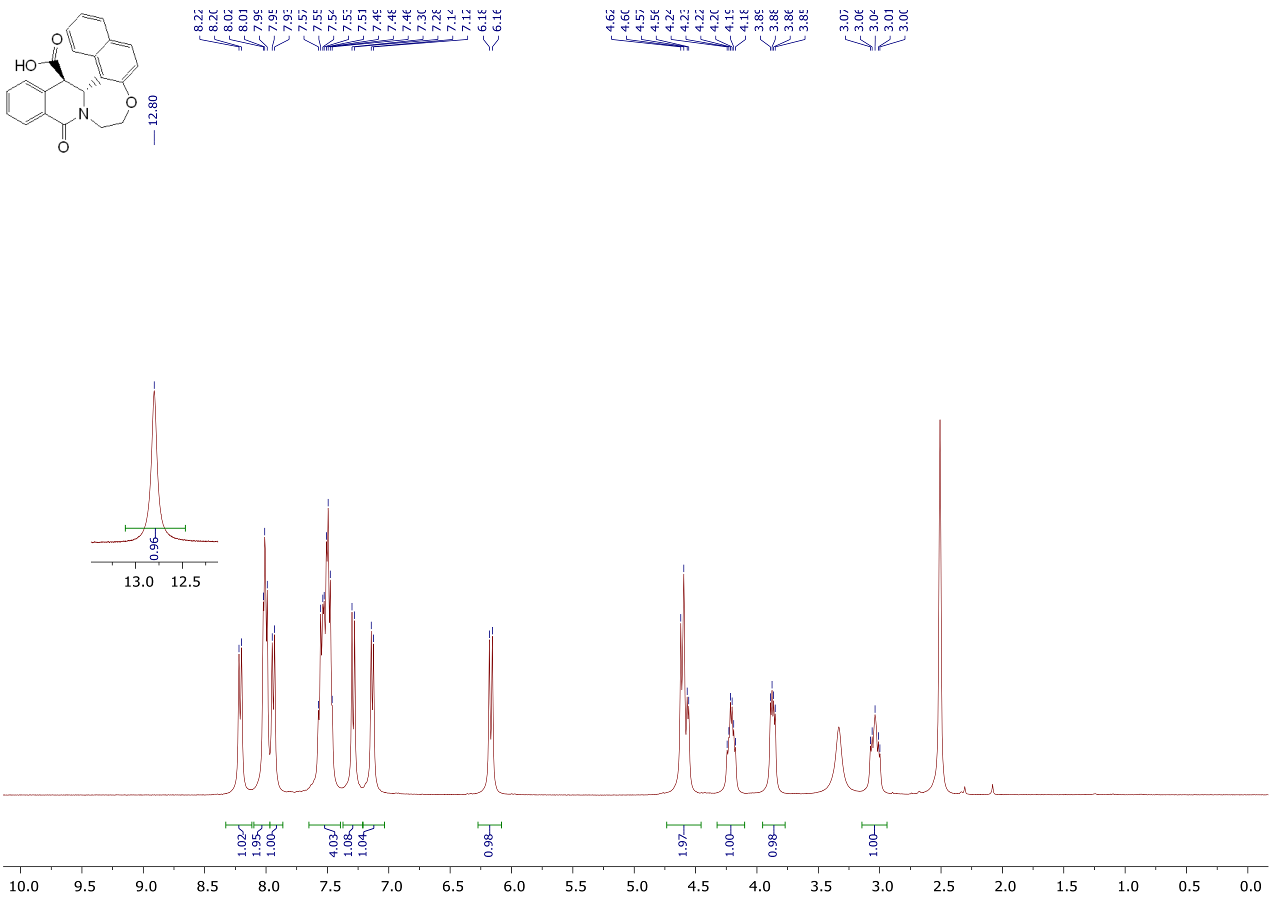

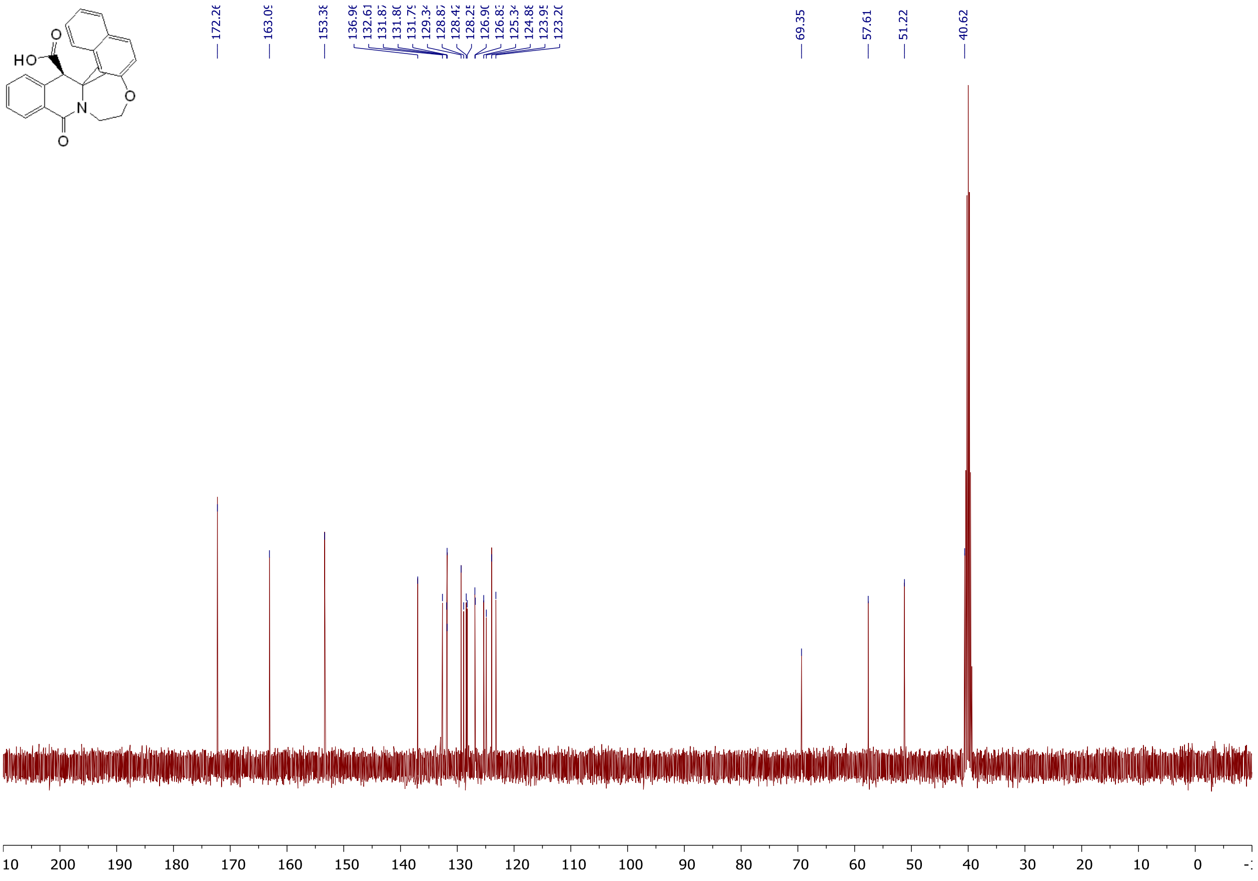

Copies of ^{1}H 400.13 MHz, $CDCl_3$ and ^{13}C 100.61 MHz, $CDCl_3$ spectra of **7**

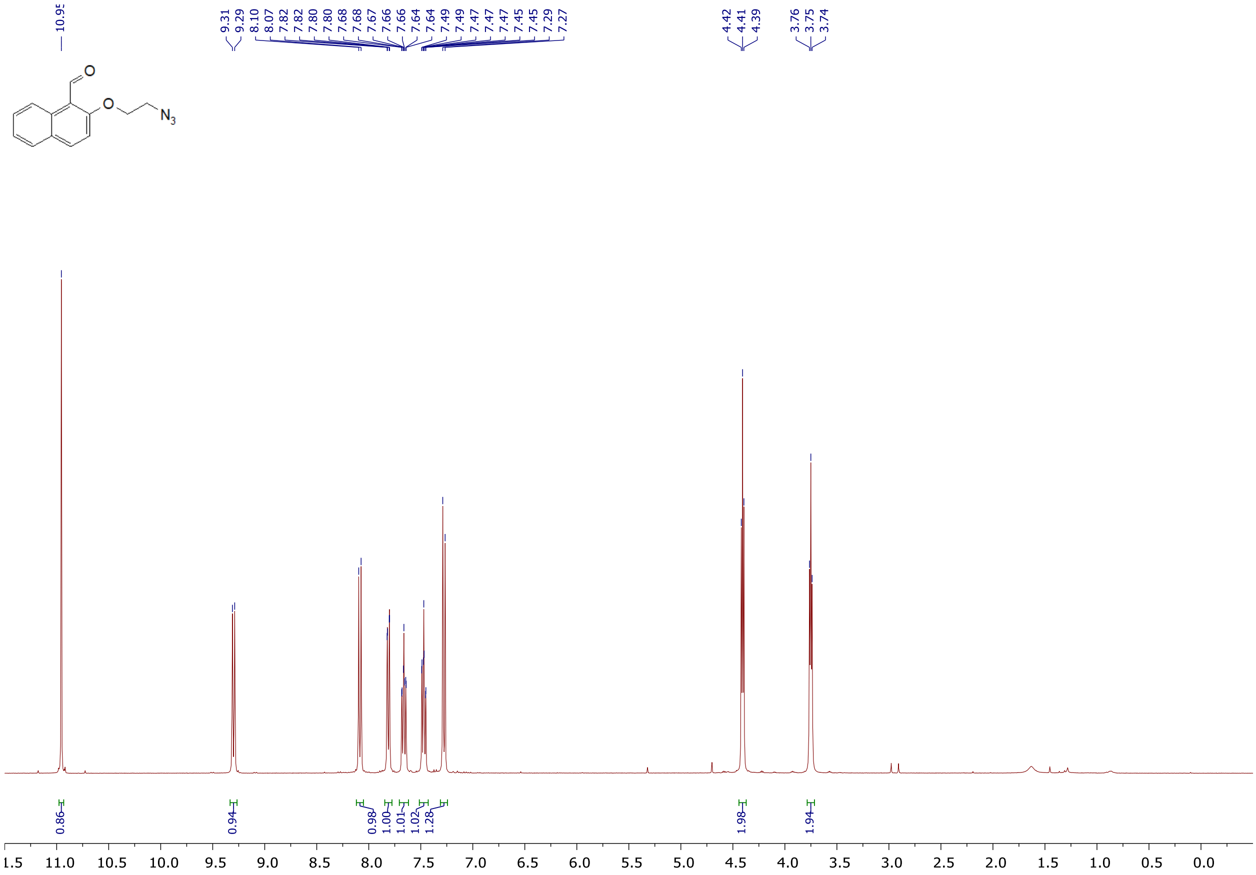

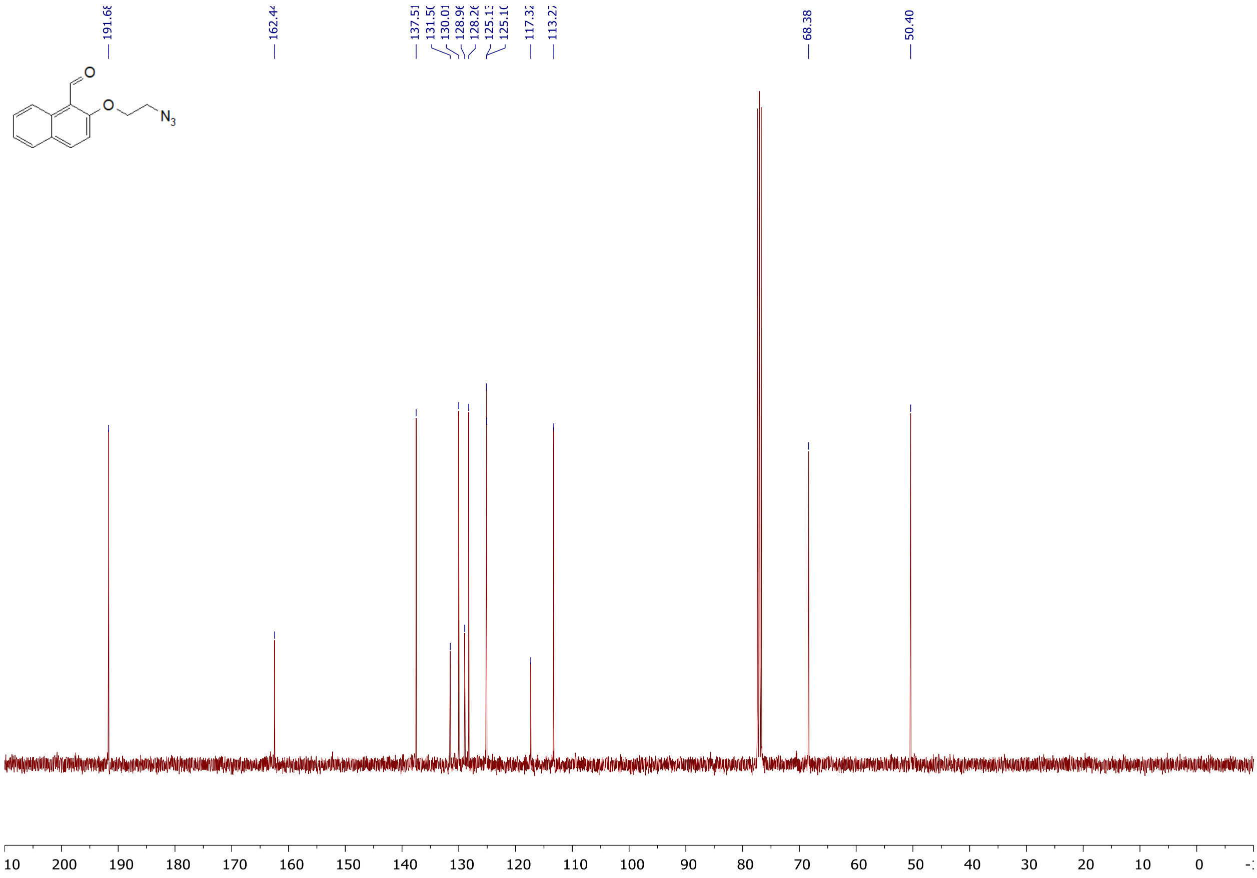

Copies of ^{1}H 400.13 MHz, $CDCl_3$ and ^{13}C 100.61 MHz, $CDCl_3$ spectra of **8**

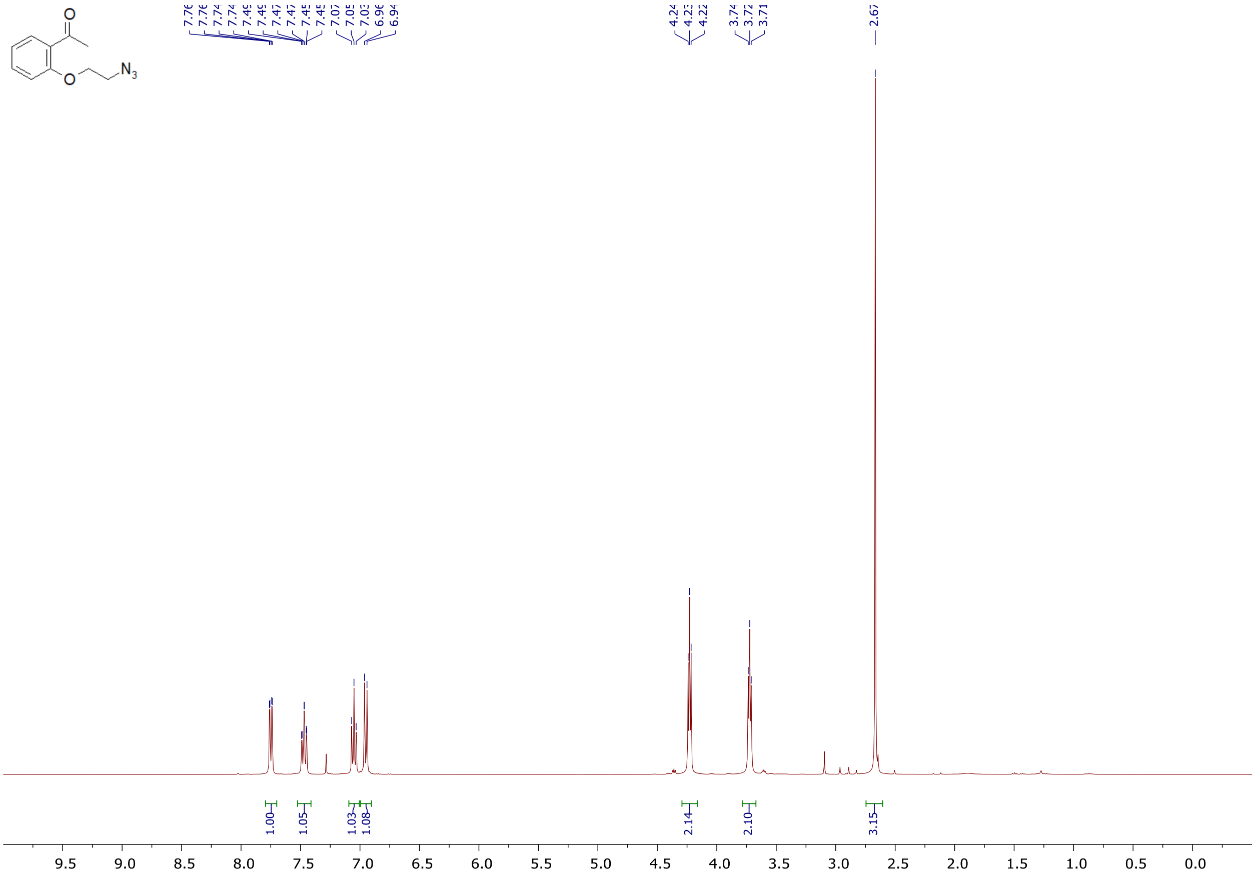

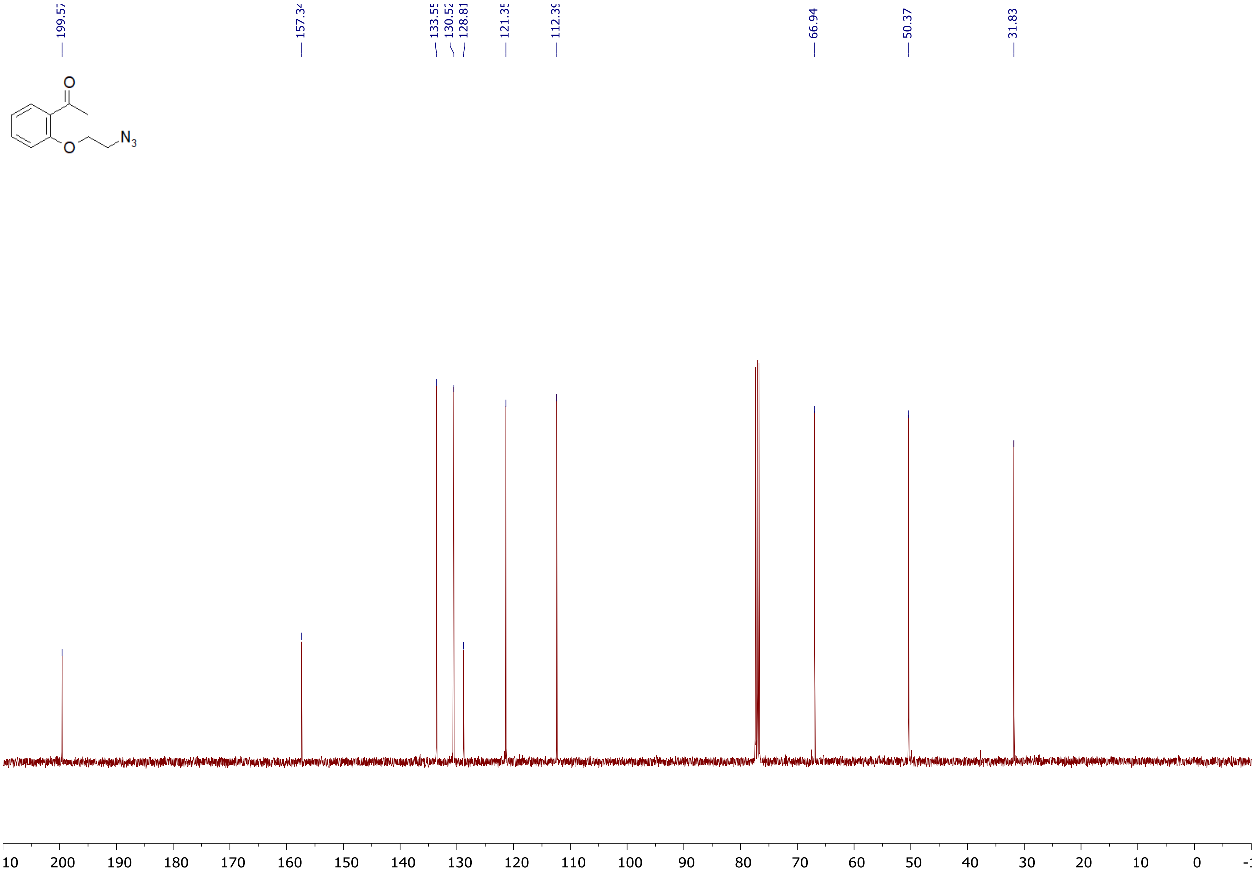

Copies of ^{1}H 400.13 MHz, $CDCl_3$ and ^{13}C 100.61 MHz, $CDCl_3$ spectra of **10a**

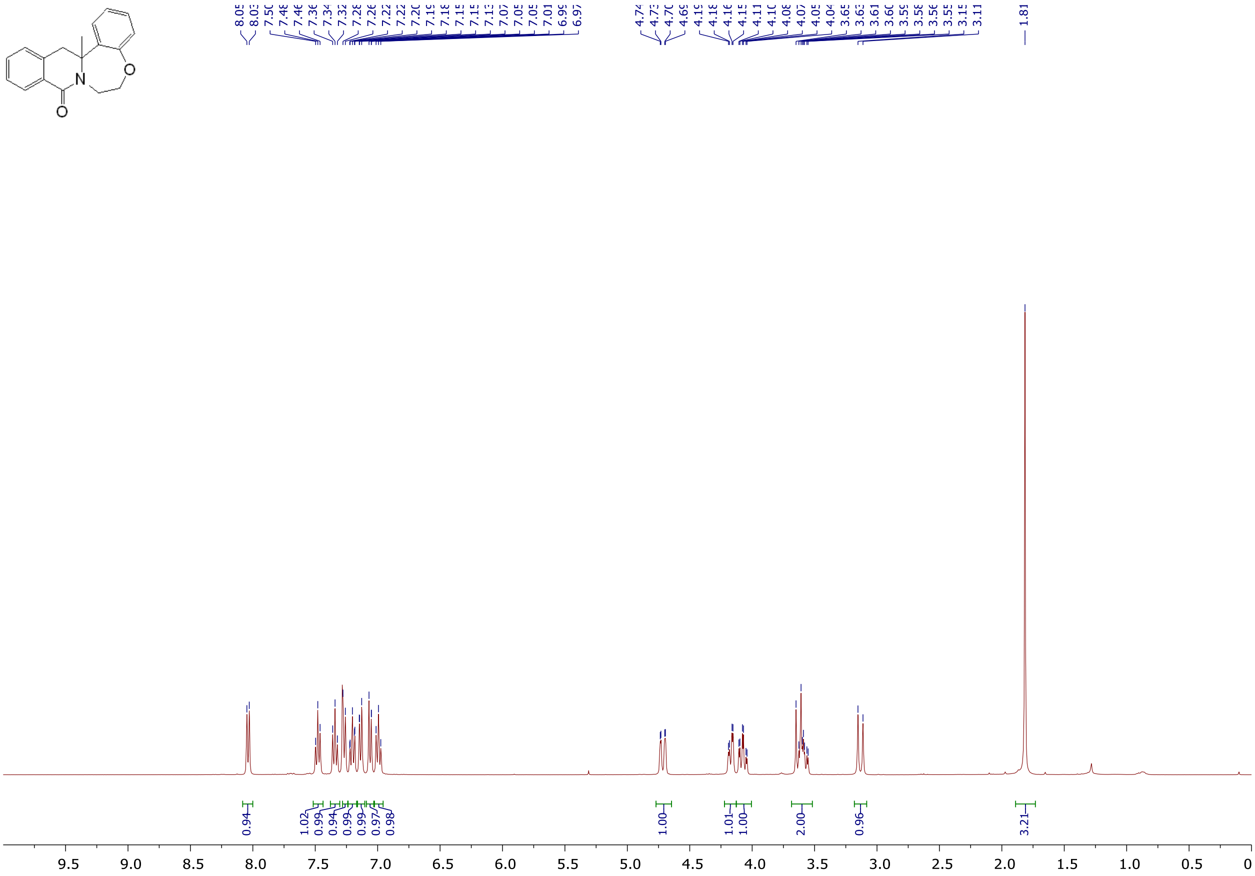

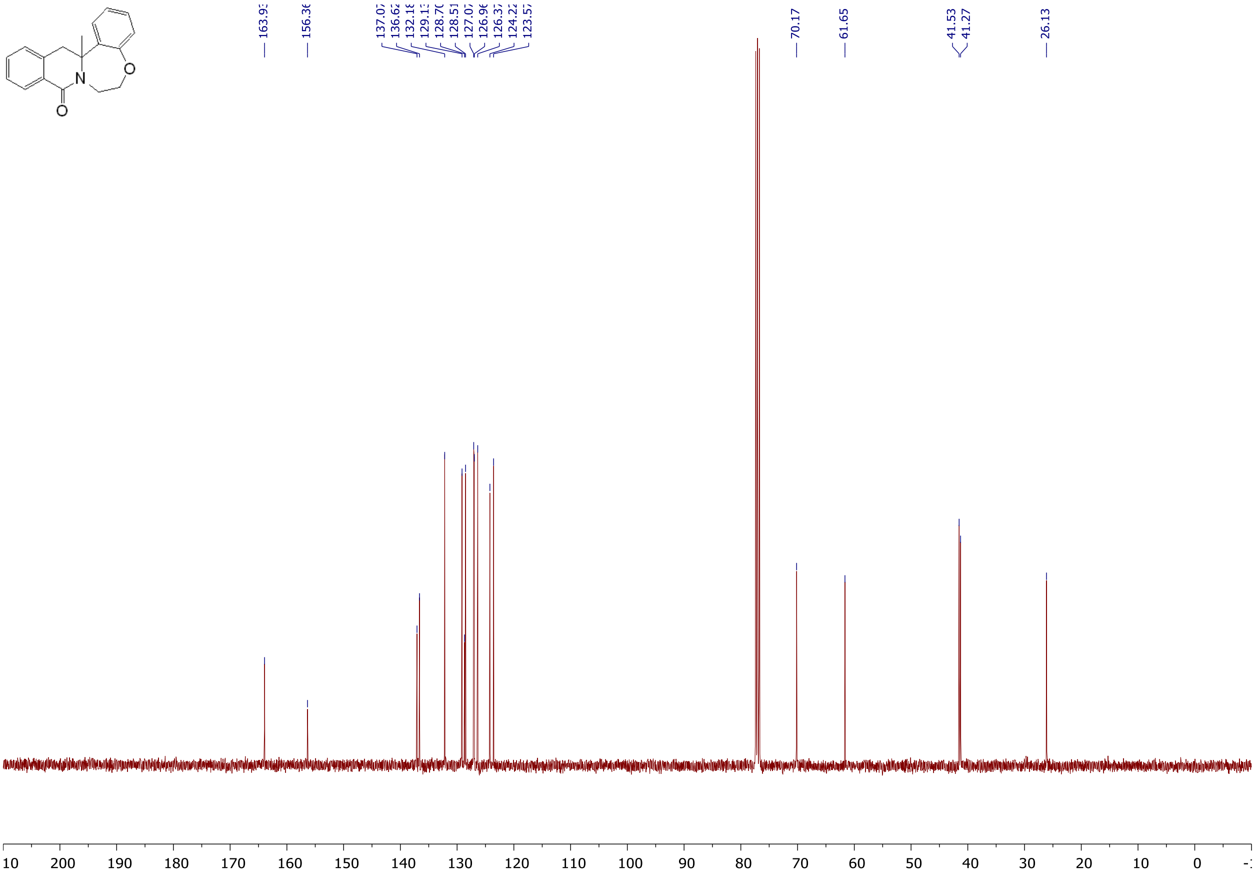

Copies of ^{1}H 400.13 MHz, $CDCl_3$ and ^{13}C 100.61 MHz, $CDCl_3$ spectra of **10b**

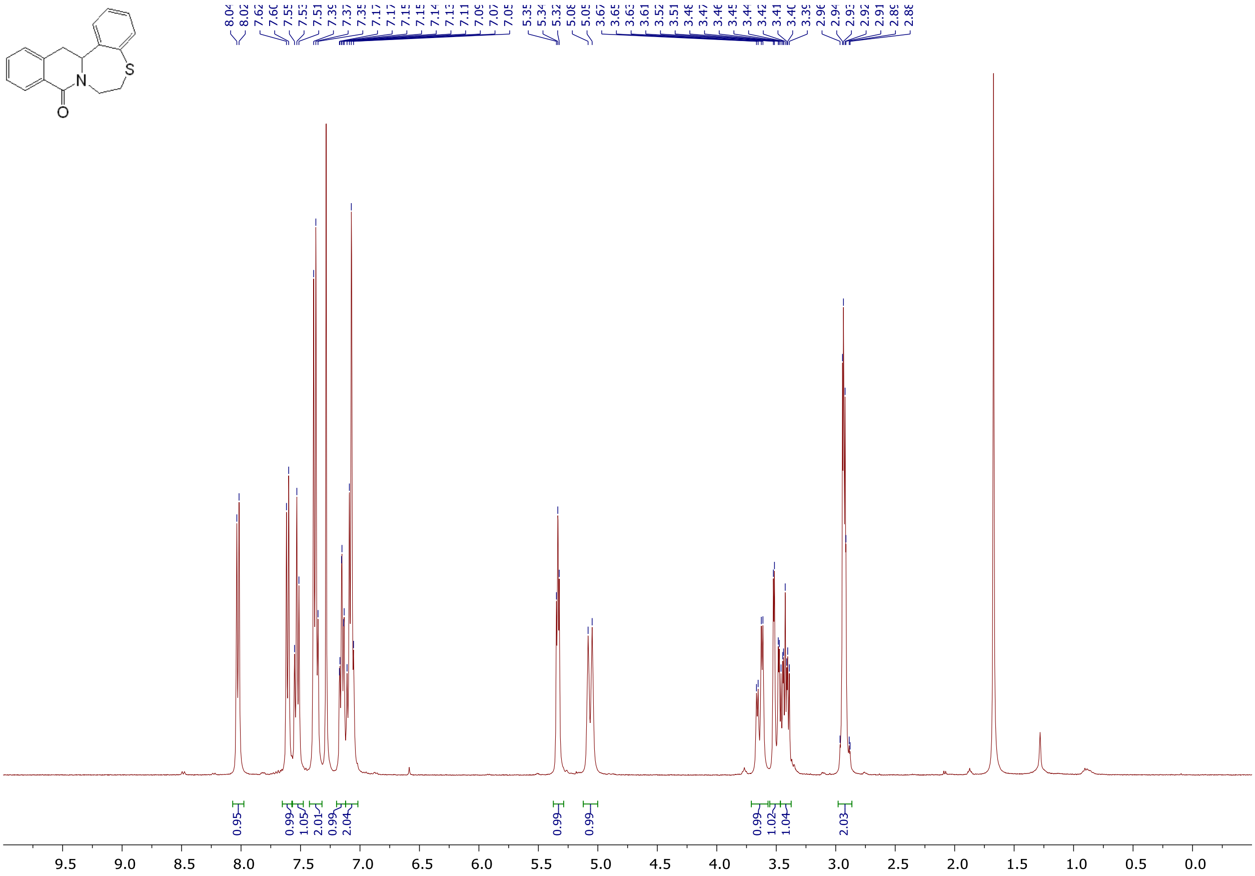

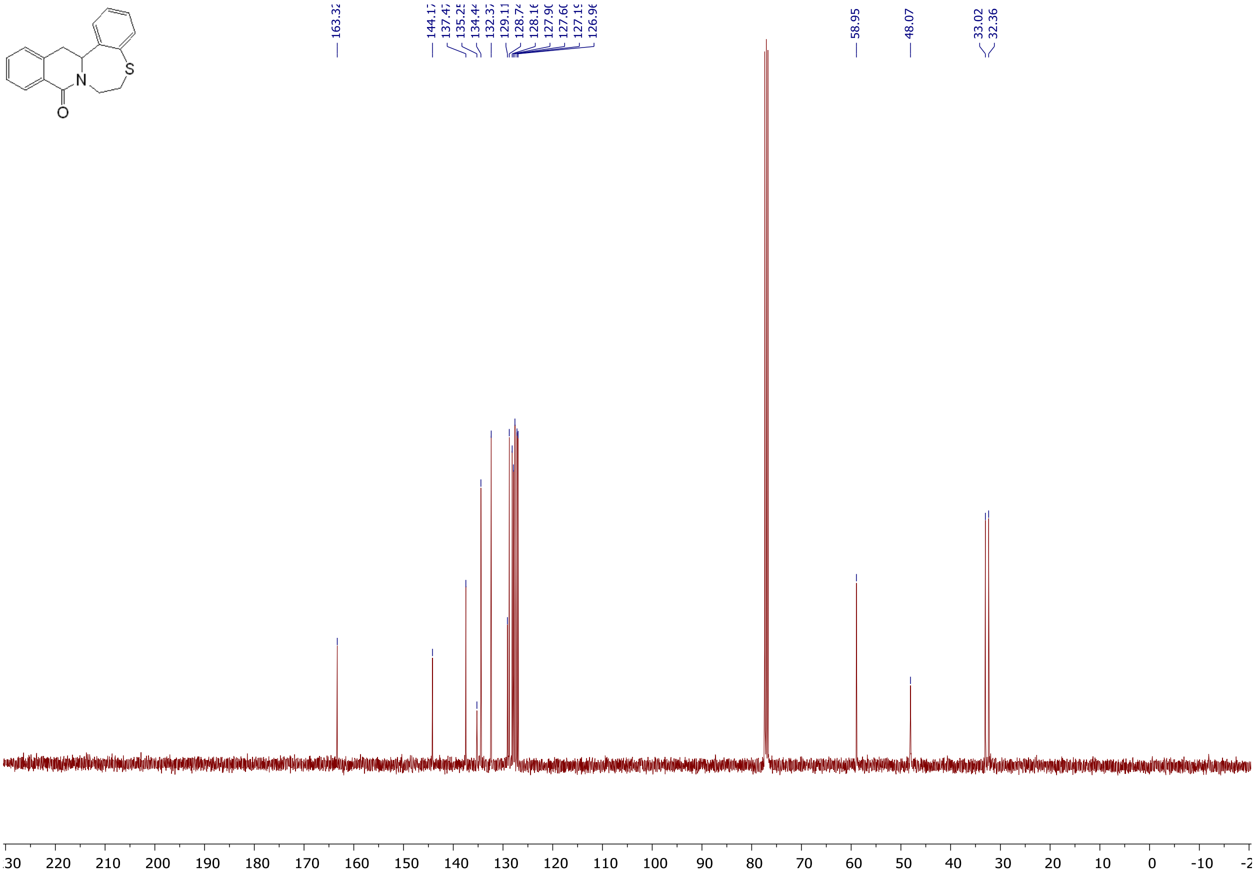

Copies of ^{1}H 400.13 MHz, $CDCl_3$ and ^{13}C 100.61 MHz, $CDCl_3$ spectra of **10c**

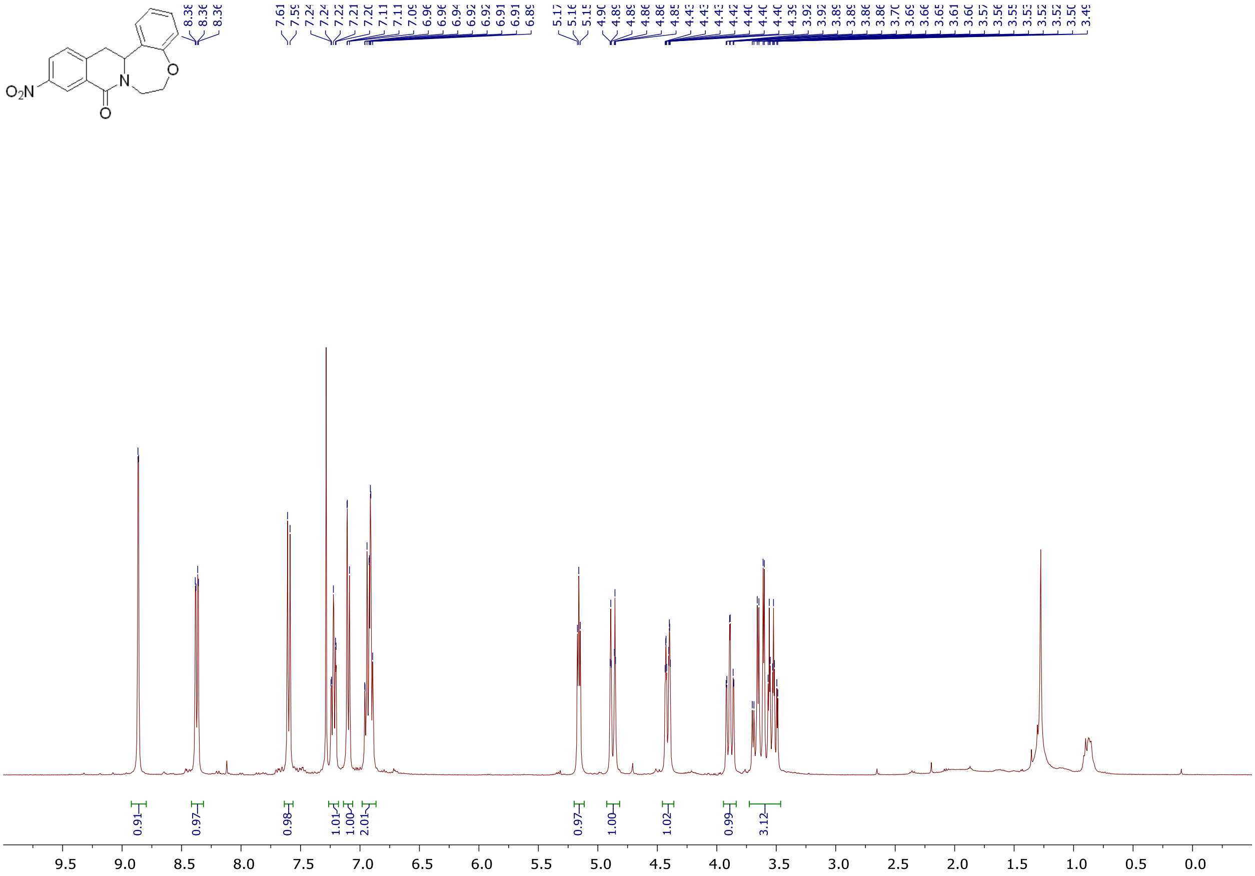

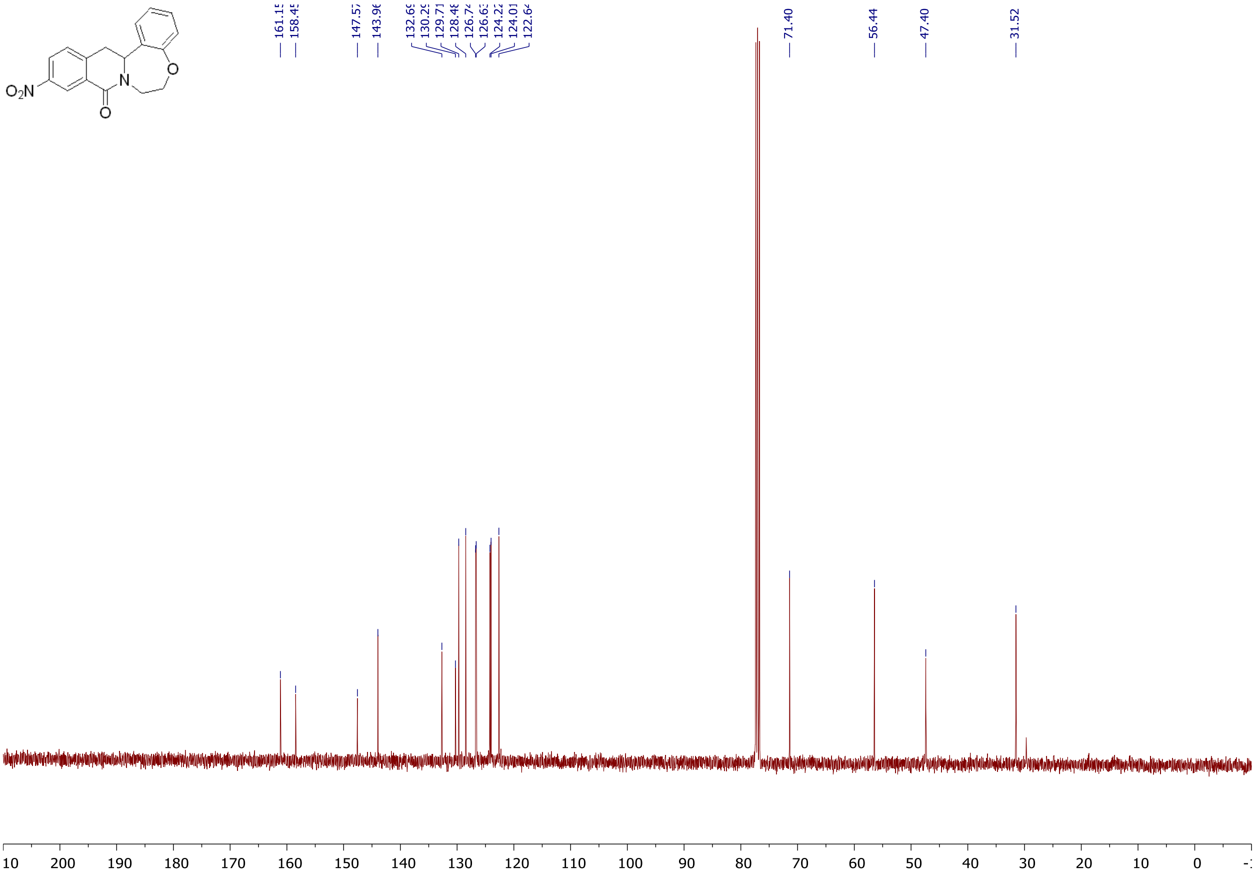

Copies of ^{1}H 400.13 MHz, $CDCl_3$ and ^{13}C 100.61 MHz, $CDCl_3$ spectra of **10d**

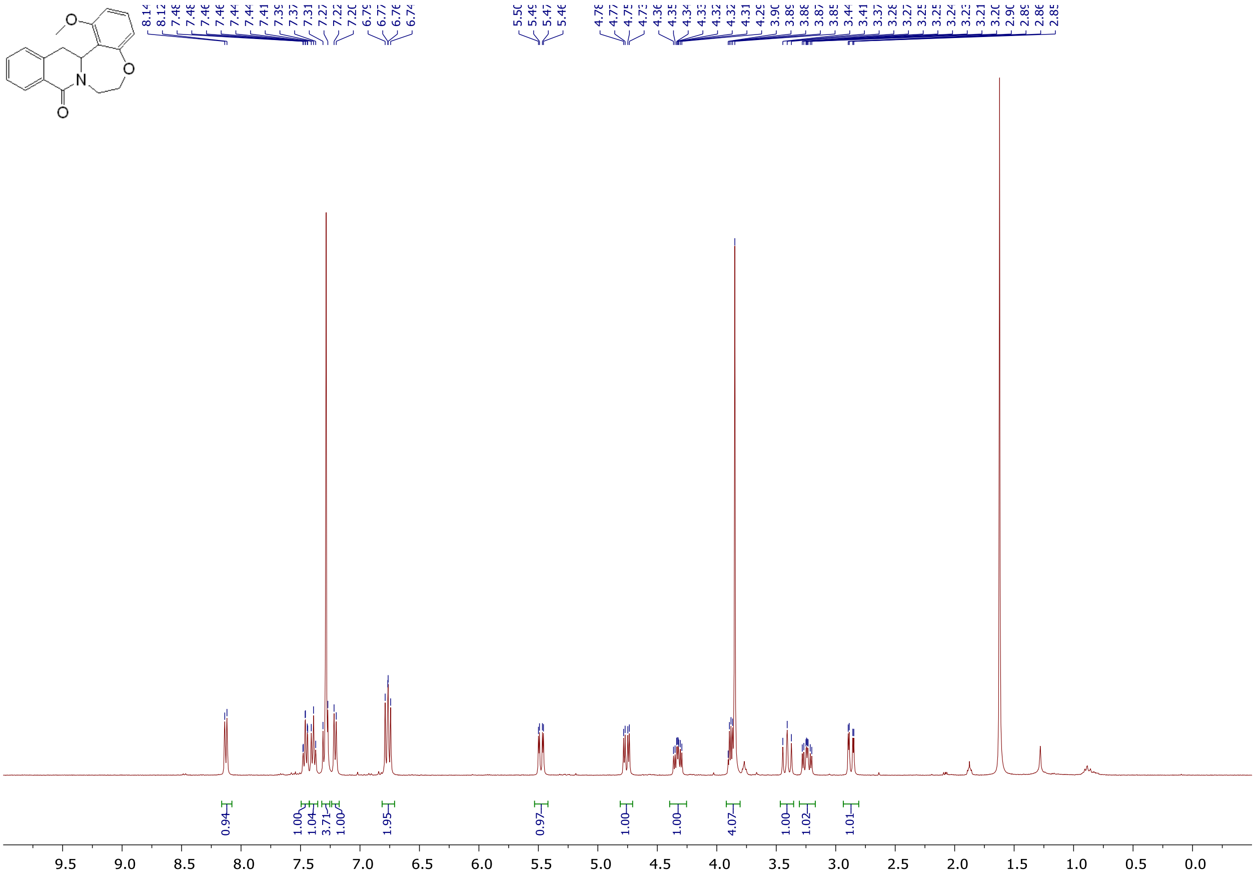

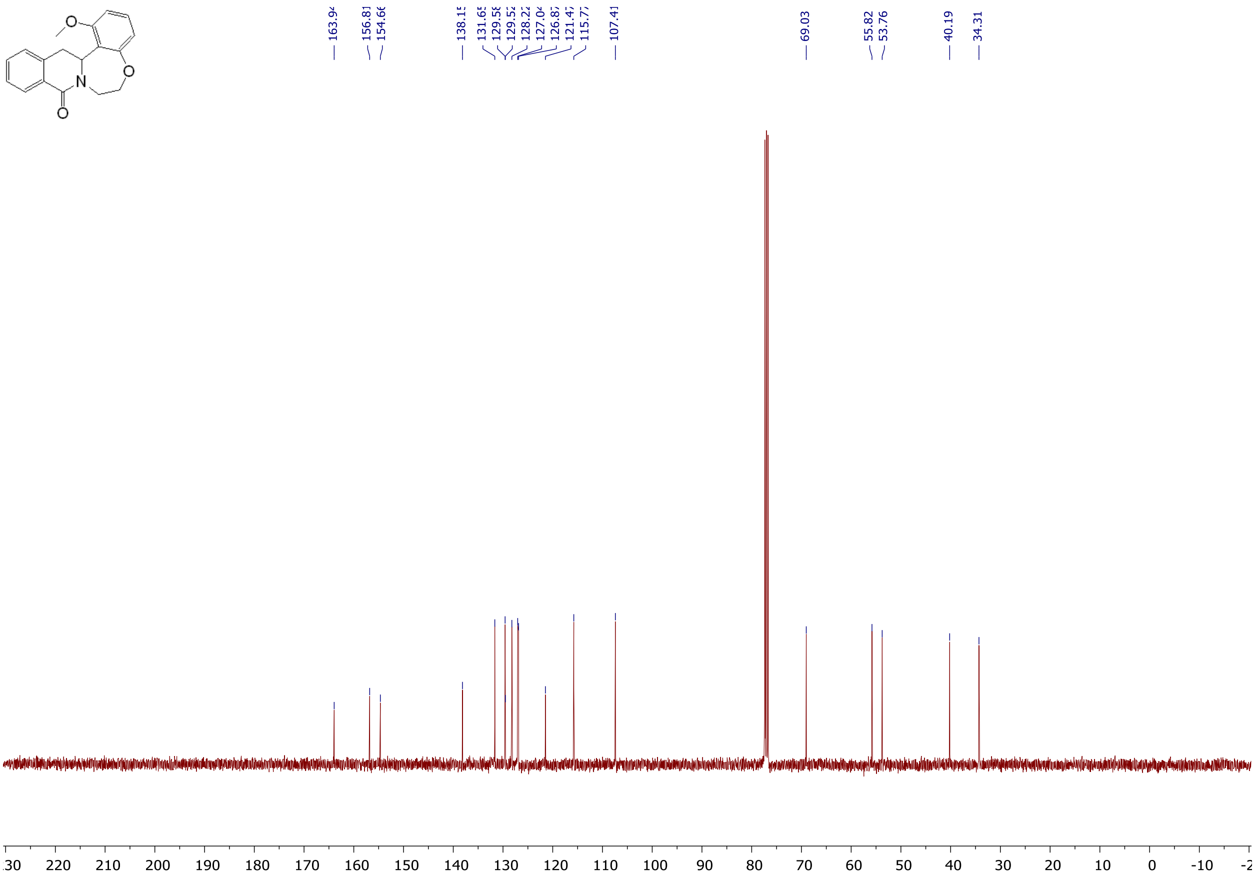

Copies of ^{1}H 400.13 MHz, $CDCl_3$ and ^{13}C 100.61 MHz, $CDCl_3$ spectra of **10e**

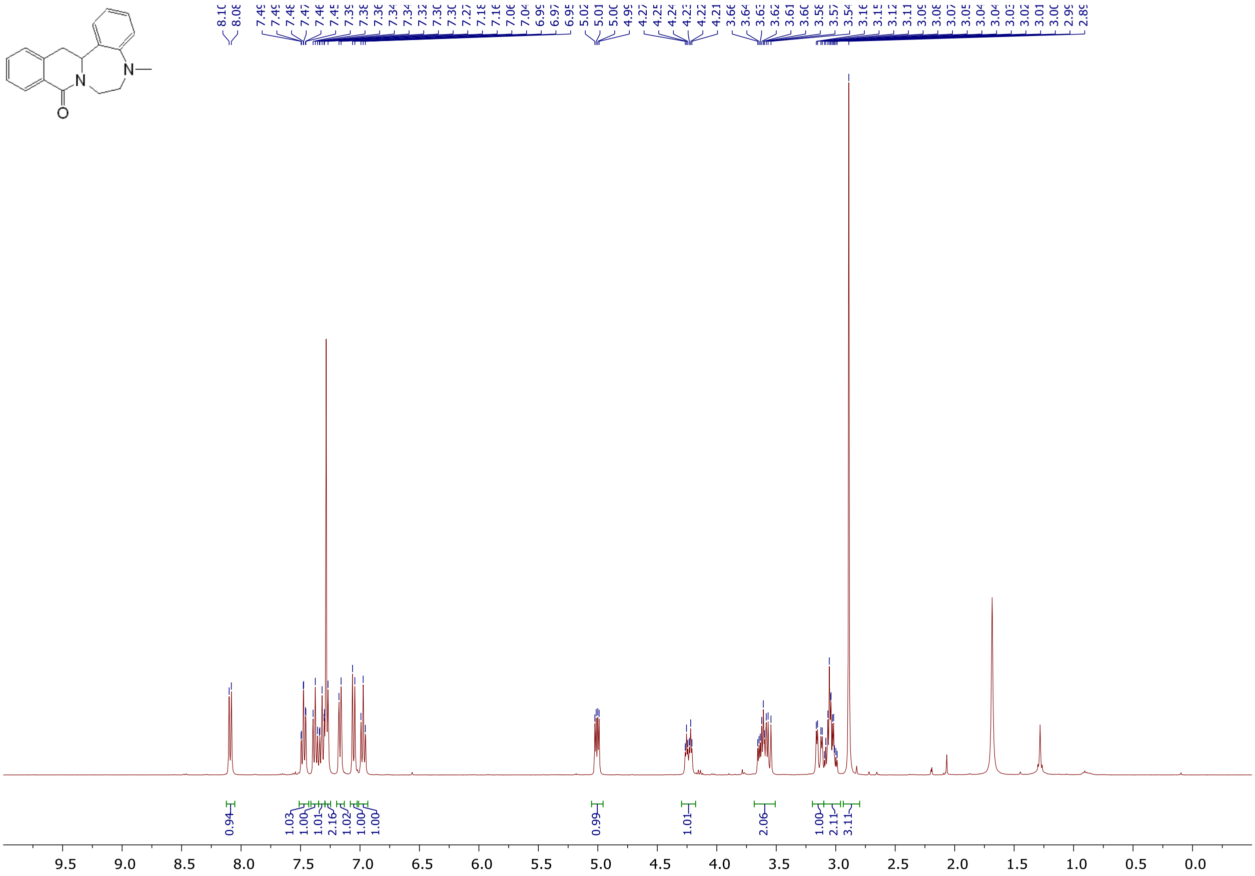

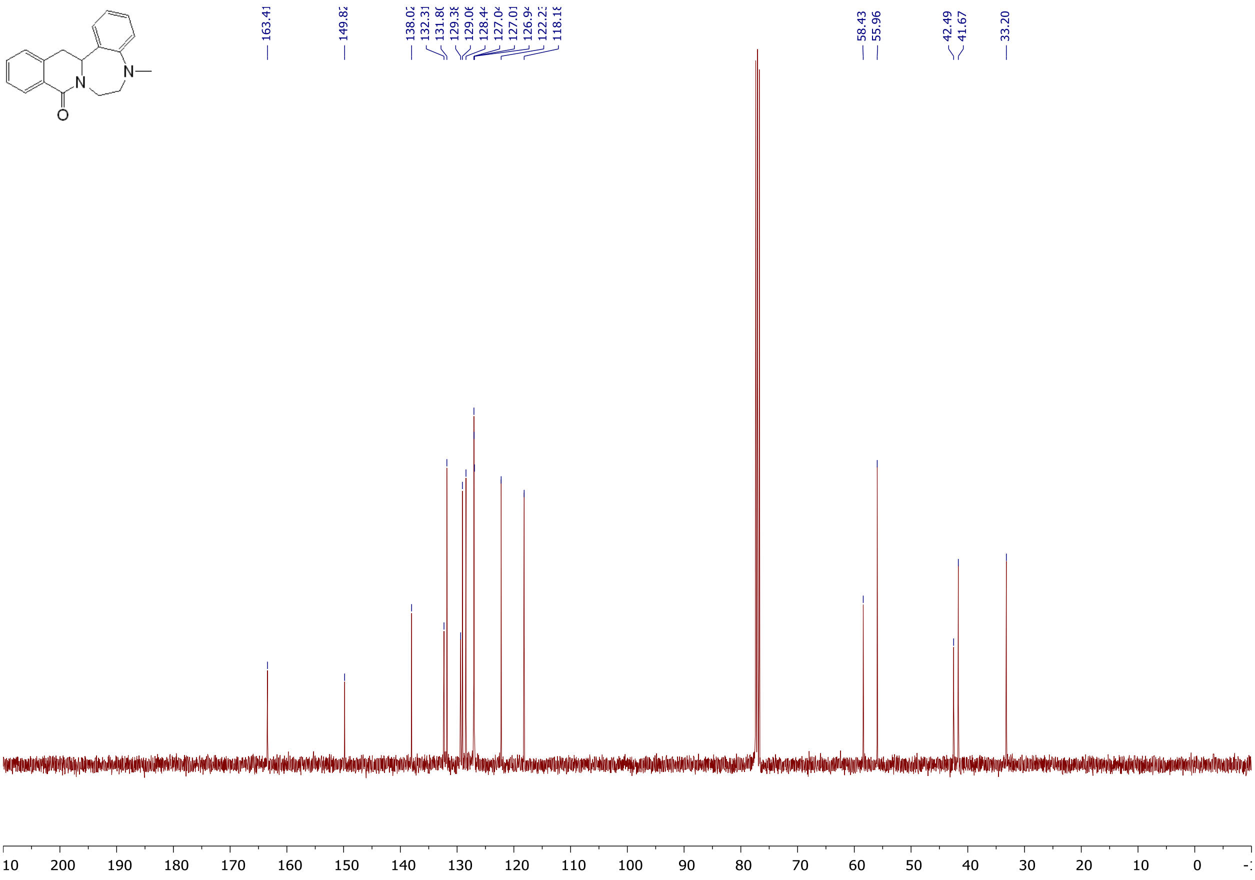

Copies of ^{1}H 400.13 MHz, DMSO-d_6 and ^{13}C 100.61 MHz, DMSO-d_6 spectra of **13**

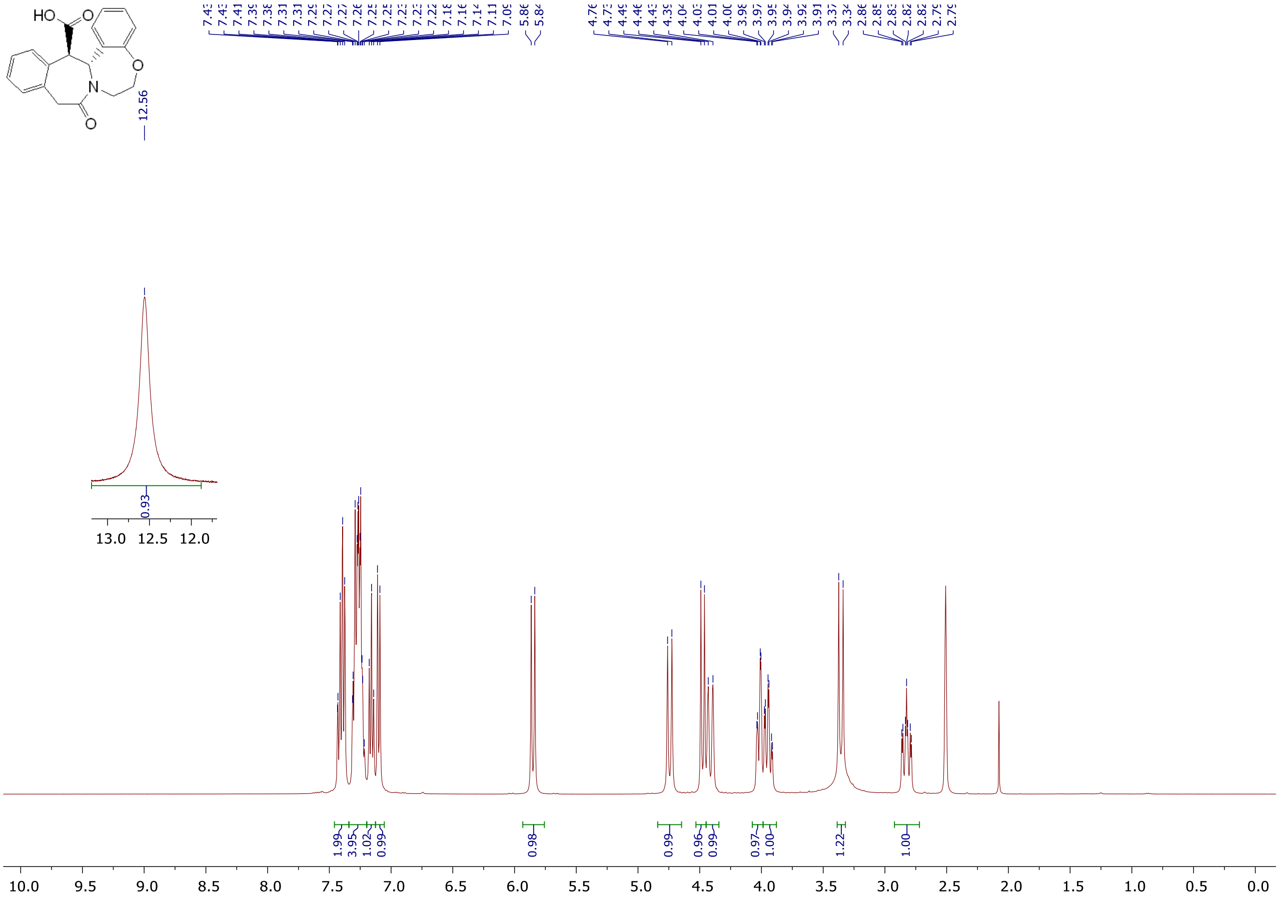

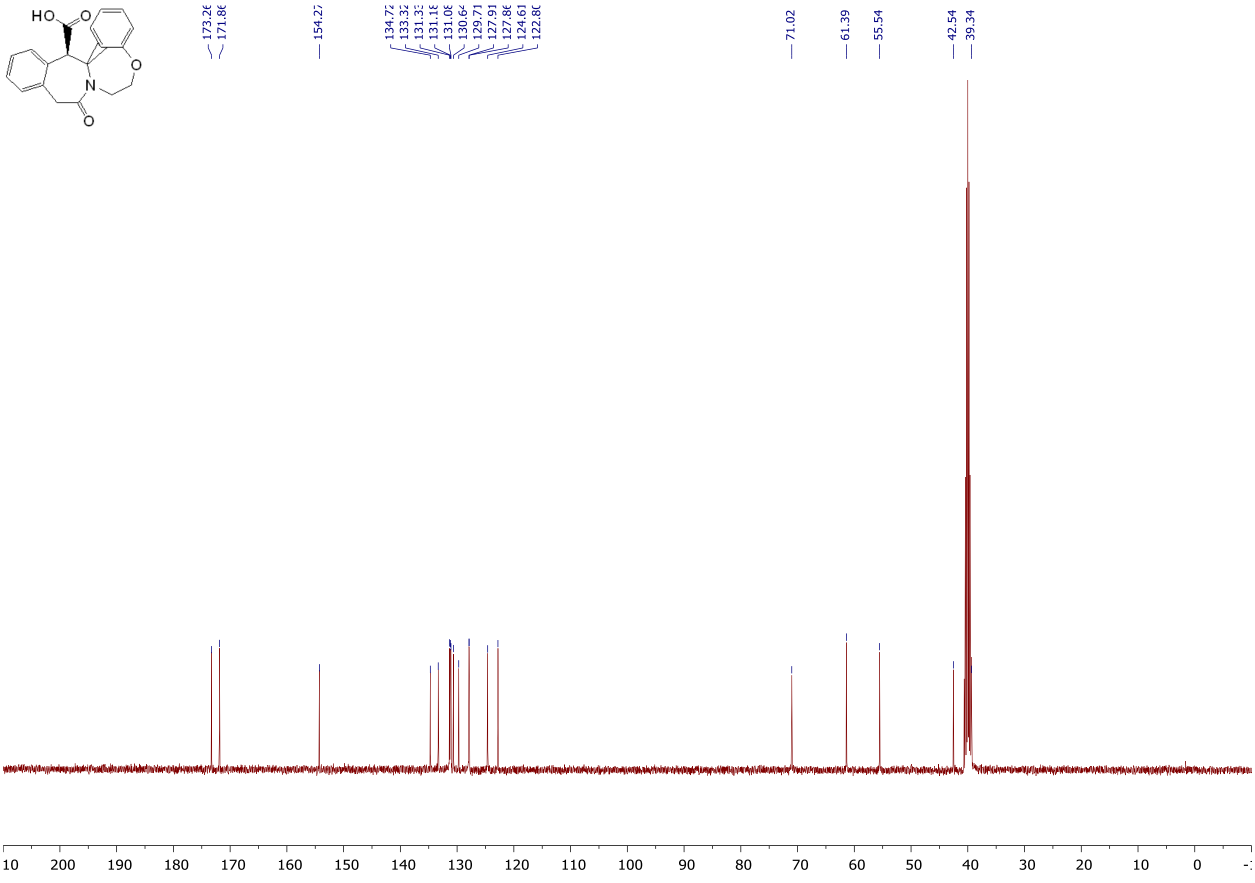

Copies of ^{1}H 400.13 MHz, DMSO-d_6 and ^{13}C 100.61 MHz, DMSO-d_6 spectra of **16**

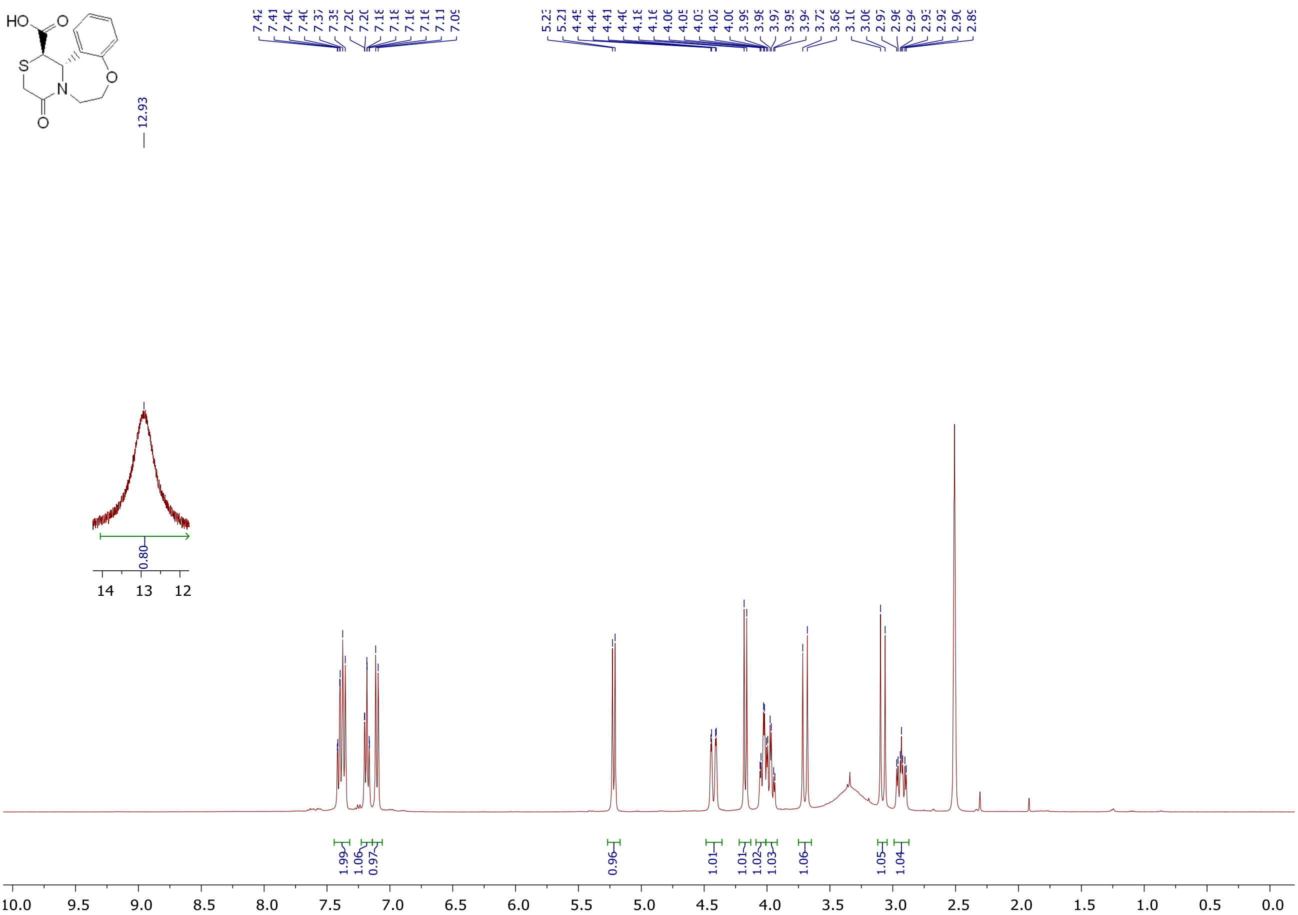

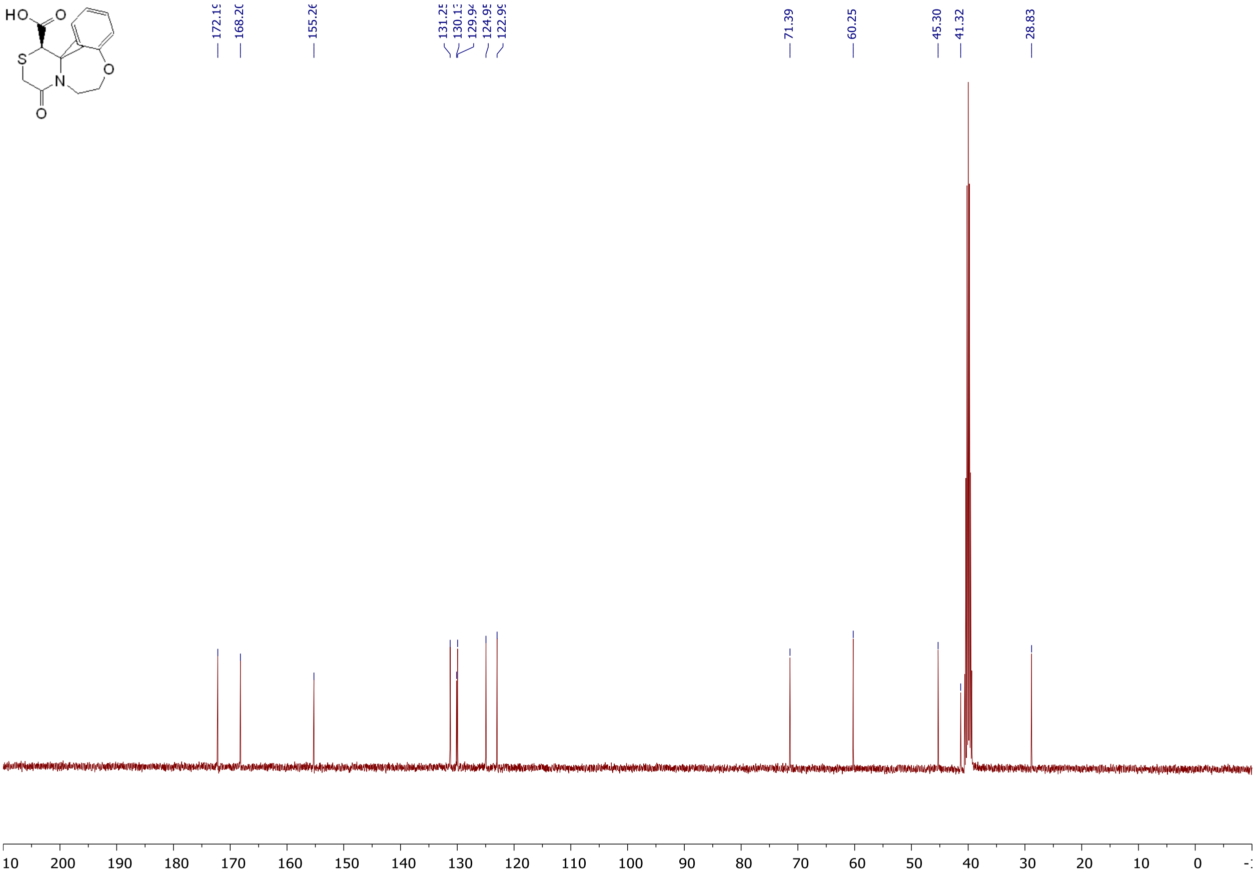

Copies of ^{1}H 400.13 MHz, $CDCl_3$ and ^{13}C 100.61 MHz, $CDCl_3$ spectra of **17**

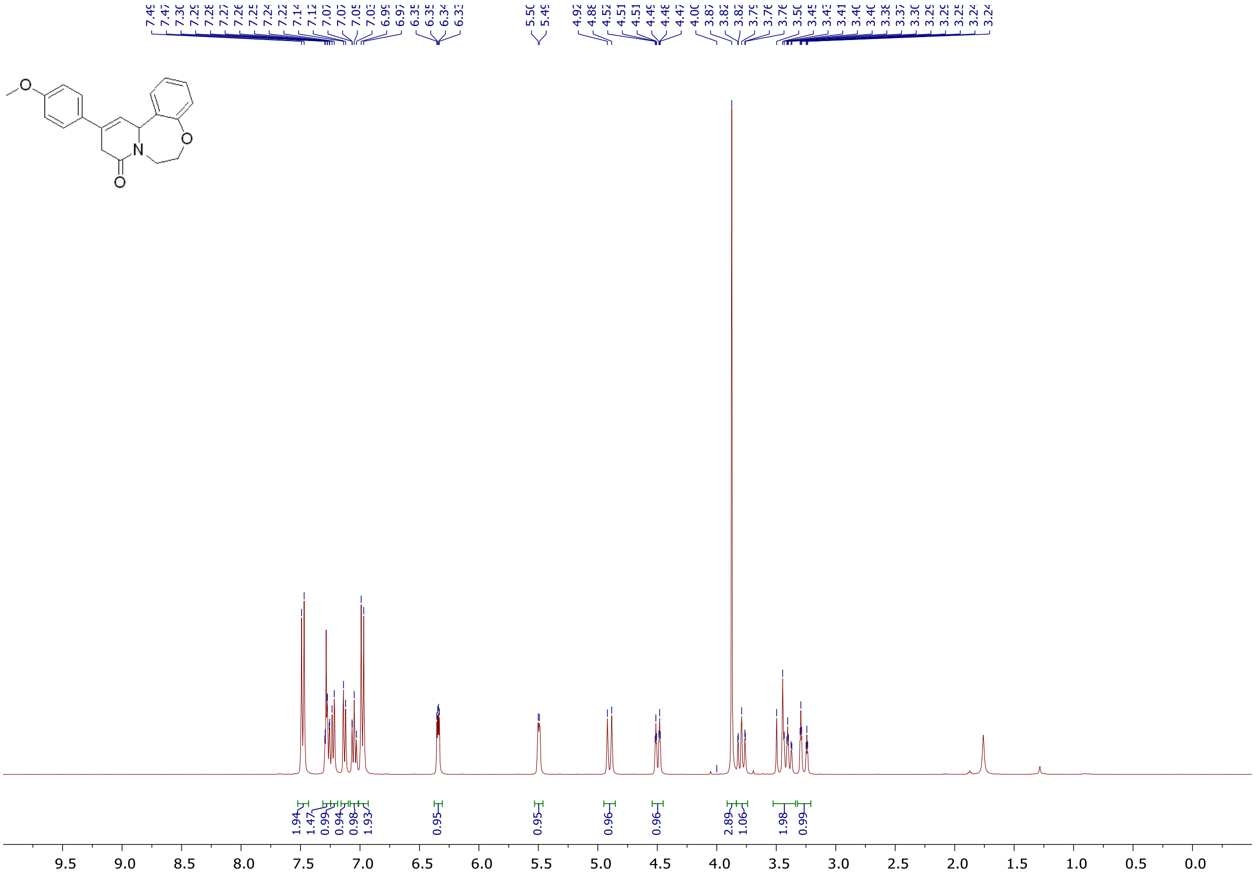

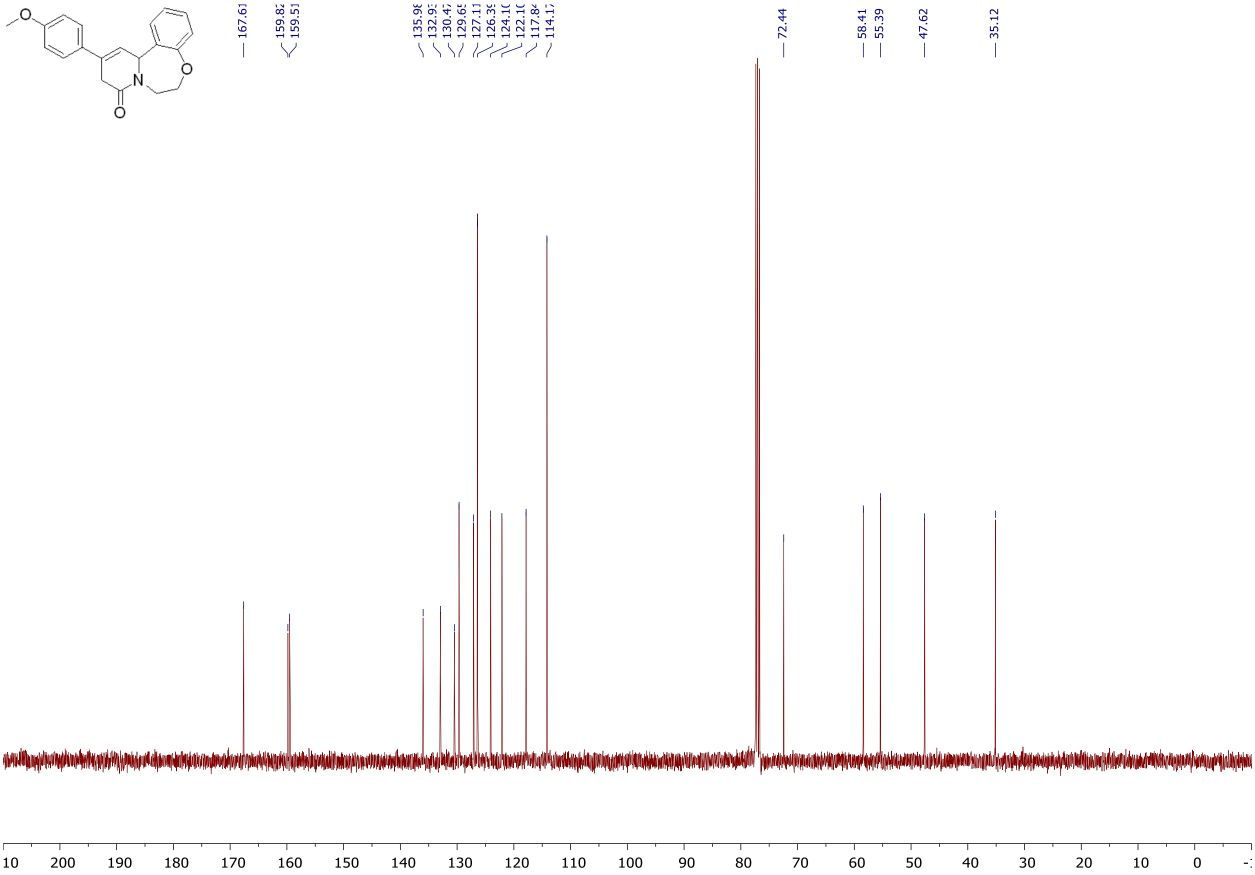

Supplement: Supplementary file 1 [file molecules-27-08130-s001.zip › molecules-2016631-supplementary.pdf]
